# A metabolic signature for NADSYN1-dependent congenital NAD deficiency disorder

**DOI:** 10.1172/JCI174824

**Published:** 2024-02-15

**Authors:** Justin O. Szot, Hartmut Cuny, Ella M.M.A. Martin, Delicia Z. Sheng, Kavitha Iyer, Stephanie Portelli, Vivien Nguyen, Jessica M. Gereis, Dimuthu Alankarage, David Chitayat, Karen Chong, Ingrid M. Wentzensen, Catherine Vincent-Delormé, Alban Lermine, Emma Burkitt-Wright, Weizhen Ji, Lauren Jeffries, Lynn S. Pais, Tiong Y. Tan, James Pitt, Cheryl A. Wise, Helen Wright, Israel D. Andrews, Brianna Pruniski, Theresa A. Grebe, Nicole Corsten-Janssen, Katelijne Bouman, Cathryn Poulton, Supraja Prakash, Boris Keren, Natasha J. Brown, Matthew F. Hunter, Oliver Heath, Saquib A. Lakhani, John H. McDermott, David B. Ascher, Gavin Chapman, Kayleigh Bozon, Sally L. Dunwoodie

**Affiliations:** 1 Victor Chang Cardiac Research Institute, Darlinghurst, Sydney, New South Wales, Australia.; 2 School of Clinical Medicine, Faculty of Medicine and Health, Sydney, New South Wales, Australia.; 3 School of Chemistry and Molecular Biosciences, University of Queensland, Brisbane, Queensland, Australia.; 4 Computational Biology and Clinical Informatics, Baker Heart and Diabetes Institute, Melbourne, Victoria, Australia.; 5 Department of Pediatrics, Division of Clinical and Metabolic Genetics, The Hospital for Sick Children, and; 6 Prenatal Diagnosis and Medical Genetics Program, Department of Obstetrics and Gynecology, Mount Sinai Hospital, University of Toronto, Toronto, Ontario, Canada.; 7 GeneDx, Gaithersburg, Maryland, USA.; 8 Clinique de Génétique “Guy Fontaine,” Hôpital Jeanne de Flandre, Lille, France.; 9 Laboratoire de Biologie Médicale Multisites SeqOIA, FMG2025, Paris, France.; 10 Manchester Centre for Genomic Medicine, St. Mary’s Hospital, Manchester University Hospitals NHS Foundation Trust, Manchester, United Kingdom.; 11 Yale University School of Medicine, Pediatric Genomics Discovery Program, New Haven, Connecticut, USA.; 12 Broad Institute of MIT and Harvard, Cambridge, Massachusetts, USA.; 13 Victorian Clinical Genetics Services, Murdoch Children’s Research Institute, Melbourne, Victoria, Australia.; 14 Department of Paediatrics, The University of Melbourne, Parkville, Victoria, Australia.; 15 Metabolic Laboratory, Victorian Clinical Genetics Services, Murdoch Children’s Research Institute, Melbourne, Victoria, Australia.; 16 Department of Diagnostic Genomics, PathWest Laboratory Medicine Western Australia, Nedlands, Perth, Western Australia, Australia.; 17 General Paediatric Department, Perth Children’s Hospital, Perth, Western Australia, Australia.; 18 Rural Clinical School, University of Western Australia, Perth, Western Australia, Australia.; 19 Pinnacle Dermatology, Scottsdale, Arizona, USA.; 20 Division of Genetics and Metabolism, Phoenix Children’s Hospital, Phoenix, Arizona, USA.; 21 Department of Genetics, University Medical Centre Groningen, University of Groningen, Groningen, Netherlands.; 22 Genetic Services of Western Australia, King Edward Memorial Hospital, Perth, Western Australia, Australia.; 23 Département de Génétique, Groupe Hospitalier Pitié-Salpêtrière, Assistance Publique – Hôpitaux de Paris, Sorbonne Université, Paris, France.; 24 Monash Genetics, Monash Health, Clayton, Victoria, Australia.; 25 Department of Paediatrics, Monash University, Clayton, Victoria, Australia.; 26 Department of Metabolic Medicine, The Royal Children’s Hospital, Melbourne, Victoria, Australia.; 27 Division of Evolution, Infection and Genomics, School of Biological Sciences, University of Manchester, Manchester, United Kingdom.; 28 Faculty of Science, University of New South Wales, Sydney, New South Wales, Australia.

**Keywords:** Reproductive biology, Therapeutics, Embryonic development, Genetic diseases, Mouse models

## Abstract

Nicotinamide adenine dinucleotide (NAD) is essential for embryonic development. To date, biallelic loss-of-function variants in 3 genes encoding nonredundant enzymes of the NAD de novo synthesis pathway — *KYNU*, *HAAO*, and *NADSYN1* — have been identified in humans with congenital malformations defined as congenital NAD deficiency disorder (CNDD). Here, we identified 13 further individuals with biallelic *NADSYN1* variants predicted to be damaging, and phenotypes ranging from multiple severe malformations to the complete absence of malformation. Enzymatic assessment of variant deleteriousness in vitro revealed protein domain–specific perturbation, complemented by protein structure modeling in silico. We reproduced NADSYN1-dependent CNDD in mice and assessed various maternal NAD precursor supplementation strategies to prevent adverse pregnancy outcomes. While for *Nadsyn1^+/–^* mothers, any B_3_ vitamer was suitable to raise NAD, preventing embryo loss and malformation, *Nadsyn1^–/–^* mothers required supplementation with amidated NAD precursors (nicotinamide or nicotinamide mononucleotide) bypassing their metabolic block. The circulatory NAD metabolome in mice and humans before and after NAD precursor supplementation revealed a consistent metabolic signature with utility for patient identification. Our data collectively improve clinical diagnostics of NADSYN1-dependent CNDD, provide guidance for the therapeutic prevention of CNDD, and suggest an ongoing need to maintain NAD levels via amidated NAD precursor supplementation after birth.

## Abstract 2

<p>NADSYN1-dependent Congenital NAD Deficiency Disorder is characterized by a consistent metabolic signature with utility for patient identification and preventative therapeutic options.</p>

## Graphical abstract

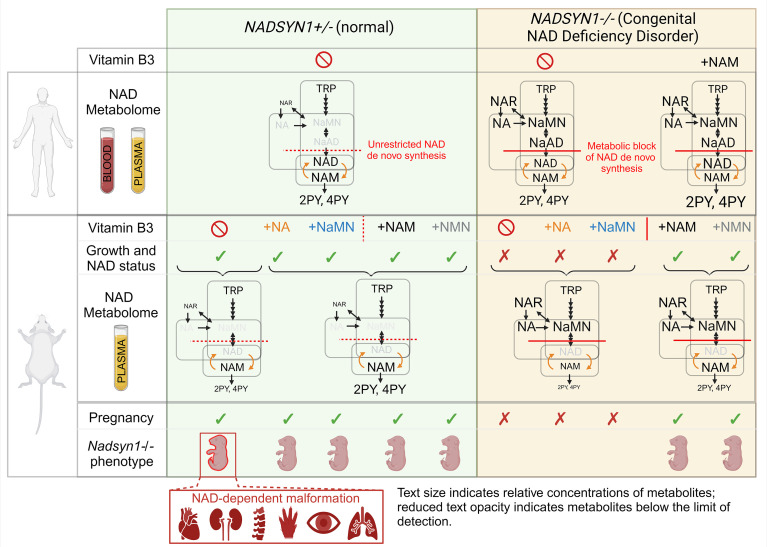

## Introduction

Nicotinamide adenine dinucleotide (NAD) is essential for embryonic development and is linked to some 488 different types of reactions in the human body (1). In mammalian cells, NAD is synthesized from l-tryptophan via the NAD de novo synthesis pathway or converted from dietary B__3__ vitamers, including nicotinic acid (NA) via the Preiss-Handler pathway, or nicotinamide (NAM), nicotinamide mononucleotide (NMN), and nicotinamide riboside (NR) via the salvage pathway (2) (Supplemental Figure 1; supplemental material available online with this article; https://doi.org/10.1172/JCI174824DS1). NAD availability is cell and tissue specific (3), and while most of the adult NAD pool is derived from NAD salvage rather than synthesis (4), homeostatic levels of NAD are maintained through consumption and replenishment (5). Consequently, deficiency or dysregulation of NAD levels across various tissues underlies myriad postnatal cardiometabolic, epigenetic, neurodegenerative, and aging-related disorders (5, 6). Gestational NAD deficiency in humans due to perturbation of NAD de novo synthesis causes a diverse array of congenital malformations, most frequently affecting the heart, kidney, vertebrae, and limbs, and is termed congenital NAD deficiency disorder (CNDD). All CNDD cases identified to date originate from biallelic loss-of-function variants in any of 3 nonredundant genes of the NAD de novo synthesis pathway — *KYNU* (kynureninase), *HAAO* (3-hydroxyanthranilate 3,4-dioxygenase), or *NADSYN1* (NAD synthetase 1) (OMIM 617661, 617660, and 618845, respectively) (7, 8). Phenotypes vary between CNDD patients (9) and do not appear proportional to the enzymatic activity of their respective protein variants.

Mouse models of *Kynu*- and *Haao*-related CNDD recapitulate malformations seen in humans, which can be prevented in mice when the NAD precursor NA is provided via the Preiss-Handler pathway during pregnancy (8). In wild-type mice, NAD-dependent malformations can also be generated in embryos when the maternal diet contains insufficient NAD precursors, and combination of such dietary insufficiency with *Haao* heterozygosity exacerbates the phenotype via a gene-environment interaction (10).

Downstream of KYNU and HAAO, NADSYN1 directly generates NAD as the common final step of both the NAD de novo synthesis and Preiss-Handler pathways (Supplemental Figure 1). Among individuals with CNDD identified so far, those with biallelic pathogenic *NADSYN1* variants are the most diverse in phenotype (7, 9, 11–13). To better understand the impact of *NADSYN1* variation in humans and to explore the preventative potential of B__3__ vitamer supplementation on phenotype and the NAD metabolome, we report on a further 13 individuals from 10 unrelated families and a novel *Nadsyn1*-null mouse model of CNDD.

## Results

### *NADSYN1* variants affect enzymatic activity and phenotype in humans

**Figure 1:**
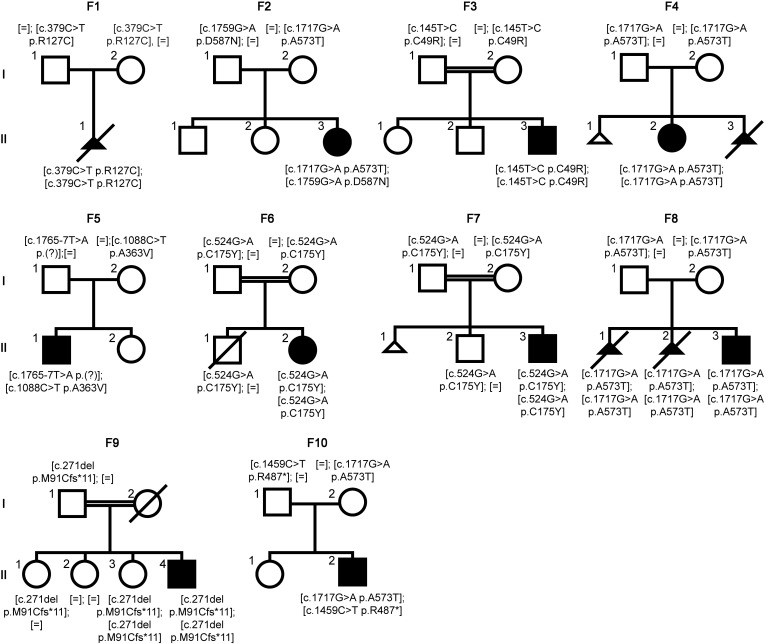
Pedigrees of families with individuals harboring biallelic *NADSYN1* variants. Squares indicate male individuals, circles female, triangles first-trimester deaths, solid symbols affected individuals, slashes deceased, and double horizontal lines consanguinity. *NADSYN1* variant details are provided underneath individuals.

To date, 11 individuals have been reported with CNDD caused by biallelic *NADSYN1* variants (7, 11–13), the majority of whom have not survived past 3 months of age due to the severity of their malformations. Here, 12 more individuals from 10 unrelated families were identified, either through personal communications or GeneMatcher (14), presenting with various congenital malformations consistent with CNDD and biallelic *NADSYN1* variation (Figure 1). No individual was identified as part of a cohort enriched for specific congenital anomalies.

#### Clinical features of individuals carrying biallelic NADSYN1 variants are consistent with CNDD.

All 12 individuals described herein had biparental inheritance of *NADSYN1* variants in conjunction with various structural congenital anomalies; heterozygous carrier parents were unaffected. Most of the individuals were surviving (8 of 12); 1 died in utero (F1.II.1), 1 died 13 days after birth (F6.II.2), and the remaining ones were terminated during pregnancy owing to significant malformation; 2 families reported additional miscarriages with unknown *NADSYN1* variant status (Figure 1 and Table 1). Overall, observed malformations were heterogeneous but consistent with those previously identified in CNDD cases (9). Congenital abnormalities mostly affected the vertebrae (10 of 12), heart (9 of 12), and limbs (8 of 12) while less frequently affecting the kidneys (3 of 12). Additional frequent findings across individuals included the occurrence of mild facial dysmorphism (7 of 12) and craniofacial (6 of 12), growth (5 of 12), neurodevelopmental (4 of 12), and central nervous system (3 of 12) abnormalities (Table 1 and Supplemental Table 1). There was phenotypic variability within each affected system, with heart defects, for example, ranging from relatively mild abnormalities of the aortic arch to life-threatening hypoplastic left heart (Supplemental Table 1). This variability was further exemplified within family 8 and between families 6 and 7, who share the same homozygous *NADSYN1* genotype. The most extreme variability was observed within family 9, in which only one (F9.II.4) of two siblings, both homozygous for a *NADSYN1* frameshift variant, exhibited malformations. Both siblings, however, experienced previously unseen life-threatening episodes of pellagra-like dermatitis, a characteristic postnatal consequence of NAD deficiency (15) that has not been reported previously in NADSYN1 deficiency. Additional previously unreported congenital phenotypes such as a reduced lobe count in the lungs (F8.II.2) were also observed (Supplemental Table 1). Detailed descriptions of individuals with biallelic *NADSYN1* variants are presented in the Supplemental Results.

#### Identified NADSYN1 variants have predicted deleterious impact on substrate binding and protein function in silico.

**Figure 2:**
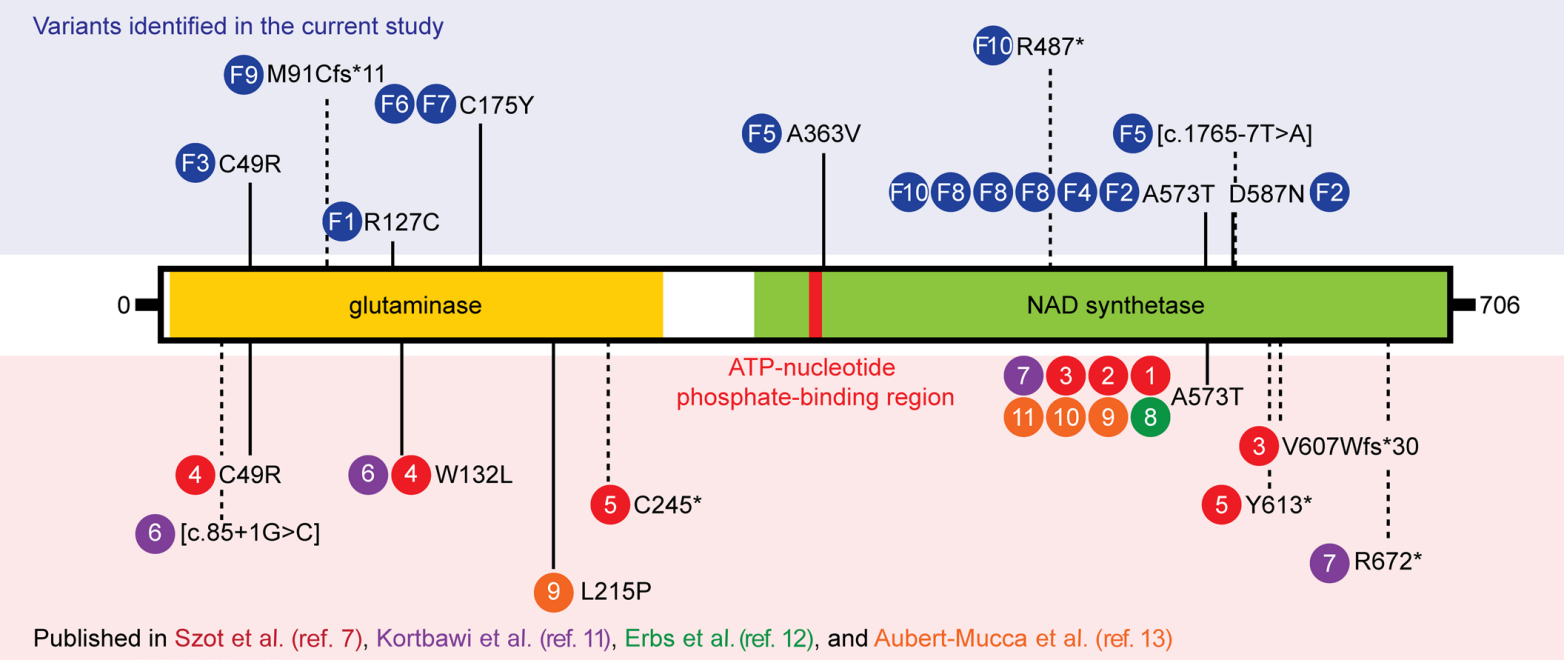
Positions of previously and newly identified NADSYN1 variants identified relative to functional protein domains. Blue circles indicate variants identified in the current study and respective family (see Figure 1). Other colored circles with numerals indicate study origin of identified variants and the chronology of their identification, respectively. Previously published variants have been reported in refs. 7, 11–13. Solid and dashed lines distinguish missense from presumed loss-of-function variants due to altered reading frame and protein truncation, respectively.

All *NADSYN1* variants were identified via exome or genome sequencing (Supplemental Methods) and in the absence of any competing predicted disease-causal variants. *NADSYN1* c.145T>C p.C49R and c.1717G>A p.A573T have been reported in unrelated CNDD patients (7, 11, 12), with the latter variant identified in 50% of probands in this cohort (Figures 1 and 2) despite their heterogeneous ancestries and lack of consanguinity. Variant c.1765-7T>A, found in patient F5.II.1, was also reported in a patient with unspecified “malformation syndrome,” and causes 3 abnormal splicing events, each resulting in protein truncation (16). These protein truncations, including those caused by novel frameshift and truncating variants c.271del p.M91Cfs*11 and c.1459C>T p.R487* in families 9 and 10, respectively, are more N-terminal than known pathogenic protein-truncating variants (Figure 2) and are similarly predicted to lead to loss of function. All variants scored greater than 20 with respect to CADD-PHRED (v1.6) (17), placing them in the top 1% of deleterious variants, except for splice variant c.1765-7T>A (16.94). Similarly, all variants scored greater than 0.025 and were classified as possibly damaging with respect to M-CAP (18) except for c.1088C>T p.A363V with a score of 0.017 (Table 2). All variants are rare with respect to individuals lacking pediatric disease in the gnomAD database (v4.0.0) with an allele frequency less than 0.1%, and none, except c.1765-7T>A and c.1717G>A p.A573T, were observed in the homozygous state in gnomAD (19).

All *NADSYN1* missense variants occur in the N-terminal glutaminase or the C-terminal NAD synthetase domain (Figure 2). To first assess variant impact on protein structure and function, we computed changes in binding affinities to cofactor adenosine triphosphate (ATP) and NADSYN1 substrates nicotinic acid adenine dinucleotide (NaAD) and glutamine (Supplemental Methods). Moderate to severe loss of ATP and NaAD binding affinities were seen for synthetase variants, while only mild losses in glutamine affinity were observed for glutaminase domain variants (Supplemental Table 2 and Supplemental Figure 2). Given that NaAD binding at the active site is necessary for enhancement of both glutaminase activity and NAD synthesis (20), these combined observations suggest that while all variants are predicted to disrupt protein function, those that affect NaAD binding should cause the greatest protein dysfunction.

#### NADSYN1 variants affect NAD synthetase activity in vitro.

Human NADSYN1 protein is equally capable of utilizing environmental glutamine or ammonia as an amide source for NaAD amidation when either co-substrate is provided in excess (20). Therefore, we compared both glutamine- and ammonia-dependent NAD synthetase activities of purified wild-type NADSYN1 protein with those of the missense variants identified in these families, except for p.C49R because of its previously reported instability in vitro (7).

**Figure 3:**
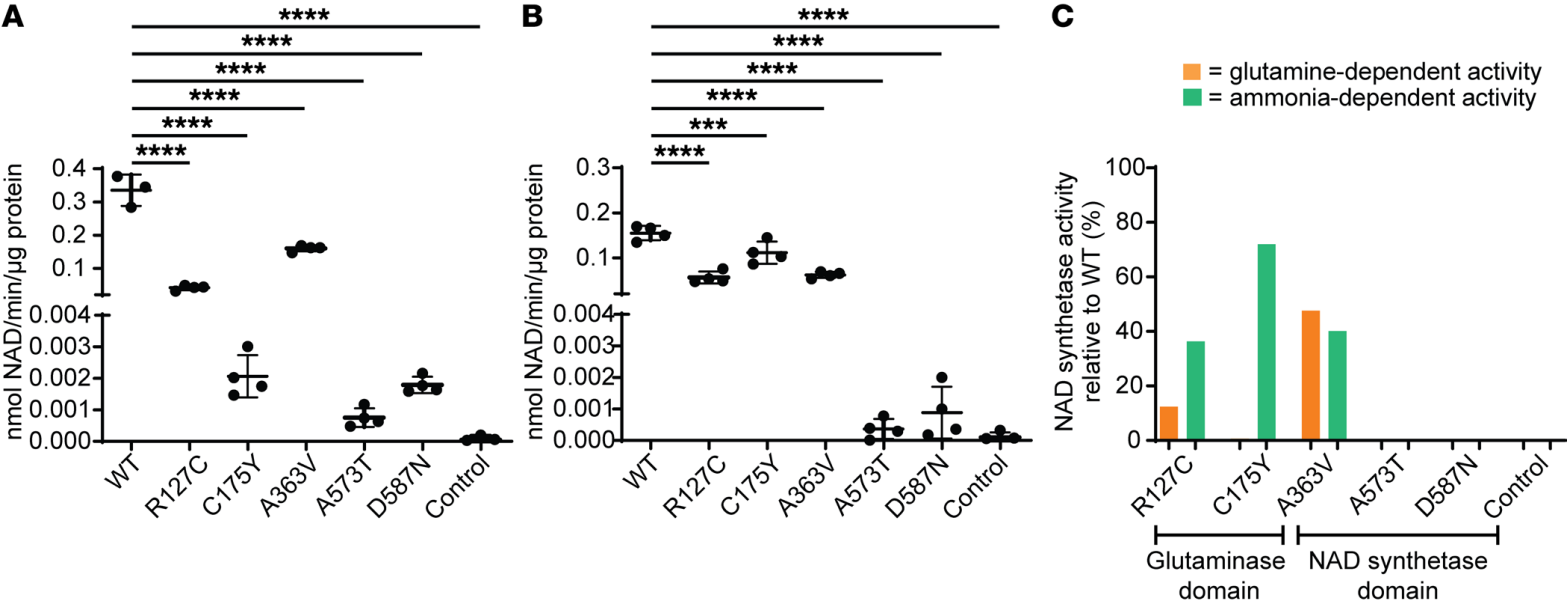
Functional assessment of NADSYN1-variant proteins corresponding to gene variants identified in affected individuals. (**A**) NADSYN1 activity of purified variant proteins compared with wild-type NADSYN1 protein in the presence of glutamine as substrate. One-way ANOVA with Dunnett’s post hoc test. (**B**) NADSYN1 activity of purified variant proteins compared with wild-type NADSYN1 protein in the presence of ammonia as substrate. One-way ANOVA with Dunnett’s post hoc test. (**C**) Average NADSYN1 activity of variant protein relative to wild-type protein activity with respect to positions of variant sites in functional protein domains. ***P < 0.001, ****P < 0.0001; *n* = 4 experiments. WT, wild-type NADSYN1 protein; Control, negative control (untransfected cell lysate).

Wild-type NADSYN1 and variants p.R127C, p.C175Y, p.A363V, p.D587N, and p.A573T were detected at similar levels in transfected COS-7 cells (Supplemental Figure 3A). NADSYN1 wild-type and variant proteins were purified (Supplemental Figure 3B) and NAD synthetase activity assessed in either excess glutamine or ammonia. In the presence of excess glutamine, all variants exhibited significantly reduced capacity to synthesize NAD compared with wild-type protein, with p.D587N, p.A573T, and p.C175Y exhibiting near-complete loss of function (Figure 3A). When provided with excess ammonia, all variants exhibited significantly reduced activity, with p.D587N and p.A573T lacking activity (Figure 3B). The p.A363V variant, positioned within the ATP binding site, showed consistently reduced activity using either substrate. These domain-specific perturbations are consistent with our in silico predictions and reinforce that all identified variants significantly disrupt NADSYN1-dependent NAD synthesis, with variants in the C-terminus consistently lacking activity, irrespective of substrate (Figure 3C).

### Biallelic *NADSYN1* loss-of-function variants impact the NAD metabolome of humans

Prior studies report changes to metabolite levels in individuals with pathogenic *KYNU*- or *HAAO*-inactivating variants (8, 21), but to date, no metabolic assessment of individuals with NADSYN1-dependent CNDD has been performed. To understand the extent of NAD pathway perturbation caused by biallelic *NADSYN1* variants, we collected whole blood and plasma from 3 individuals (F3.II.2 and F5.II.1 were fasting; F7.II.3 was not fasting) and from their fasting heterozygous carrier parents. Levels of NAD and 25 associated metabolites, defined as the NAD metabolome, were quantified by an ultra-high-performance liquid chromatography–tandem mass spectrometry assay (22).

**Figure 4:**
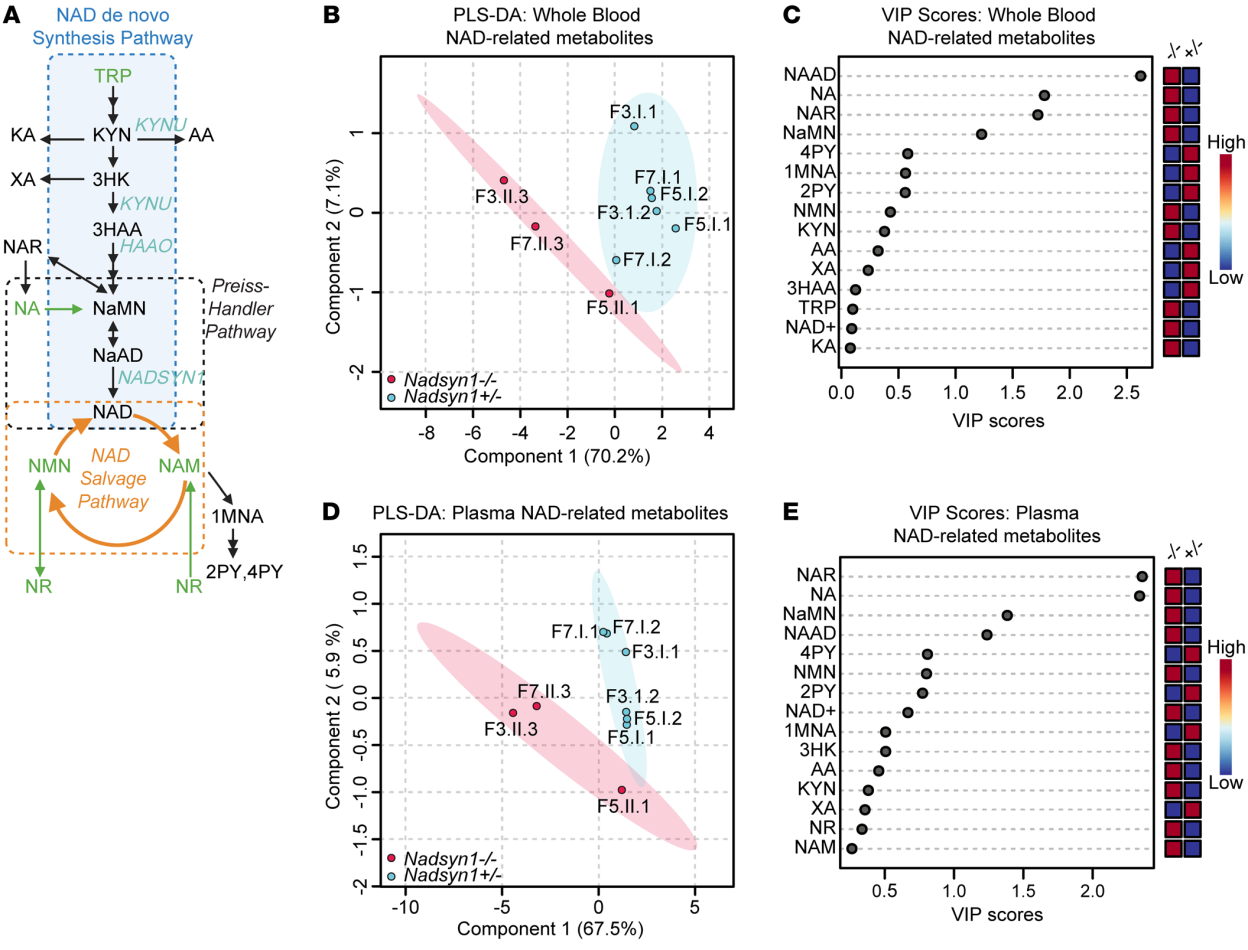
Whole-blood and plasma NAD metabolomic profiles in individuals with biallelic *NADSYN1* variants and their heterozygous parents. (**A**) Simplified NAD biosynthesis pathway; genes in which biallelic pathogenic variants cause CNDD (cyan), and B__3__ vitamers (green). (**B** and **D**) Partial least squares–discriminant analysis (PLS-DA) 2-dimensional score plots of proband and parental whole blood (**B**) and plasma (**D**). Affected individuals (red) and parents (cyan) are denoted by their pedigree IDs (see Figure 1). (**C** and **E**) Respective variable importance in projection (VIP) plots. The most discriminating metabolites are shown in descending order of their VIP scores. *n* = 3 probands and their 6 parents, respectively. AA, anthranilic acid; 3HK, 3-hydroxykynurenine; KYN, kynurenine; 1MNA, 1-methylnicotinamide; NA, nicotinic acid; NaAD, nicotinic acid adenine dinucleotide; NAD^^+^^, nicotinamide adenine dinucleotide; NAM, nicotinamide; NaMN, nicotinic acid mononucleotide; NAR, nicotinic acid riboside; NMN, nicotinamide mononucleotide; NR, nicotinamide riboside; 2PY, *N*^^1^^-methyl-2-pyridone-5-carboxamide; 4PY, *N*^^1^^-methyl-4-pyridone-3-carboxamide; XA, xanthurenic acid. See Supplemental Figure 1 for an overview of the NAD synthesis pathways and associated metabolites. Metabolite concentration values are provided in Table 3 and Supplemental Table 3.

To identify significant differences in NAD-related metabolites (Figure 4A) between affected individuals and carrier parents, we performed partial least squares–discriminant analysis on both whole blood and plasma with variable importance in projection (VIP) scores computed to identify the most important metabolites for clustering. NAD metabolomes of all 3 affected individuals clustered together and were separate from those of all heterozygous parents, who formed a second cluster (Figure 4, B and D). Elevated Preiss-Handler metabolites NA, nicotinic acid riboside (NAR), nicotinic acid mononucleotide (NaMN), and NaAD most strongly distinguished affected individuals from parents, in whom these were barely or not detectable. Downstream of NADSYN1, affected individuals also exhibited decreased salvage pathway excretion products 1-methylnicotinamide (1MNA), *N*^^1^^-methyl-2-pyridone-5-carboxamide (2PY), and *N*^^1^^-methyl-4-pyridone-3-carboxamide (4PY) (Figure 4, C and E, and Table 3). These excretion products occur due to methylation of surplus NAM when enough is available for salvage to NAD (23). Therefore, while NAD levels were not consistently altered between affected individuals and parents, NAM conservation by minimizing production of excretion products in affected individuals indirectly reflects their diminished NAD availability. Tryptophan and intermediates of the NAD de novo synthesis pathway upstream of NaMN were relatively consistent among all tested individuals (Supplemental Table 3). Fasting plasma samples from F4.II.2 were assessed for 806 biochemicals nonspecific to the NAD metabolome by Baylor Genetics (Supplemental Table 4). Levels of NA were elevated, while 1MNA and 2PY were reduced, relative to control populations. Together, these data indicate that individuals with biallelic *NADSYN1* variants are identifiable by their accumulation of Preiss-Handler metabolites and minimization of excretion products in circulation.

#### NAM supplementation alters the NAD metabolome.

NAD levels in individual F7.II.3 were 1.7-fold lower than those of their parents and below those of both F3.II.3 and F5.II.1. Therefore, the potential for NAD replenishment in F7.II.3 was assessed via 2 supplemental dosages of NAM, first at 50 mg/d for 6 weeks followed by 100 mg/d for another 6 weeks. Whole blood and plasma were collected at the end of each dosage period (Table 3). Both dosages elevated whole-blood NAD^^+^^ to levels higher than those of parents, while NAM levels only increased with supplementation of 100 mg/d NAM. Corresponding with this increase in NAD, NAM supplementation elevated excretion product levels 2.2- to 4.3-fold over presupplementation values with a proportional further increase of about 4-fold when supplementation was doubled. This indicates that even 50 mg/d NAM was sufficient to restore NAD availability to within a normal range (24). In addition, levels of NA and NaMN increased 3.7- and 1.6-fold, respectively, in whole blood following supplementation with 100 mg/d NAM, whereas trends for other Preiss-Handler metabolites and those in plasma did not follow a consistent trend (Table 3).

We next addressed whether these elevated metabolite levels in the circulation were retained or excreted by assessing the NAD metabolome in urine samples. Samples were collected from individual F7.II.3 twice under nonfasting conditions, and finally during the supplementation with 50 mg/d NAM under fasting conditions alongside that of their parents. NA, NAR, and NaMN were highly abundant in F7.II.3’s urine, whereas these metabolites were absent in the paternal sample, and only NA and NAR were detected in the maternal sample at lower levels (Supplemental Table 5). After supplementation with 50 mg/d NAM, F7.II.3’s salvage pathway excretion products 1MNA, 2PY, and 4PY increased in concentration 6.3-fold, more than 3.6-fold, and 11.6-fold, respectively, compared with presupplementation values. Regardless of supplementation status, neither NaAD nor NAD could be detected in F7.II.3’s urine or parental urine. Together, these data indicate that NAM supplementation effectively replenished available NAD levels in this affected individual. Furthermore, elevated Preiss-Handler metabolites that cannot effectively be used for NAD synthesis and salvage pathway waste products are excreted in the urine.

### Loss of *Nadsyn1* disrupts embryogenesis in mice

To better understand the consequences of NADSYN1 inactivation, we generated a null *Nadsyn1* allele in mice (Supplemental Figure 4). We confirmed that null mice (*Nadsyn1^–/–^*) had no NADSYN1 activity by measuring enzymatic function in the liver, where NADSYN1 is most active (4) (Supplemental Figure 5).

#### Nadsyn1^^–/–^^ mouse embryos develop NAD-dependent malformations when maternal dietary NAD precursors are limited during gestation.

Genes involved in NAD synthesis as well as the maternal diet affect gestational NAD levels in mice, and under conditions that cause NAD deficiency, mice either cannot support a pregnancy or generate embryos with malformations (8, 10). Conversely, on diets plentiful in NA, pregnancies are unaffected despite maternal or embryonic homozygous loss of function of *Kynu* (*Kynu^–/–^*) or *Haao* (*Haao^–/–^*), genes of the NAD de novo synthesis pathway, because NA bypasses their metabolic block via the Preiss-Handler pathway (Supplemental Figure 1). Correspondingly, when *Nadsyn1^–/–^* female mice were mated with heterozygous (*Nadsyn1^+/–^*) males and provided with a Breeder Diet with abundant NAD precursors (Supplemental Table 6) during pregnancy, all generated embryos were present according to Mendelian ratios and morphologically normal at E17.5–E18.5 (data not shown).

As established for *Haao^–/–^* mice (10), we next pretreated female mice with an NAD precursor–rich Standard Diet (Supplemental Table 6) to equalize maternal NAD levels prior to pregnancy. *Nadsyn1^+/–^* intercross then recapitulated NAD-dependent defects in E18.5 embryos (Supplemental Table 7) only when provided with a diet with restricted NAD precursor content (Limited Diet; Supplemental Table 6) during pregnancy. Malformations affected the heart, kidneys, vertebrae, tail, palate, eyes, abdominal wall, and neural tube, consistent with previous mouse models of CNDD (8, 10) (Supplemental Figure 6). Previously unseen malformations of the lungs were also observed (Supplemental Table 7). In contrast to *Nadsyn1^+/–^* mice, pregnancy outcomes could not initially be assessed in *Nadsyn1^–/–^* mice because of their progressive weight loss when maintained on the pretreatment Standard Diet (Supplemental Figure 7A), presumably due to their inability to use either tryptophan or NA in the diet as NAD precursors. We therefore substituted NA in the pretreatment diet with NAM (Sufficient Diet; Supplemental Table 6), predicting that this would bypass the metabolic block caused by complete loss of *Nadsyn1*. Correspondingly, on this Sufficient Diet, mice of all *Nadsyn1* genotypes equally gained weight (Supplemental Figure 7B and Supplemental Figure 8). To enable future comparisons in pregnancy outcomes between *Nadsyn1^+/–^* and *Nadsyn1^–/–^* mice, all subsequent experimental mice were pretreated with the Sufficient Diet.

Embryos generated by *Nadsyn1^+/–^* intercross on the Sufficient Diet were all alive and largely normal (97%) at E17.5, whereas 39% of all offspring generated on the Limited Diet either were malformed or died during development (Table 4, Supplemental Figures 9 and 10, and Supplemental Figure 11, A and B). Dead embryos (29% of total) could not be genotyped, but there were fewer than expected *Nadsyn1^–/–^* live embryos (Supplemental Figure 11C), indicating that *Nadsyn1^–/–^* embryos were predominantly dying. Furthermore, over half of *Nadsyn1^–/–^* embryos had isolated or multiple malformations, while few defects were identified across wild-type (*Nadsyn1^+/+^*) or *Nadsyn1^+/–^* littermates (Supplemental Figure 9). Affected *Nadsyn1^–/–^* embryos most frequently exhibited malformations of the kidneys, eyes, and lungs (Supplemental Figure 11B and Supplemental Table 8).

Given our previous observation that Preiss-Handler pathway metabolites accumulate in humans with biallelic loss-of-function *NADSYN1* variants (Figure 4), we hypothesized that the Limited Diet, owing to its predominant tryptophan-based composition and minimal NAM content relative to the Sufficient Diet (Supplemental Table 6), would not supply *Nadsyn1^–/–^* female mice with sufficient NAD precursors to support a pregnancy. Therefore, we compared embryo outcomes of *Nadsyn1^–/–^* mothers on the Limited Diet versus the Sufficient Diet. On the Sufficient Diet, 21% of offspring were dead or malformed (Table 4 and Supplemental Figure 9). Of 24 live embryos, only 2 (8%) were malformed. On the Limited Diet, all embryos were dead (Table 4 and Supplemental Figure 10). These data collectively show that *Nadsyn1^–/–^* mouse embryos recapitulate human CNDD phenotypes when maternal NAD precursors are limited.

#### Amidated (NMN, NAM), but not deamidated (NaMN, NA), B__3__ vitamers can bolster maternal NAD availability in Nadsyn1^^–/–^^ mice.

NADSYN1 amidates NaAD to generate NAD as the common final step of both NAD de novo and Preiss-Handler pathways. As *Nadsyn1^–/–^* mice cannot use tryptophan or NA to generate NAD, but could sustain pregnancies on the NAM-rich Sufficient Diet, we hypothesized that amidated NAD precursors such as NMN and NAM would replenish NAD levels. We therefore assessed liver NAD stores and the plasma NAD metabolome of female mice on the Limited Diet with and without equimolar supplementation of amidated (NMN) or deamidated (NA, NaMN) NAD precursors.

**Figure 5:**
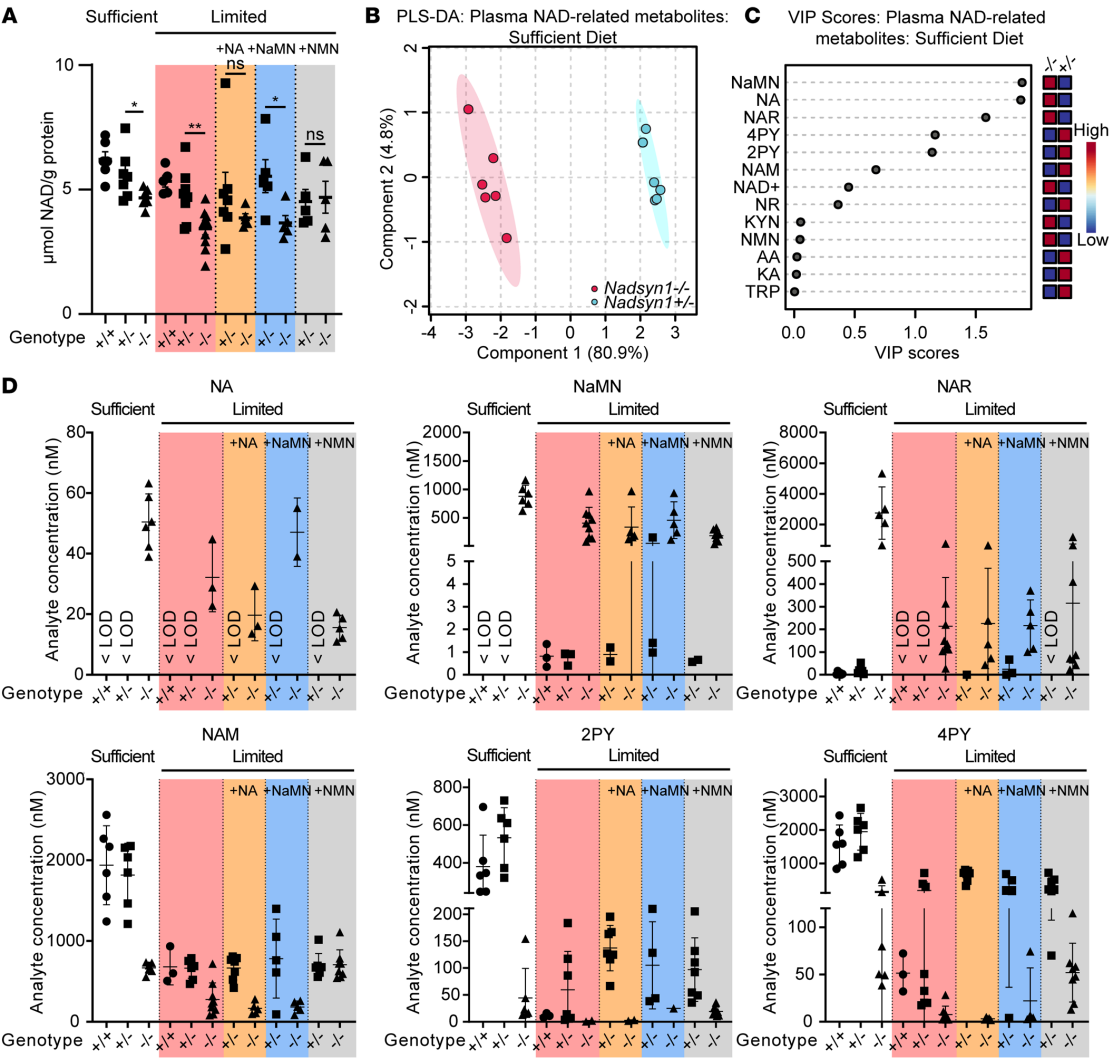
Liver NAD and NAD-related metabolites in plasma of female mice of different *Nadsyn1* genotypes under various dietary conditions. All mice were pretreated with the Sufficient Diet for more than 21 days and then provided with the indicated diets for 17 days, after which they were dissected and liver tissue and plasma collected. (**A**) Liver total NAD. Statistical comparisons represent within-diet 2-tailed Student’s *t* test between *Nadsyn1^+/–^* and *Nadsyn1^–/–^* mice. *P < 0.05, **P < 0.01; *n* = 5–10 mice per condition. For numerical values, see Supplemental Table 9. (**B** and **C**) Partial least squares–discriminant analysis (PLS-DA) 2-dimensional score plots (**B**) and corresponding variable importance in projection (VIP) plots (**C**) comparing plasma metabolite levels between female *Nadsyn1^+/–^* and *Nadsyn1^–/–^* mice fed the Sufficient Diet; *n* = 6 mice per condition. (**D**) NAD-related metabolites with the highest VIP scores in mouse plasma. *Nadsyn1* genotypes are shown below each graph and dietary conditions on top of graphs. Bars indicate mean ± standard deviation; *n* = 5–10 mice per condition. Values for the other measured NAD-related metabolites are summarized in Supplemental Figure 12. “< LOD” indicates below the limit of detection. See Supplemental Figure 1 for an overview of the NAD synthesis pathways and associated metabolites.

Consistent with *Nadsyn1^–/–^* mice on the NA-rich Standard Diet (Supplemental Figure 7A), *Nadsyn1^–/–^* mice progressively lost weight when provided with the Limited Diet alone and when supplemented with NA or NaMN (Supplemental Figure 8). By contrast, this weight loss was abated when NMN was provided, as with the NAM-rich Sufficient Diet during mouse pretreatment. No weight loss was observed in *Nadsyn1^+/–^* mice on any test diet. Total NAD content in liver of these treated mice significantly decreased in proportion to loss of *Nadsyn1* alleles, reflecting the role of *NADSYN1* in liver NAD synthesis (3, 4), whether NAD precursors were in excess (Sufficient Diet) or restricted (Limited Diet). NAD levels were lowest in *Nadsyn1^–/–^* livers of mice fed the Limited Diet, and this was not improved by supplementation with NA or NaMN, whereas supplementation with NMN significantly raised NAD levels (Figure 5A and Supplemental Table 9). This reinforced that NAD replenishment via the salvage pathway was independent of functional NADSYN1 and suggested that sufficient NAD levels in the liver were required to maintain weight (Supplemental Figure 8).

We next quantified the plasma NAD metabolome of these female mice. In *Nadsyn1^–/–^* mice on the Sufficient Diet, Preiss-Handler metabolites accumulated and excretion products decreased (Figure 5, B–D), as seen in humans with biallelic *NADSYN1* variants. On the other diets, Preiss-Handler metabolites were also consistently elevated in *Nadsyn1^–/–^* mice. On diets that caused weight loss in *Nadsyn1^–/–^* mice, excretion products were barely detectable (Figure 5D and Supplemental Figure 8) and NAM levels only reached 24%–42% of those in *Nadsyn1^+/–^* mice under diet-matched conditions, collectively indicating severely diminished NAD bioavailability. By contrast, when NMN was provided to *Nadsyn1^–/–^* mice, NAM levels equaled those of *Nadsyn1^+/–^* mice with a corresponding increase in excretion products, liver NAD (Figure 5A), and weight gain (Supplemental Figure 8). No NAD metabolomic differences were observed in *Nadsyn1^+/–^* plasma on any diet besides the Sufficient Diet, on which surplus NAD precursors elevated levels of NAM and excretion products. Circulatory levels of NAD, NMN, and NR were in the low nanomolar range, consistent with previous reports (22), and too low under all conditions to observe significant changes (Supplemental Figure 12). Finally, tryptophan and intermediates of the NAD de novo synthesis pathway were similar or only altered to a lesser extent between treatment conditions or *Nadsyn1* genotypes (Supplemental Figure 12 and Supplemental Table 10).

Together, these data show that complete loss of *Nadsyn1* manifests a characteristic plasma metabolomic profile in mice consisting of Preiss-Handler pathway metabolite accumulation coinciding with minimal excretion product formation, consistent with *NADSYN1* CNDD individuals. Furthermore, on diets lacking amidated NAD precursors, *Nadsyn1^–/–^* mice become NAD deficient and lose weight, which is preventable by amidated NAD precursor (NMN, NAM) supplementation.

#### NADSYN1-dependent embryo loss and malformation in mice are preventable by dietary amidated NAD precursor supplementation during pregnancy.

Given that the type of NAD precursor supplementation significantly affected the health and NAD status of female mice, we next addressed the effects of these diets during pregnancy. As expected, intercross of female *Nadsyn1^–/–^* with male *Nadsyn1^+/–^* mice on the Limited Diet supplemented with NA or NaMN resulted in pregnancy loss with no viable embryos generated (Table 4). By contrast, alive and normal embryos were generated on the Sufficient Diet (Supplemental Figure 13A). Similarly, supplementation of the Limited Diet with NMN enabled the generation of alive and normal embryos in 5 of 6 litters (Table 4). In the remaining litter, all embryos exhibited one or more malformations, irrespective of *Nadsyn1* genotype, suggesting that the mother did not consume enough NAD precursors during gestation (Supplemental Figures 9 and 10 and Supplemental Figure 13B). For *Nadsyn1^+/–^* females, supplementing the Limited Diet with either NA, NaMN, or NMN significantly reduced the number of affected embryos from 39% to 2%–10% (Table 4). There were overall fewer embryo deaths, from 29% with Limited Diet alone to 0%–5% with supplementation with either NAD precursor, and the overall incidence of malformed embryos decreased from 14% to 0%–5% (Table 4 and Supplemental Figures 9 and 10). The few malformations observed under these conditions did not show a significant difference in incidence between *Nadsyn1* genotypes (Supplemental Figure 14). Taken together, these data show that all B__3__ vitamers can ameliorate adverse pregnancy outcomes in mice when the mother has at least one functional copy of *Nadsyn1*. By extension, *Nadsyn1^–/–^* mothers only benefit from supplementation with NMN (Limited Diet + NMN) or NAM (Sufficient Diet), indicating that maternal NAD bioavailability determines pregnancy outcome independent of embryonic *Nadsyn1* genotype.

We then addressed whether pregnancy itself, owing to the increased metabolic demand from developing embryos (9), affected maternal liver NAD stores and the circulatory NAD metabolome at a critical period of embryonic organogenesis, E11.5 (25). This analysis was performed on *Nadsyn1^+/–^* and *Nadsyn1^–/–^* mothers provided with NMN, as this supplement showed consistent benefit during pregnancy; these mothers were compared with *Nadsyn1^+/–^* mothers without supplementation or provided with the Sufficient Diet. Overall, the NAD metabolome of pregnant mice was similar to that of non-pregnant female mice in all matched conditions (Figure 5D, Supplemental Figure 15A, and Supplemental Table 11), though liver NAD appeared consistently lower (Supplemental Figure 15B). This suggested that mouse dietary habits did not significantly change as a result of pregnancy but that pregnancy itself altered liver NAD stores, as previously reported (10). All E11.5 embryos generated from *Nadsyn1^+/–^* mothers were without external malformation (Supplemental Figure 16A) and consistent with trends at E17.5. Correspondingly, total NAD in embryos was variable but consistently elevated with maternal NMN supplementation (Supplemental Figure 15C and Supplemental Table 12), suggesting a direct benefit to embryonic NAD from increased maternal NAD bioavailability. Finally, E11.5 *Nadsyn1^–/–^* embryos generated from *Nadsyn1^–/–^* mothers exhibited NAD levels significantly lower than those in *Nadsyn1^+/–^* littermates (Supplemental Figure 15D) — levels previously reported to be at the threshold of sufficiency for normal development (10). As such, embryos were observed in expected Mendelian ratios and without external malformation (Supplemental Figure 16B) as seen by E17.5 (Table 4). *Nadsyn1^–/–^* mothers on Limited Diet + NMN exhibited adequate levels of plasma NAM to support embryonic development (Supplemental Figure 15A), similar to those of pregnant *Nadsyn1^+/–^* mothers. However, their near-zero levels of plasma excretion products suggested that maternal NAD levels were only just sufficient to sustain a pregnancy.

In summary, maternal diet–derived NAD precursors primarily determine the development of healthy embryos. However, when maternal NAD precursors are limited, embryonic NAD de novo synthesis contributes to the overall NAD level, accounting for differences in NAD level and potential developmental outcome differences between *Nadsyn1^+/–^* and *Nadsyn1^–/–^* embryos.

## Discussion

In this study, we report 13 new individuals across 10 families with biallelic loss-of-function variants in *NADSYN1*. All previously reported individuals with NADSYN1-dependent CNDD have biallelic loss-of-function variants that exhibit less than 1% glutamine-dependent NAD synthetase activity relative to wild-type protein (9). Similarly, all our tested variants exhibited significantly reduced glutamine-dependent activity but, interestingly, retained more than 36% ammonia-dependent activity when located in the glutaminase domain. This domain-specific perturbation is consistent with studies in NADSYN1 orthologs (26–28) but particularly clear for glutaminase mutant p.C175Y, which retains near-wild-type activity when provided with ammonia as an amide source (27, 29). Of the 4 novel missense variants identified in our cohort, p.C175Y also represents the first reported pathogenic human variant, to our knowledge, of this nucleophilic cysteine, which is centrally conserved across the nitrilase superfamily (30). NaAD amidation is ultimately dependent on a functional synthetase domain. Accordingly, variants found in our study that occur in the synthetase domain active site were most disruptive to NAD synthesis. In general, in silico predictions of domain-specific impacts to protein structure and function were concordant with assessed activity in vitro (Supplemental Results). Nevertheless, deleteriousness of respective *NADSYN1* variants did not directly correlate with severity of phenotype in our affected individuals and those reported previously (7, 11–13), underscoring the possible impact of environmental modifiers in CNDD causation.

The CNDD cases reported here exhibit various combinations of abnormalities involving vertebral, cardiac, renal, or limb defects, reinforcing the defining clinical features of this disorder (9). In addition, all our cases exhibited various additional congenital or developmental anomalies not previously reported in CNDD cohorts. One new abnormality in particular, lung aplasia in patient F8.II.2, is of note because it was reproduced in *Nadsyn1^–/–^* mouse embryos, further confirming its association with CNDD and suggesting that lung morphology be assessed in humans with a suspected CNDD diagnosis. Of the surviving individuals with biallelic *NADSYN1* variants, 4 of 8 (50%) presented with some form of neurodevelopmental anomaly, consistent with incidence in previously identified surviving CNDD patients (10 of 18, 56%; ages ranging from 0.5 to 30 years old) (7, 8, 11–13, 21, 31, 32). In our cohort, neurodevelopmental anomalies were diagnosed consistently in individuals with facial dysmorphism (4 of 4, 100%). This trend is similarly observed in published surviving CNDD cases (8 of 10, 80%), suggesting that the presence of facial dysmorphism is predictive of neurodevelopmental anomalies. After internal comparisons of facial dysmorphism between affected individuals of our cohort and with the limited available images of published CNDD cases (8, 12, 13, 31), we conclude that there is no specific facial gestalt defining CNDD. Rather, identification of mild facial dysmorphism may guide differential diagnoses of CNDD in unresolved cases of vertebral, cardiac, renal, and limb malformations, such as VACTERL association (33), especially for individuals presenting with disproportionate growth or neurodevelopmental disorders. Given the consanguinity in 4 of 10 families, it is possible that uncharacteristic features may be explained by additional, unascertained, genetic variants.

Our affected individuals exhibited consistent accumulation of the NADSYN1 substrate NaAD and upstream metabolites extending to those of the Preiss-Handler pathway in plasma and whole blood, suggesting that these might be good biomarkers for NADSYN1-dependent CNDD. NaAD is not detectable in healthy human blood and thus has only been reported as a biomarker of the NAD-boosting supplement NR, when systemic NAD levels are already sufficiently high (34). In addition to NaAD, NaMN was highly elevated in all proband plasma and urine samples, while it was undetectable in parental samples, consistent with reports for healthy individuals (35). NaMN accumulation is likely secondary to that of NaAD, as NaAD was not readily detectable in the plasma or urine, and their enzymatic interconversion by NMNAT1–NMNAT3 is reversible (36). Finally, the extreme elevation in levels of NAR in proband urine, plasma, and whole blood is a likely consequence of 5′-nucleotidases CN-II and CN-III, which may dephosphorylate NaMN to NAR only at the supraphysiological levels caused by NADSYN1 dysfunction (37). A similar trend, although to a lesser extent than with NAR, was seen with NA. These overall trends were observed in all tested individuals with biallelic *NADSYN1* variants of varying age, appeared in proportion to the in vitro deleteriousness of their respective NADSYN1 variants, and were reproduced in *Nadsyn1^–/–^* mice irrespective of diet. Given that the individuals from whom we could obtain NAD metabolome data have comparably less deleterious variants relative to individuals with NAD synthetase domain or protein-truncating loss-of-function variants, the latter might experience even greater accumulation of these metabolites.

Previous assessment of individuals with CNDD due to biallelic variants in *KYNU* or *HAAO* revealed decreased plasma NAD relative to heterozygous variant carriers (8), whereas this was not observed in our *NADSYN1* cohort. This inconsistency may first be attributable to differences in dietary intake, additional non-genetic factors, or differences in sample handling and processing. This may alternatively be due to differences in the degree of deleteriousness of specific variants. Assessed individuals have biallelic protein-truncating variants in *KYNU* or *HAAO* resulting in complete loss of function (8), whereas the *NADSYN1* variants in our 3 tested individuals have residual enzymatic activity (Supplemental Results), and therefore residual NAD de novo synthesis and Preiss-Handler activity may contribute to NAD replenishment. Plasma levels of NAD are in the low nanomolar range, even in healthy individuals (38), whereas the 2 main NAD precursors in circulation are tryptophan and NAM (3). Therefore, circulatory NAD is less reflective of systemic NAD availability than changes in NAM. Cellular NAD levels are efficiently maintained by NAMPT-mediated recycling of NAM via the salvage pathway, while excess NAM is irreversibly converted to 1MNA and its derivatives 2PY and 4PY in order to prevent feedback inhibition of NAD-consuming enzymes (39). Conversely, lowered plasma 2PY and 4PY levels, as seen in our individuals with biallelic *NADSYN1* variants compared with their parents, indicate that the probands’ metabolism was conserving NAM as a consequence of impaired NAD replenishment via other pathways besides NAM salvage. Concordantly, our *Nadsyn1^–/–^* mice exhibited near-zero plasma levels of salvage pathway excretion products, as well as lowered NAM, while they were NAD deficient. This metabolic signature correlated with weight loss, decreased liver NAD, and an inability to generate viable embryos due to limited NAD precursor availability from the diet. Weight loss has previously been observed in NAD-deficient mice (23, 40), causing a reduction of body fat and fatty liver deposits, and an inability to accumulate white adipose tissue. Unsurprisingly, these conditions were insufficient to meet the additional metabolic demands of a growing embryo. Consistent with this model, a *Nadsyn1*-knockout mouse line independently generated by the International Mouse Phenotyping Consortium (41) exhibited complete female infertility when maintained on a diet with tryptophan and NA as sole NAD precursors.

In agreement with other mouse models with knockout of *Kmo* (42), *Kynu*, *Haao* (8), or *Qprt* (23), no congenital anomalies in *Nadsyn1^–/–^* offspring were observed when dietary NAD precursors were abundant. It follows that, irrespective of which gene is mutated in the NAD de novo synthesis pathway, pregnancy success is primarily determined by the bioavailability of maternal NAD precursors. When NAM is abundant, sufficient embryonic NAD for normal development can be derived via the salvage pathway. Given the limited bioavailability of other salvage pathway metabolites, it is therefore the inadequate provision of NAM from mother to fetus that drives defect formation in *Nadsyn1^–/–^* embryos. Under the same conditions, *Nadsyn1^+/–^* embryos are less susceptible to CNDD owing to their ability to perform NAD de novo synthesis from the tryptophan provided in circulation. Additional studies will be required to determine how different NAD precursors integrate into NAD-deficient developing organisms and ameliorate disrupted NAD-dependent processes during embryogenesis (9).

Human trials have shown therapeutic benefit from NAM, NR, and more recently NMN (43) supplementation in elevating systemic NAD levels. The precise mechanisms by which these precursors bolster NAD levels and their tissue specificities, however, are still being explored. Our pregnant *Nadsyn1^–/–^* mice show that the salvage pathway metabolites NMN and NAM support normal embryonic development regardless of genetic disruption to maternal NAD de novo synthesis or Preiss-Handler pathway. By contrast, in pregnant *Nadsyn1^+/–^* mice, all B__3__ vitamers (NA, NAM, NMN, NR) improve the outcomes of *Nadsyn1^–/–^* embryos, and in humans this could be similarly effective in preventing a fetal CNDD phenotype. The dosage of NMN given to our mice to prevent adverse pregnancy outcomes equates to approximately 180% the daily recommended dietary intake (RDI) of vitamin B__3__ for pregnancy in humans (18 mg/d) (44), assuming certain rates of consumption in mice (10). However, an increased dose is likely required for more consistent benefit. The tolerable upper limit for NAM in non-pregnant adult humans is 900 mg/d (45), 28-fold above our tested dosage, although safe levels have not yet been established in pregnancy. Hepatotoxicity has been observed at dosages upward of 3,000 mg/d (46). Oral NMN supplementation has been shown to safely increase NAD levels in humans (47) and, given its conversion to NR for cellular uptake (48), is expected to be equally as capable as NR of elevating NAD levels. Concerning NR, human trials have assessed dosages of approximately 300 mg/d, with as little as 100 mg/d showing NAD-boosting effects (49), and up to 230 mg/d (~12-fold the RDI during pregnancy) proposed to be safe for pregnant or lactating women (50). Given that F5.I.2 supplemented her diet with 18 mg NAM per day during pregnancy, and F5.II.1 still developed NAD-dependent malformations, it is unlikely that this current dosage is sufficient to prevent NADSYN1-dependent CNDD in humans. Additional clinical trials are needed before accurate dosage recommendations required to prevent CNDD-dependent malformation can be made. It is also conceivable that post-birth phenotypes such as feeding difficulties, developmental delay, and short stature, seen in some of our cases as well as previous CNDD cohorts, may be prevented via supplementation strategies. A 1-month-old individual with CNDD caused by biallelic loss-of-function *HAAO* variants was reported to have improved in length and development after 3 months of NA supplementation (31). By contrast, after 14 months of NAM supplementation from 24 months of age, F7.II.3 had shown developmental progress to a level consistent with age, but remained microcephalic with mild improvement in growth parameters, and still exhibited mild emotional and behavioral dysregulation. Monitoring of progress in more supplemented individuals with CNDD, and for longer time frames, is necessary before it becomes clear whether these developmental phenotypes are indeed modifiable.

Only one individual with biallelic *NADSYN1* variants to date has been reported taking NAM supplementation, at 400 mg/d without any self-reported adverse outcomes, although it remains unclear whether this provided any gross benefit beyond superficial increase in blood NAD levels (12). Three individuals with NADSYN1-dependent CNDD in our cohort have similarly trialed NAM supplementation without reports of adverse effects: F7.II.3 at 100 mg/d for 6 weeks, who exhibited a 115% increase in blood NAD levels in comparison with unsupplemented conditions, elevated to levels similar to those of the aforementioned patient; F4.II.2 at 250 mg/d; and F9.II.4 at 200 mg/d for 8 weeks to prevent life-threatening NADSYN1-dependent pellagra-like dermatitis (NDPD; Supplemental Results). To our knowledge, this is the first report of NDPD and its prevention, likely occurring only in F9.II.3 and F9.II.4 due to their complete loss-of-function *NADSYN1* variants in combination with an additional environmental modifier such as inadequate dietary NAD precursors. These occurrences suggest that ongoing maintenance of NAD status in affected individuals via NAD precursor supplementation strategies may be warranted. It will be worthwhile to track progress in these affected individuals over time and, importantly, address whether supplementation may offset any age-dependent decline in NAD levels (51) or have a positive impact on long-term developmental and behavioral outcomes (8).

Finally, our data suggest that NADSYN1-dependent CNDD may be entirely preventable in humans, as one of two siblings with homozygous complete loss-of-function *NADSYN1* variants (F9.II.3 and F9.II.4, respectively) is devoid of any congenital malformation. This is further supported by the occurrence of an individual homozygous for A573T, the most commonly occurring pathogenic variant in CNDD and in our cohort; a homozygote for the loss-of-function splice variant c.1765-7T>A also reported in our cohort; and 2 homozygotes for the previously established pathogenic variant c.395G>C p.W132L (7) in gnomAD. These individuals were included in this database because they lacked severe pediatric disease (19), and, while it is not documented whether they experienced any post-birth anomalies common to NADSYN1-dependent CNDD, this reinforces the observation that NAD-dependent congenital malformation in humans, as in *Nadsyn1^–/–^* mouse embryos, may be prevented. This would also suggest that observation of a newfound variant’s being tolerated in the homozygous state in healthy-population databases like gnomAD should not be used to discredit its capacity to cause CNDD alone. Without strong negative selection pressure for recessive variants, it is likely that more NADSYN1-dependent CNDD cases will occur, and given enrichment for certain pathogenic variants in discrete populations, specific population groups may be more at risk. In gnomAD, W132L is observed disproportionately in admixed American populations with an allele frequency of 0.00259 versus 0.000129 overall; A573T is also enriched in European (non-Finnish) populations (allele frequency 0.00155 vs. 0.00118 overall). Haplotype analysis to confirm a founder variant status of the latter was not possible because of data sharing restrictions between carriers of this variant across clinical sites. Specific congenital malformations are heterogeneous in individuals with this biallelic variant and are equally variable in those with alternative biallelic *NADSYN1* variants (7, 9, 11–13), indicating that their identification was more likely biased by their considerable congenital malformation, and the availability of genetic testing, than by their *NADSYN1* genotype. Given the extent to which B__3__ vitamers in the maternal diet during pregnancy can prevent adverse development of *Nadsyn1^–/–^* mouse embryos, it is likely that many individuals with biallelic *NADSYN1* variants and milder congenital anomalies remain undiagnosed. For future CNDD cases, testing known blood biomarkers of *NADSYN1* disruption in patients, and similarly those of *KYNU* and *HAAO*, will better help identify those at risk of NAD deficiency–dependent development.

Taken together, our work presents a unique combination of CNDD phenotypic assessment, functional genetics, and NAD metabolome quantification in affected individuals and their carrier parents, with parallel replication in a CNDD mouse model. We have reaffirmed the importance of maternal and embryonic genotype as well as the gestational environment in the development of CNDD. Combined, our data demonstrate the necessity of appropriate B__3__ vitamer supplementation for individuals with biallelic loss-of-function variants in *NADSYN1*. Which B__3__ vitamer is used for supplementing heterozygous carriers, estimated to be 1 in 995 individuals for *NADSYN1* (9), is less essential. However, as these women are genetically predisposed to NAD deficiency, particularly when they experience myriad pathophysiological conditions that can perturb NAD homeostasis and NAD availability, it is vitally important that they have access to, and that they be encouraged to take, NAD-boosting supplements during pregnancy.

## Methods

Detailed methods are provided in Supplemental Methods.

### Statistics.

Statistical analyses, including 1-way ANOVA, 2-tailed Student’s *t* test, and 2-tailed 2×2 Fisher’s exact test, were performed with GraphPad Prism (v10.0.2). Significance was calculated, adjusting for multiple comparisons, at *P* less than 0.05. Bar graphs indicate mean and standard deviation.

### Study approval.

Written informed consent was received from all presented families prior to participation in this study. All animal experiments were performed in accordance with protocols approved by the Garvan Institute of Medical Research/St. Vincent’s Animal Experimentation Ethics Committee, Sydney, Australia, under approvals 18/27 and 21/18.

### Data availability.

The *NADSYN1* variants reported in this article are accessible in ClinVar with the submission number SUB13916655. Values for all data points are available in the Supporting Data Values file.

## Author contributions

SLD, GC, JOS, and HC conceptualized the project. JOS, HC, EMMAM, DZS, KI, S Portelli, VN, JMG, DA, K Bozon, GC, and DBA designed experiments, performed experiments, and/or analyzed data. KC, IMW, CVD, AL, EBW, WJ, LJ, MFB, LSP, TYT, JP, CAW, HW, IDA, BP, TAG, CP, S Prakash, BK, NJB, MFH, OH, SAL, JHM, NCJ, DC, and K Bouman identified patients and provided data and samples. JOS and HC curated data and performed the formal analysis. JOS, HC, K Bozon, and SLD provided overall intellectual input. JOS, HC, K Bozon, and SLD wrote the manuscript. All authors reviewed and edited the manuscript.

## Supplementary Material

Supplemental data

Supporting data values

## Figures and Tables

**Table 1:**
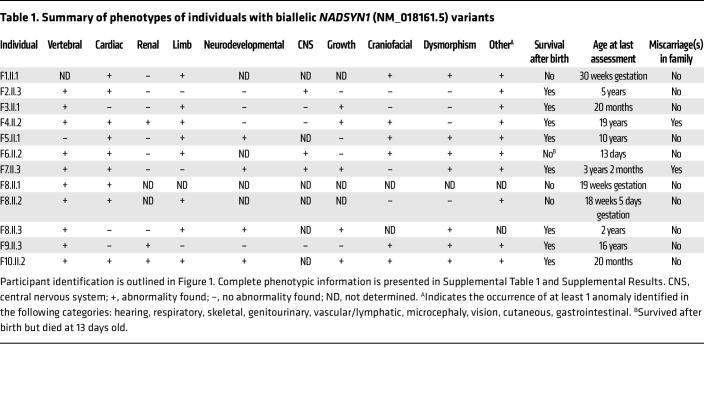
Summary of phenotypes of individuals with biallelic *NADSYN1* (NM_018161.5) variants

**Table 2:**
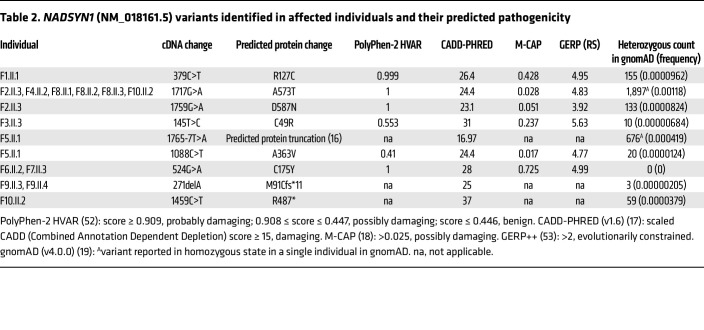
*NADSYN1* (NM_018161.5) variants identified in affected individuals and their predicted pathogenicity

**Table 3:**
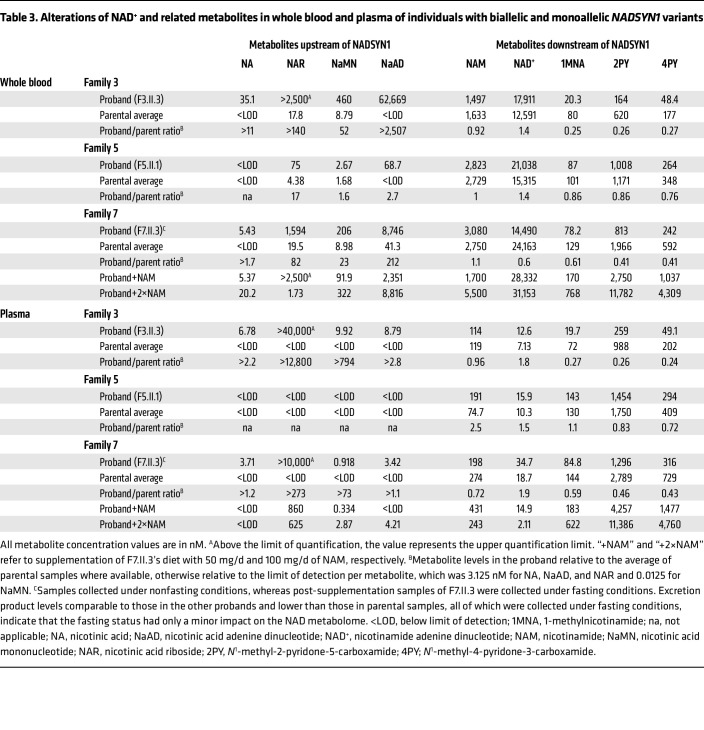
Alterations of NAD+ and related metabolites in whole blood and plasma of individuals with biallelic and monoallelic *NADSYN1* variants

**Table 4:**
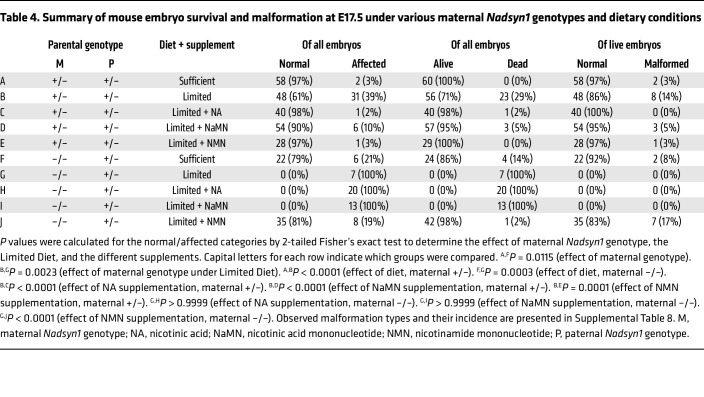
Summary of mouse embryo survival and malformation at E17.5 under various maternal *Nadsyn1* genotypes and dietary conditions

## Version 1 02/15/2024

Electronic publication
