# Supplementary material for: A metabolic signature for NADSYN1-dependent congenital NAD deficiency disorder
Source: J Clin Invest. 2024 Feb 15;134(4):e174824. doi: 10.1172/JCI174824 (PMC10866660; doi:10.1172/JCI174824)
Supplement: Supplemental data [file jci-134-174824-s287.pdf]

Supplemental Material

for

A metabolic signature for NADSYN1-dependent congenital NAD deficiency disorder

Justin O. Szot[1], Hartmut Cuny[1,2], Ella M. M. A. Martin[1], Delicia Z. Sheng[1], Kavitha Iyer[1], Stephanie Portelli[3,4], Vivien Nguyen[1], Jessica M. Gereis[1], Dimuthu Alankarage[1], David Chitayat[5,6], Karen Chong[6], Ingrid M. Wentzensen[7], Catherine Vincent-Delormé[8], Alban Lermine[9], Emma Burkitt-Wright[10], Weizhen Ji[11], Lauren Jeffries[11], Lynn S. Pais[12], Tiong Y. Tan[13,14], James Pitt[14,15], Cheryl A. Wise[16], Helen Wright[17,18], Israel D. Andrews[19], Brianna Pruniski[20], Theresa A. Grebe[20], Nicole Corsten-Janssen[21], Katelijne Bouman[21], Cathryn Poulton[22], Supraja Prakash[20], Boris Keren[23], Natasha J. Brown[13,14], Matthew F. Hunter[24,25], Oliver Heath[13,26], Saquib A. Lakhani[11], John H. McDermott[10,27], David B. Ascher[3,4], Gavin Chapman[1,2], Kayleigh Bozon[1], Sally L. Dunwoodie[1,2,28]

[1]Victor Chang Cardiac Research Institute, Darlinghurst, Sydney, NSW 2010, Australia.
[2]School of Clinical Medicine, Faculty of Medicine and Health, Sydney, NSW 2052, Australia.
[3]School of Chemistry and Molecular Biosciences, University of Queensland, Brisbane, Queensland 4072, Australia.
[4]Computational Biology and Clinical Informatics, Baker Heart and Diabetes Institute, Melbourne, Victoria 3004, Australia.
[5]Department of Pediatrics, Division of Clinical and Metabolic Genetics, The Hospital for Sick Children, University of Toronto, Toronto, ON M5G 1X8, Canada.
[6]The Prenatal Diagnosis and Medical Genetics Program, Department of Obstetrics and Gynecology, Mount Sinai Hospital, University of Toronto, Toronto, ON M5G 1X5, Canada.
[7]GeneDx, Gaithersburg, MD 20877, USA.
[8]Clinique de Génétique "Guy Fontaine", Hôpital Jeanne de Flandre, Lille 59000, France.
[9]Laboratoire de Biologie Médicale Multisites SeqOIA, FMG2025, FMG2025, Paris 75014, France.
[10]Manchester Centre for Genomic Medicine, St Mary's Hospital, Manchester University Hospitals NHS Foundation Trust, Manchester, M13 9WL, UK.
[11]Yale University School of Medicine, Pediatric Genomics Discovery Program, New Haven, CT 06520, USA.
[12]Broad Institute of MIT and Harvard, Cambridge, MA 02142, USA.
[13]Victorian Clinical Genetics Services, Murdoch Children's Research Institute, Melbourne, Victoria 3052, Australia.
[14]Department of Paediatrics, The University of Melbourne, Parkville, Victoria 3052, Australia.
[15]Metabolic Laboratory, Victorian Clinical Genetics Services, Murdoch Children's Research Institute, Melbourne, Victoria 3052, Australia.
[16]Department of Diagnostic Genomics, PathWest Laboratory Medicine Western Australia, Nedlands, Perth, Western Australia 6009, Australia.
[17]General Paediatric Department, Perth Children's Hospital, Perth, Western Australia 6009, Australia.

[18]Rural Clinical School, University of Western Australia, Perth, Western Australia 6009, Australia.
[19]Pinnacle Dermatology, Scottsdale, AZ 85258, USA.
[20]Division of Genetics and Metabolism, Phoenix Children's Hospital, Phoenix, AZ 85006, USA.
[21]Department of Genetics, University Medical Centre Groningen, University of Groningen, Hanzeplein 1, 9700RB Groningen, The Netherlands.
[22]Genetic Services of Western Australia, King Edward Memorial Hospital, Perth 6008, Australia.
[23]Département de Génétique, Groupe Hospitalier Pitié-Salpêtrière, Assistance Publique - Hôpitaux de Paris, Sorbonne Université, Paris 75013, France.
[24]Monash Genetics, Monash Health, Clayton, Victoria 3168, Australia.
[25]Department of Paediatrics, Monash University, Clayton, Victoria 3168, Australia.
[26]Department of Metabolic Medicine, The Royal Children's Hospital, Melbourne, Victoria 3052, Australia.
[27]The Division of Evolution, Infection and Genomics, School of Biological Sciences, University of Manchester, Manchester, UK.
[28]Faculty of Science, University of New South Wales, Sydney, NSW 2052, Australia.

EMMA, DS, and KI contributed equally to this work.

Address correspondence to:

Sally L. Dunwoodie at the Victor Chang Cardiac Research Institute, Lowy Packer Building, 405 Liverpool St., Darlinghurst, Sydney NSW 2010, Australia. Tel. (612) 9295 8613, email: s.dunwoodie@victorchang.edu.au

TABLE OF CONTENTS

Content	Page
**Supplemental Materials and Methods**	5
**Supplemental Figures**	14
Figure S1. Mammalian NAD synthesis pathways	14
Figure S2. Structural representation of patient NADSYN1 variants within a NADSYN1 monomer	15
Figure S3. Human wild-type and variant NADSYN1 protein expressed in COS-7 cells and purified	16
Figure S4. Deletion schematic for the generation of a *Nadsyn1*-null allele in the mouse strain C57BL/6J.	17
Figure S5. NADSYN1 enzymatic activity generating NAD in *Nadsyn1* wild-type, heterozygous, and homozygous-null liver tissue extracts of adult mice	18
Figure S6. Congenital malformations in E18.5 *Nadsyn1-/-* embryos	19
Figure S7. Weight change in female mice maintained on the *Standard Diet* or *Sufficient Diet*	20
Figure S8. Weight change in non-pregnant female *Nadsyn1+/-* and *Nadsyn1-/-* mice maintained on various dietary conditions for 17 days	21
Figure S9. Phenotypic outcomes of E17.5 embryos generated under various dietary conditions and mating schemes	22
Figure S10. Phenotypic outcomes of E17.5 embryo litters generated under various dietary conditions and mating schemes	23
Figure S11. Malformations found in E17.5 embryos generated from *Nadsyn1+/-* intercrosses with females given a *Sufficient Diet* or *Limited Diet* during pregnancy	24
Figure S12. Levels of NAD-related metabolites in plasma of female mice of different *Nadsyn1* genotypes under various dietary conditions	25
Figure S13. Malformations found in E17.5 embryos generated from matings of *Nadsyn1-/-* females with *Nadsyn1+/-* males, and females given a *Sufficient Diet* or *Limited Diet* supplemented with NMN during pregnancy	26
Figure S14. Malformations found in E17.5 embryos generated from *Nadsyn1+/-* intercrosses with females given a *Limited Diet* with different supplements during pregnancy	27

Figure S15. Pregnant mouse plasma NAD-related metabolites, maternal liver total NAD, and total NAD in generated E11.5 embryos 29

Figure S16. Number of surviving and dead embryos at E11.5 under various dietary conditions and mating schemes 30

Supplemental Tables 31

Table S1. Expanded phenotypic description of patients with biallelic *NADSYN1* (NM_018161.5) variants 31

Table S2. Predicted impact of NADSYN1 missense mutation in silico to substrate binding affinities 35

Table S3. Concentrations of NAD and related metabolites in patients with biallelic *NADSYN1* variants and their parents 34

Table S4. Plasma Global Metabolomic Assisted Pathway Screen of F4.II.2 36

Table S5. Urinary NAD metabolome components in Family 7 prior to and post-NAM supplementation 39

Table S6. Mouse dietary treatments, their NAD precursor contributions, and how they compare to the human recommended dietary intake 40

Table S7. Incidence of embryo loss and malformation in E18.5 embryos generated on the *Standard* and *Limited Diets* 41

Table S8. Summary of types and incidence of congenital malformations in E17.5 mouse embryos 44

Table S9. Liver total NAD levels of female mice of different *Nadsyn1* genotypes under various dietary conditions 45

Table S10. Concentrations of NAD and related metabolites in plasma of female mice of various *Nadsyn1* genotypes fed various NAD precursors for 17 days 46

Table S11. Concentrations of NAD and related metabolites in plasma of pregnant *Nadsyn1+/-* and *Nadsyn1-/-* mice at 11.5 days of gestation 47

Table S12. Total NAD levels in E11.5 embryos generated under various dietary conditions and mating schemes 48

Table S13. NADSYN1 cloning and genotyping primers 49

Supplemental Results 50

Molecular modelling 50

Clinical Presentation of Patients 52

Supplemental Materials and Methods

DNA sequencing and analysis

F1.II.1

Using genomic DNA from the proband (F1.II.1) and parents (F1.I.1 and F1.I.2), the exonic regions and flanking splice junctions of the genome were captured using the SureSelect Human All Exon V4 (50 Mb), the Clinical Research Exome kit (Agilent Technologies, Santa Clara, CA) or the IDT xGen Exome Research Panel v1.0 (Integrated DNA Technologies, Coralville, IA). Massively parallel (NextGen) sequencing was done on an Illumina system with 100 bp or greater paired-end reads. Reads were aligned to human genome build GRCh37/UCSC hg19, and analyzed for sequence variants using a custom-developed analysis tool. Reported variants were confirmed by an appropriate orthogonal method in the proband and in selected relatives. Additional sequencing technology information and variant interpretation protocol has been previously described (1). The general assertion criteria for variant classification are publicly available on the GeneDx ClinVar submission page (http://www.ncbi.nlm.nih.gov/clinvar/submitters/26957/)

F2.II.3

The proband and parents were sequenced using an Illumina NovaSeq system. The quantification and the qualification of the nucleic acids were obtained on a Spark multimode reader (Tecan) and Fragment Analyzer (Agilent), respectively. The fragments were generated by sonication (LE220-plus, Covaris). Size selection, as well as subsequent purification steps, were carried out on magnetic beads (Sera-Mag magnetic beads, GE Healthcare). The library was prepared without amplification (NEBNext Ultra II End repair/A-tailing module & Ligation module, New England Biolabs). The library was quantified by qPCR (NEBNext Custom 2X Library Quant Kit Master Mix, New England Biolabs; QuantStudio 6 Flex Real-Time PCR System, Life Technologies). The libraries were sequenced in "paired-end" (2 times 150 cycles) by SBS technology (Flow Cell S4, NovaSeq 6000, Illumina).

Raw (.BCL) Illumina files were converted into FASTQ files with the bcl2fastq Illumina conversion software (v2.20.0.422). The reads were aligned to the GRCh38 Human reference (GRCh38, release-92, Jul 02 2018, ftp.ensembl.org) with the BWA-MEM (v0.7.15) algorithm from the Burrows-Wheeler Aligner (BWA) package. Next, duplicate reads were flagged with Picard MarkDuplicates (2.8.1) and base quality recalibration was performed with GATK4 (v4.1.6.0, Broad Institute). The SNPs and DELINS were called with HaplotypeCaller (GATK4, v4.1.7.0, Broad Institute) and annotated with SNPeff (4.3t) and SnpSift (4.3t); the selected database were SNPEff (v4.3t), 1000Genomes (phase3, v2013-05-02), gnomAD (exomes, v2.1.1), gnomAD (genomes, v3), ClinVar (v20190722), COSMIC (coding, v89), COSMIC (non-coding, v89), dbscSNV (v1.1), dbSNP (v20180418), dbNSFP (v4.0) and phastCons (v08-May-2015). Finally, Copy Number Variants (CNVs) were called with CNVnator (v0.4.1, Mark B. Gerstein lab, Yale University); CNVs were annotated with AnnotSV (v2.5.1) using an integrated database (v2.5).

Candidate *NADSYN1* variants were identified with a compound heterozygous filter including DP > 6, GQ > 0, QD > 3, population frequency within an internal database

<1% and homozygous within this database <3, homozygous in gnomAD <3, and impact on the protein scoring high, moderate, and low with respect to SNPeff.

F3.II.3

Due to the combination of abnormalities identified in F3.II.3, eligibility was met for rapid trio exome sequencing (R14) of the proband and parents, a National Health Service England clinically accredited test (ISO15189) offered through the Exeter Genomics Laboratory for children born with suspected monogenic disorders admitted to the NICU (https://www.exeterlaboratory.com/test/exome-sequencing-services/ (accessed 5th September 2022)). In brief, DNA was extracted from whole blood of the proband and parents and prepared for sequencing as per manufacturer's instructions (Twist Biosciences), sequenced on a NovaSeq Sequencer (Illumina, San Diego, CA, USA) averaging 98.8% of nucleotides in RefSeq targeted exons (https://www.ncbi.nlm.nih.gov/refseq) with a coverage ≥20, covering the coding regions and conserved splice site regions of 23244 genes. Analysis interrogated single nucleotide variants and indels (up to 30 bp) with coverage of ≥20 reads at a sensitivity of 99.89% (95% CI 99.88-99.91%) and 98.6% (95% CI 97.8-99.12%), respectively. Agnostic variant assessment was performed with filters applied to prioritize de novo or biparental homozygous variants. Variants were classified using the American College of Medical Genetics and Genomics and the Association for Molecular Pathology (ACMG/AMP) (2) and ACGS 2020 guidelines (https://www.acgs.uk.com/media/11631/uk-practice-guidelines-for-variant-classification-v4-01-2020.pdf, accessed 5th September 2022), resulting in the identification of the homozygous *NADSYN1* (NM_018161.5) c.145T>C p.C49R in the proband. The *NADSYN1* gene (2541 bp) was covered at a mean depth of 98.19 with 100% of the coding and splice site regions with a depth of ≥20. CNVs were also assessed using SavvyCNV (3) analysis software from whole exome sequence data. No reportable CNVs were identified.

F4.II.2

Genomic DNA was prepared from saliva using standard procedures. The whole exome was captured using the xGen target kit from IDT, and 101 base paired-end sequencing on the Illumina platform (HiSeq 4000) was performed under a research protocol at the Yale Center for Genome Analysis. The sequence reads were converted to FASTQ format and were aligned to reference human genome (hg19). GATK (4) best practices were applied to identify genetic variants, and variants were annotated by ANNOVAR (5). The trio samples from this family were sequenced to a mean coverage of 130-190x, with at least 20x independent reads in ~98% of targeted bases or 50x in ~97% of targeted bases.

Exonic or splice-site rare variants that exhibited high quality sequence reads (pass GATK Variant Score Quality Recalibration, had a minimum 10 total reads, and had a quality of depth score [QD] ≥ 2) were filtered for MAF ≤ 0.01 across all samples in gnomAD all or subpopulations, and within the Yale institutional database. De novo variants that were only present in the proband's sample (≥20% alternate allele frequency in the proband, alternate allele frequency < 3% in parents) were called. Rare homozygous or compound heterozygous variants were called if each was inherited from one of the parents, respectively. All the recorded variants were then visualized and verified manually by Integrative Genomics Viewer (IGV). The homozygous variant NM_018161.5(*NADSYN1*):c.1717G>A p.(A573T) had a depth of 123 independent reads in the proband.

F5.II.1

Familial whole exome sequencing was performed on DNA from whole blood samples of the proband and parents. Next Generation Sequencing was performed at Randwick Genomics Laboratory (NATA accredited to ISO15189). Library preparation was performed using an Agilent SureSelect XT Low Input Clinical Research Exome x3 (Agilent CRE v2) kit, with libraries analyzed on an Illumina NovaSeq 6000, at a multiplex of 96 exomes per S2 flow cell. Data filtering was performed using an in-house pipeline (Genomics Annotation and Interpretation Application; GAIA, v.3).

Reads were aligned to Human Genome Reference Sequence GRCh38 and single nucleotide and short insertion/deletion variants identified using the Dragen Server in Illumina Basespace. Variant filtering, prioritization, and reporting was performed using the GAIA in-house pipeline under assumptions of heterozygous de novo, heterozygous autosomal dominant, homozygous and compound heterozygous autosomal recessive, X-linked de novo, and X-linked inheritance patterns. Genomic variants were filtered based on Gemini impact, on frequencies of <2% in the gnomAD databases for homozygous and compound heterozygous models and <0.1% for heterozygous models, and a minimum threshold CADD-PHRED score of 10. Analysis was limited to variants present within an in-house variant database, and those OMIM/Orphanet or Pubmed-defined disease genes where variants are rare or unique to the family based on in-house datasets, and segregation is consistent with the proposed modes of inheritance. The data analysis pipeline was based on Gemini v18 with annotation from VEP and dbSNFP, and leveraged family structure and known inheritance patterns.

Evidence for phenotype-causality was evaluated for each variant resulting from the aforementioned filtering strategies and variants were prioritized according to laboratory criteria (6). Only those variants with evidence for causing or contributing to disease were reported. Each variant was evaluated based on the available information from the following databases (typically including, but not limited to, the public version of ClinVar, LSDBs and gnomAD v2.1.1 and v3), published literature, clinical correlation, segregation analysis, functional studies, and its predicted functional or splicing impact using evolutionary conservation analysis and computational tools (including PolyPhen-2, SIFT, CADD, REVEL, Provean, ReVe, and Clinpred, and structural information from Metadome, PDB/Uniprot, and HOPE).

Variants were classified following the joint consensus recommendations of the ACMG/AMP for the interpretation of sequence variants (2) as well as integrating guidance from the best practice guidelines for variant interpretation 2019 and 2020 published by the Association for Clinical Genomic Science (https://www.acgs.uk.com/quality/best-practice-guidelines/). Variants were reported according to HGVS nomenclature (www.hgvs.org/mutnomen).

F6.II.2

Whole exome sequencing and data processing were performed by the Genomics Platform at the Broad Institute of MIT and Harvard with an Illumina Twist exome capture (~38 Mb target) and sequenced (150 bp paired reads) to cover >80% of targets at 20x and a mean target coverage of >100x. Exome sequencing data was processed through a pipeline based on Picard and mapping done using the BWA aligner to the human genome build 38. Variants were called using Genome Analysis Toolkit (GATK) HaplotypeCaller package version 3.5.

F7.II.3

Whole exome sequencing with DNA from the proband and both parents was performed using massively parallel sequencing (Twist VCGS whole exome capture) with a minimum coverage of 97% of bases sequenced to at least 10×. Data was processed, including read alignment to the reference genome (GRCh38) and variant calling, using Cpipe (7).

Automated sex determination, relatedness, and contamination checks were performed.

Variant analysis and interpretation within the target region (coding exons +/- 8 bp) was performed using Agilent Alissa Interpret. Variants were annotated against relevant RefSeq gene transcripts, curated utilizing the transcript predicted to be the most deleterious to the protein and reported in accordance with HGVS nomenclature. Genomic coordinates were generated by Cpipe and do not necessarily comply with HGVS guidelines. Annotations included ClinVar, OMIM, gnomAD v2.1.1, gnomAD v3.1, gene constraint scores (gnomAD), NCBI, UniProt, UCSC Genome Browser, DECIPHER, PolyPhen2, PROVEAN, MutationAssessor, FATHMM, PhyloP, NetGene2, BDGP NNSPLICE.

Curation of variants was phenotype-driven with pre-curated or custom gene lists used for variant prioritization. The following PanelApp Australia Gene lists were applied: *Growth failure v1.20, Microcephaly v1.70, Congenital Heart Defect v0.142, Intellectual disability syndromic and non-syndromic v0.4288, Mendeliome v0.9771* (https://panelapp.agha.umccr.org/). If a likely cause of the disease was identified, other candidate variants may not have been considered. Where no causative variants were identified within the prioritized genes, analysis was expanded to truncating and very rare/conserved missense variants identified in the Mendeliome that could potentially be associated with the reported phenotype (this applied only to cases where appropriate request and consent were supplied). Classification of variants was based on ACMG guidelines (2). Reported high confidence variants were generally not confirmed by an orthogonal method.

F8.II.3

The index case (F8.II.3) was tested as a singleton using DNA extracted from an EDTA blood sample and whole exome massively parallel sequencing using the TWIST Comprehensive Exome panel (102032) sequenced on an Illumina NextSeq550 with bioinformatic analysis performed using DRAGEN Enrichment v3.6.3 aligned to human reference sequence GRCh38.

The VCF file was assessed by applying HPO terms for Rib fusion (HP:0000902), Abnormal vertebral morphology (HP:0003468), Camptodactyly of the 2nd-5th fingers (HP:0001215) and Posterior rotated ears (HP:0000358).

Variants were filtered using gnomAD v2 allele frequencies (initial filtering to remove variants with >2% MAF), HPO match score, presence in clinical databases (ClinVar) as likely pathogenic/pathogenic, variant type - nonsense/frameshift/translation initiation/translation termination variants, variants with potential splice effect including intronic variants within 5 bp of exons and missense variants assessed based on in silico predictions, mode of inheritance for the gene and further refinement of gnomAD allele frequencies. The “apparently” homozygous *NADSYN1* c.1717G>A missense variant only associated with one HPO term (HP:0003468), the gene was assessed as being associated with autosomal recessive vertebral, cardiac, renal, and limb abnormalities (OMIM #618845), the variant altered a highly conserved

amino acid and in silico prediction algorithms (MutationTaster, PolyPhen2, SIFT) supported a deleterious effect for the missense change, observed in gnomAD at a frequency consistent with a recessive allele (0.074%, with no homozygotes present) and was cited in ClinVar as well as reported in the literature in the homozygous state in two affected siblings and in compound heterozygosity with a frameshift variant in a similarly affected unrelated individual with in vitro studies in yeast suggesting the variant has a damaging effect (8).

Subsequent parental testing on DNA extracted from EDTA blood samples was performed using targeted Sanger sequencing - the *NADSYN1* variant was detected in the heterozygous state in each parent, confirming biparental inheritance (true homozygosity) of the variant in the index case. Following this, DNA extracted from amniotic fluid samples from two previously affected fetuses were also tested using targeted Sanger sequencing and the variant was detected in the homozygous state in each sample (F8.II.1 and F8.II.2).

F9.II.4

Genomic DNA from buccal swaps of all available family members was used for whole exome sequencing, CNV calling, and analysis by GeneDx as described for Patient F1.II.1. No clinically relevant deletion or duplication of three or more exons of any gene were identified from this analysis. 100% of the coding region of *NADSYN1* was covered with >10 read depth.

F10.II.2

Trio whole exome sequencing (NextSeq Sequencer, Agilent SureSelect Human All Exon V6; 151 bp paired-end reads) was performed on DNA from patient (F10.II.2) and parental buccal swaps. Gene analysis filtered variants based on Mendelian inheritance assessing heterozygous, compound heterozygous, recessive, and de novo variants. All variants, regardless of inheritance pattern and parental carriership, were interpreted. Filtered variants were finally reviewed by the clinician and classified for further assessment according to ACMG (2) guidelines.

Protein modelling and computation

The structure of the NADSYN1 monomer was downloaded from the Alphafold2 database (9) and superimposed on the human crystallized complex (PDB: 6OFB) (10) in PyMOL to create the full homooctamer. Ligand NaAD was transposed to the Alphafold2 complex from the crystallized structure, while ATP was docked using Glide (Schrodinger suite v.11.4) based on the transposed coordinates of the crystallized AMP and POP obtained from PDB 6OFB. Ligand glutamine was transposed from the *M. tuberculosis* NADSYN1 structure (PDB 6OFC), along with a water molecule present at the binding site, and minimized using the Protein Preparation Wizard (Schrodinge suite v.11.4) to remove clashes. The prepared structures were used as inputs for the estimation of changes in affinity to ligands NaAD, ATP, and glutamine (mmCSM-lig). In analyzing each affinity change perturbation, only values for variants within 12 Å of the ligand were assessed to account for direct effects at the variant locus. Finally, the interaction patterns of each variant site were visualized and generated using the Arpeggio (11) webserver, for both wild-type and PyMOL-generated variant proteins.

Cloning of human expression constructs, protein expression, purification, and immunoblotting

Human wild-type NADSYN1 pcDNA3.1 myc-His and a version harboring the Ala573Thr variant were generated as described (8); additional NADSYN1 mutants (Arg127Cys, Cys175Tyr, Ala363Val, Asp587Asn) were generated by mutagenesis of pcDONR201 NADSYN1 with primers (Supplemental Table 10) and Gateway LR cloned (Gateway LR Clonase, Invitrogen) into pcDNA3.1 GW myc-His as described for the Ala573Thr variant (8). Wild-type NADSYN1 and variant proteins were purified from transiently transfected COS-7 cells, and expression quantified via immunoblot detected with the anti-c-Myc antibody (clone: 9E10, Invitrogen) as described (8).

Enzymatic assessment of purified NADSYN1

Enzymatic assessment of wild-type NADSYN1 and variant proteins in the presence glutamine was performed as described (8). Enzymatic activity in the presence of ammonia was assessed using an identical protocol with *L*-glutamine substituted with 200 mM NH_4Cl.

***Nadsyn1*-null mouse generation and genotyping**

Nadsyn1-null (*Nadsyn1-/-*) mice were produced by the Mouse Engineering Garvan/ABR (MEGA) Facility (Moss Vale and Sydney, Australia) using CRISPR/Cas9 gene targeting in C57BL/6J mouse embryos following established molecular and animal husbandry techniques (12). The single guide RNA (sgRNA) employed targeted within exon 11 of *Nadsyn1* (TCCACCGTCACCCGGGGATATGG, protospacer-associated motif is underlined). A solution consisting of sgRNA (15 ng/µL) and full length, polyadenylated *S. pyogenes* Cas9 mRNA (30 ng/µL) was prepared and microinjected into the nucleus and cytoplasm of C57BL/6J zygotes. Microinjected embryos were cultured overnight and those that underwent cleavage introduced into pseudo-pregnant SwissTac females. Pups were screened at weaning for disruption to the *Nadsyn1* gene. A founder was identified carrying a 19 bp deletion in exon 11 of *Nadsyn1*: NM_030221, causing premature termination of translation (*Nadsyn1* c.del880_898 p.Gly293Argfs*1). Mice were genotyped via triple primer PCR amplification of 445 bp spanning the site of the deletion, and either internal WT or mutant allele (Supplemental Table 13). Mutant mice were backcrossed onto C57BL/6J mice at least 4 times.

Mouse dietary treatments and sample collection

Mice for breeding and male mice used for timed mating were maintained on Rat and Mouse Premium Breeder Diet (Gordon's Specialty Feeds, Yanderra, Australia). Female mice for timed matings were fed nicotinic acid (NA)-rich AIN93G ("*Standard Diet*"; (13)) or a modified AIN93G diet (SF22-100; Specialty Feeds, Glen Forrest, Australia) containing 30 mg/kg nicotinamide (NAM) and 1800 mg/kg *L*-tryptophan as NAD precursors, defined as *Sufficient Diet*, for at least 3 weeks prior to mating (Supplemental Table 6).

From the observation of copulation plugs, defined as embryonic day (E) 0.5, pregnant female mice were either maintained on the *Standard Diet* or *Sufficient Diet*, or put on a *Limited Diet* comprised of 1.4 mg/kg nicotinamide as the sole NAD precursor (SF16-097; Specialty Feeds, Glen Forrest, Australia) until dissection. Under *Limited Diet* conditions, drinking water was supplemented with 600 mg/L *L*-tryptophan (Sigma). B3 vitamers were added to the tryptophan water mixes at 121.8

µM [15 mg/L] NA (Sigma), nicotinic acid mononucleotide (NaMN) or nicotinamide mononucleotide (NMN; GeneHarbor Biotechnology, Hong Kong) (Supplemental Table 6) and refreshed every 7 days. At E11.5, E17.5, and E18.5, pregnant mice were sacrificed by CO_2 asphyxiation and cervical dislocation, and embryos collected for NAD quantification or phenotyping, respectively. At E11.5, maternal plasma, and liver were also collected as described (14). Additional non-pregnant female mice pre-treated for at least 3 weeks on *Sufficient Diet* were maintained on aforementioned diets and plasma and liver samples were collected from these mice after 17 days of treatment. All samples were stored at -80°C prior to enzymatic or metabolomic assessment.

Liver NADSYN1 activity assays

To assess the specific activity of NADSYN1 in mouse tissues, frozen liver was ground under liquid nitrogen-cooled mortar and pestle and 100 mg of tissue sonicated and centrifuged in HEPES/KOH buffer pH 7.5, with 20 mM NaF, 5 mM $MgCl_2$, 0.5 mM DTT and 0.5 mM PMSF Mori, et al. (15). 2.5 µL of lysate was assayed for NADSYN1 activity in 20 µL reactions of 50 mM potassium phosphate buffer, pH 7.5, 100 mM KCl, 20 mM NaF, 5 mM $MgCl_2$, 4 mM ATP, 20 mM L-glutamine, and 1 mM NaAD Mori, et al. (15). Reactions were halted on ice and immediately assayed for NAD(H) content.

Embryonic NAD and maternal liver NAD quantification

Measurements of total NAD in liver and E11.5 embryos was performed as described (16).

Embryo phenotyping

E17.5 and E18.5 embryos were dissected in ice-cold phosphate-buffered saline (Sigma), assessed for external malformations as previously described (16) and weighed. E18.5 embryos were then processed for micro-CT scanning as previously described (17). E17.5 embryos were washed in ice-cold water then transferred to a solution containing 100 mM Tris pH 8.8, 200 mL NaCl, 5 mM EDTA, 0.2% SDS, 100 µg/mL Proteinase K and incubated at 37˚C for between 5 to 100 mins depending on embryo weight and degree of external malformation. Embryos were further washed in water and transferred to a 0.7% PTA 70% ethanol solution and left on a tube roller at room temperature for >4 weeks, with PTA-ethanol solution replaced twice a week. Prior to micro-CT scanning, embryos were suspended in 1% agarose gel in polypropylene conical tubes to prevent sample movement during rotation mid-scan. E17.5 embryo scanning was performed on a Skyscan 1272 high-resolution 3D X-ray microscope (Bruker) operating at a 75 kV source voltage, 133 µA source current, using an Al 0.5 + Cu 0.038 mm filter. Magnification was set at a pixel size of 7.4 µm and images taken at 0.4° increments throughout a 360° rotation, 2-3 fields of view, and 2500 ms exposure. E18.5 embryos were scanned at a 65 kV source voltage, 153 µA source current, Al 1 mm filter, pixel size of 10 µm, and 1400 ms exposure time with other parameters constant. Micro-CT images were then reconstructed from acquired projections into 3D images using NRecon software (Bruker) with smoothing, ring artefact, and post-alignment corrections. 3D embryo reconstructions were then analyzed for internal malformation by manual inspection using CTVox (v3.3.0; Bruker) software interrogated for malformations of the skull, brain, eyes, palate, abdominal wall, heart, lungs, kidneys, limbs, digits, and tail. Malformations

were verified by complementary examination using DataViewer (v1.5.6.10, Bruker). Kidney length measurements were made using DataViewer.

NAD Metabolome assessment of human and mouse samples

Human samples were collected from individuals in the morning after overnight fasting where possible. Blood was collected into a Heparin-coated tube (BD Vacutainer®), and half of this sample was transferred into another tube and centrifuged for 15 min at 2000 g and 4°C to obtain plasma. Aliquots of whole blood and plasma were snap frozen on dry ice. Mid-stream urine samples were centrifuged for 10 min at 2000 g and 20°C, filtered with a 0.2 µM pore size syringe filter, aliquoted, and snap frozen on dry ice. All sample aliquots were stored at -70 to -80°C until metabolite extraction and quantification.

NAD and related metabolites were quantified using UHPLC-MS/MS as described (14), with minor adjustments. For mouse samples, the published protocol was followed, except for an additional step to quantify high concentration metabolites, whereby an aliquot of 10 µL of the supernatant after addition of extraction solvent and centrifugation was diluted 1:10 with 100 mM ammonium acetate in water and measured by UHPLC-MS/MS. For low concentration metabolites, the remainder of supernatant was dried in a vacuum centrifuge at room temperature and metabolites reconstituted in 50 µL of 100 mM ammonium acetate before measurement by UHPLC-MS/MS, as described.

Human plasma and whole blood samples were prepared as described (14) with the following adjustments: sample starting material and extraction solvents were doubled (plasma: 100 µL of sample and 1000 µL of 80% methanol in water (v/v); whole blood: 100 µL of sample, 100 µL of water, and 800 µL of methanol:chloroform 1:1). Duplicate samples for high and low concentration metabolites, respectively, were prepared and measured as described above for mouse samples.

For human urine samples, 100 µL of urine were mixed with 100 µL of methanol, incubated at -80°C for 10 min, and centrifuged for 10 min at 14,000 g and 4°C. 10 µL of the supernatant were mixed with 5 µL of internal standard mix (a mix of 21 stable isotope-labeled NAD-related metabolites, as described (14)) diluted 1:4 (v/v) in water, and 85 µL of 100 mM ammonium acetate in water before measurement by UHPLC-MS/MS via the published method (14).

Metabolic data was analysed by partial least squares discriminant analysis (PLS-DA) and variable importance in projection (VIP) calculation using MetaboAnalyst (v5.0) (https://www.metaboanalyst.ca/) with metabolite concentration values as input. Missing values due to the concentration being below or above the quantification limit were replaced as follows: those below the limit were replaced by 1/1000 of the minimum positive value of the corresponding variable; those above the limit were replaced by the upper quantification limit for that variable (the concentration equivalent to the highest concentration metabolite standard that could be fit onto the calibration curve). All concentration values were log transformed, Pareto scaled, and plotted via the default parameters of MetaboAnalyst.

Statistics

Statistical analyses, including One-way ANOVA (Supplemental Figures 8 and 15C, Supplemental Tables 9 and 12), Student's *t*-test (Supplemental Figure 15D, Supplemental Tables 9 and 12), two-tailed 2×2 Fisher's exact test (Supplemental Figures 13A and 13B) and Chi-square test (Supplemental Figure 16), were

performed with GraphPad Prism (v.10.0.2). Fisher's exact test with Freeman-Halton extension for 3×2 contingencies (Supplemental Figures 11A, 11B, 14A, 14B, 14C, and Supplemental Table 7) were performed using an online tool (https://www.danielsoper.com/statcalc/, v4.0, accessed June 1st 2023). Significance was calculated, adjusting for multiple comparisons, at $p < 0.05$. Bar graphs indicate mean and standard deviation.

Supplemental Figures

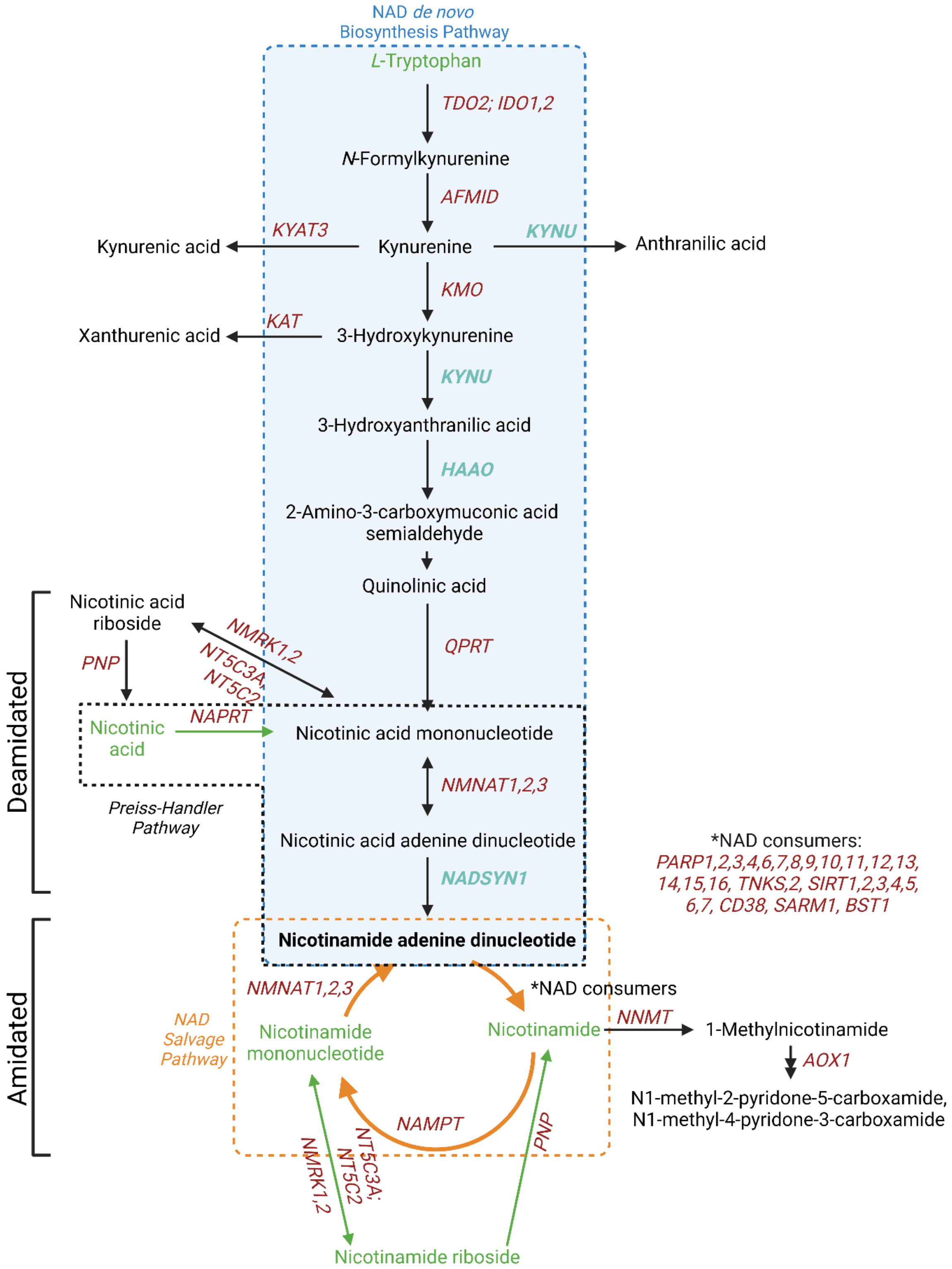

Supplemental Figure 1. Mammalian NAD synthesis pathways. Genes (red), dietary vitamin B3 precursors (green), genes in which biallelic loss-of-function mutations in patients can result in Congenital NAD Deficiency Disorder (cyan). Dotted lines indicate the NAD synthesis, Preiss-Handler, and salvage pathways. NAD: nicotinamide adenine dinucleotide.

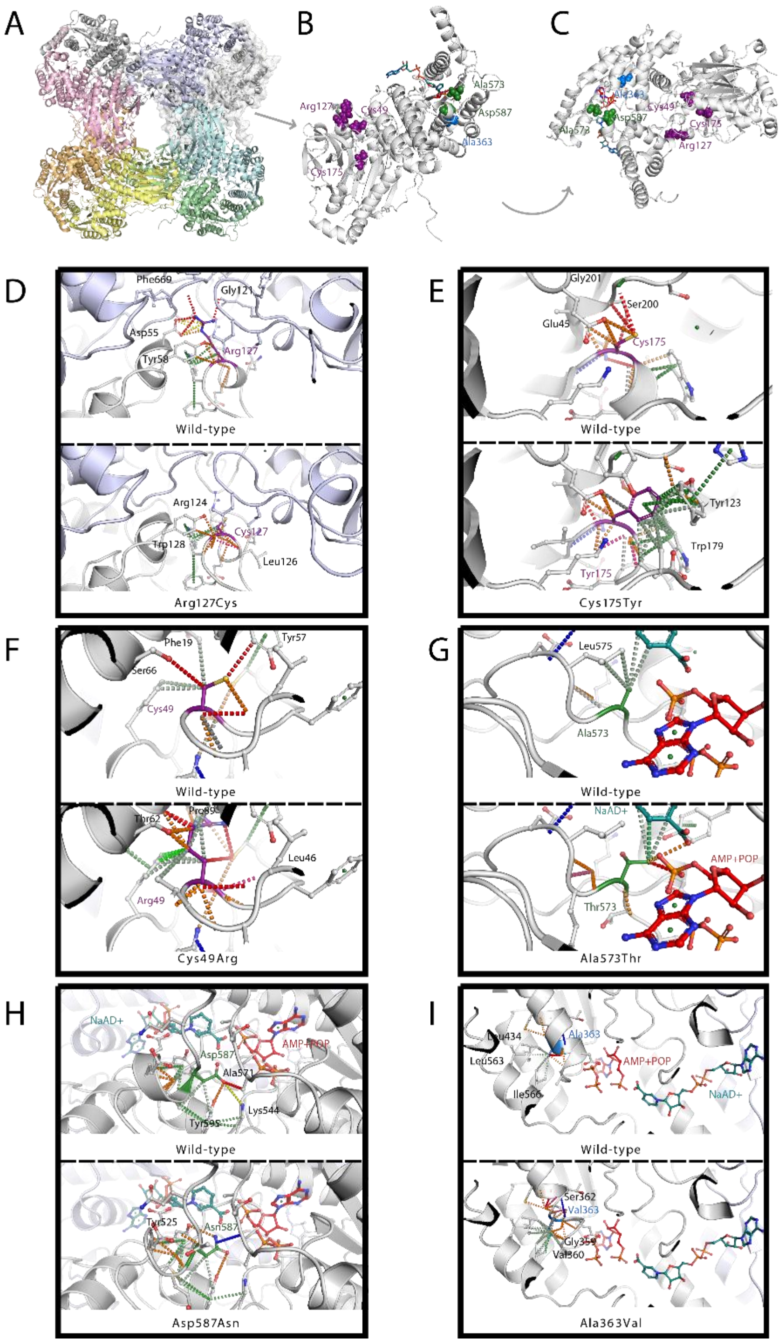

Supplemental Figure 2. Structural representation of patient NADSYN1 variants within a NADSYN1 monomer. The NADSYN1 complex is a homo-octamer (**A**), in which, variants identified in patients were introduced at residues Cys49, Arg127 and Cys175 (in purple) within the N-terminal glutaminase domain, Ala573 and Asp587 (dark green) within the C-terminal NAD synthetase domain, and Ala363 (blue) within the ATP-binding site (**B**, **C**). The latter three residues lie close to NaAD+ binding (teal) and ATP binding (AMP and pyrophosphate shown in red) shown as sticks (**B-C**, **G-I**). Residue interaction patterns for wild-type and variant amino acids are depicted (**D-I**), where hydrogen bonds are shown in red, polar interactions in orange, ionic interactions in yellow, amide-amide interactions in blue, carbonyl-van der Waals clashes in pink, aromatic interactions, including π-mediated interactions in dark green, and hydrophobic interactions in light green.

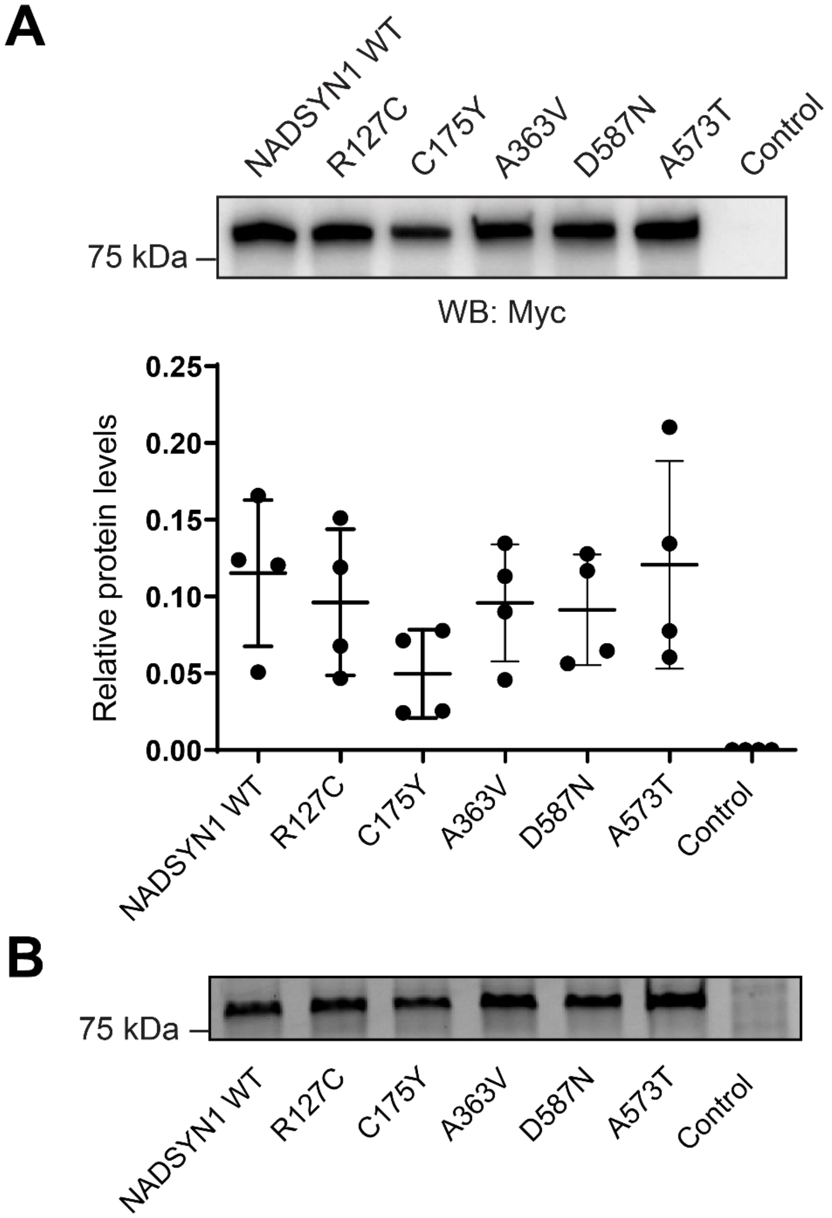

Supplemental Figure 3. Human wild-type and variant NADSYN1 protein expressed in COS-7 cells and purified. (**A**) Western blot detecting human wild-type and variant NADSYN1 protein lysates generated in transfected COS-7 cells and their levels relative to total lane protein, detected with an anti-Myc antibody. No significant difference between wild-type and variant groups was identified by One-way ANOVA. Bars indicate mean ± standard deviation. (**B**) Coomassie protein gel of purified wild-type and NADSYN1 mutant proteins used for enzymatic assessment. *n = 4*.

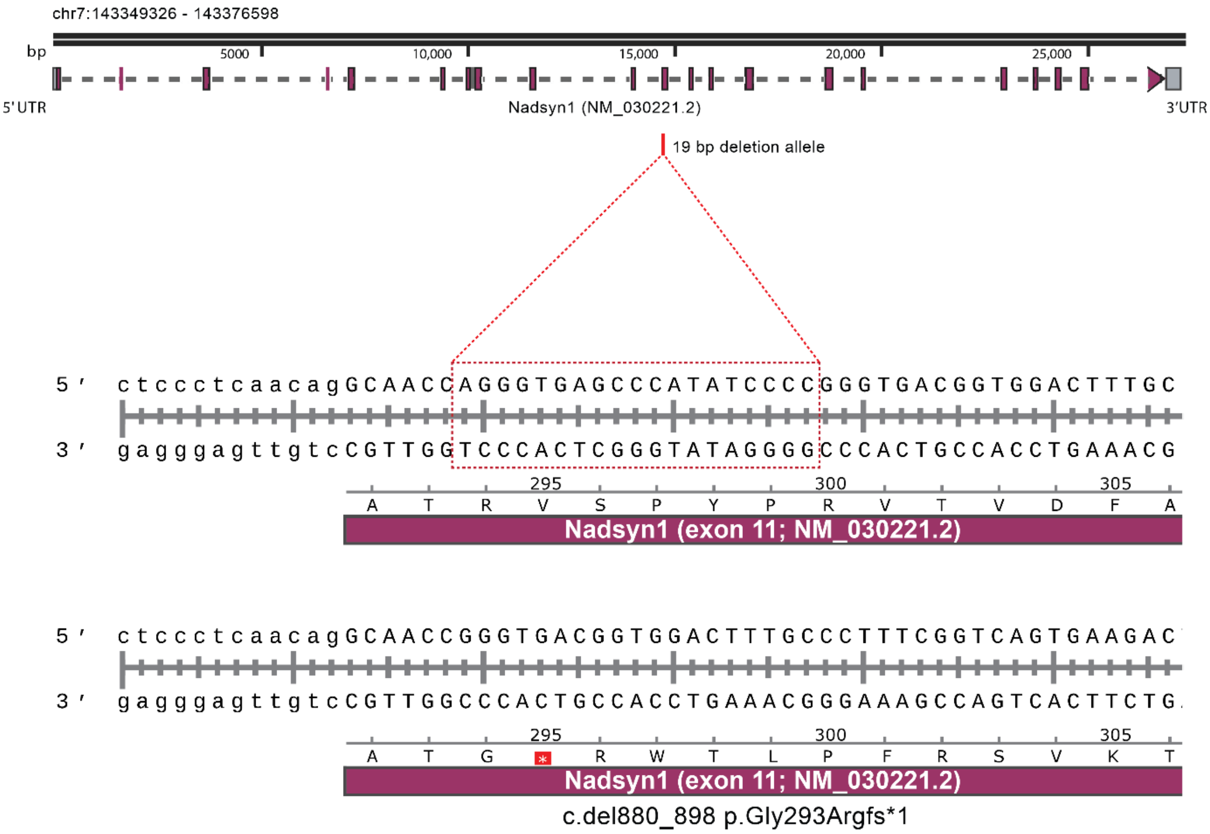

Supplemental Figure 4. Deletion schematic for the generation of a *Nadsyn1*-null allele in the mouse strain C57BL/6J. 19 bp deletion in *Nadsyn1* (NM_030221.2) exon 11 is bounded by a red dotted box.

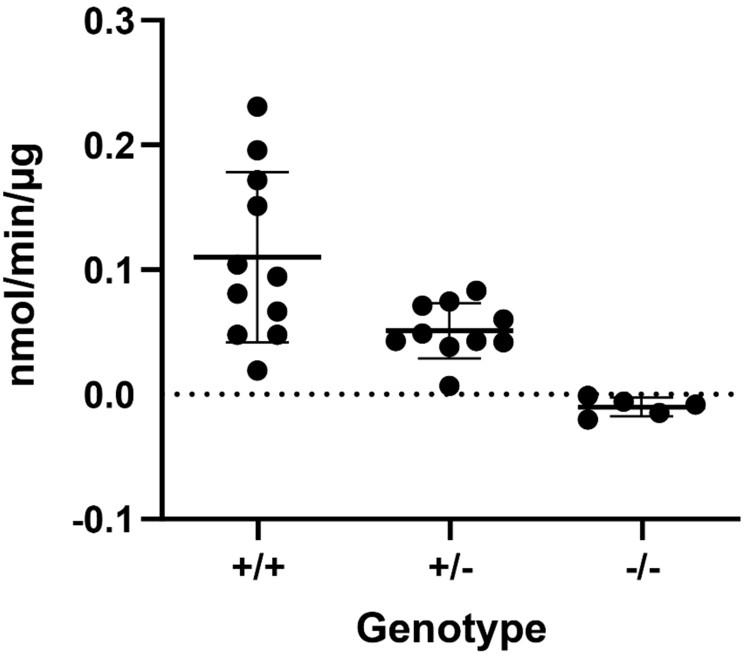

Supplemental Figure 5. NADSYN1 enzymatic activity generating NAD in *Nadsyn1* wild-type (+/+), heterozygous (+/-), and homozygous-null (-/-) liver tissue extracts of adult mice. Error bars indicate mean ± standard deviation. *n* = 5-11.

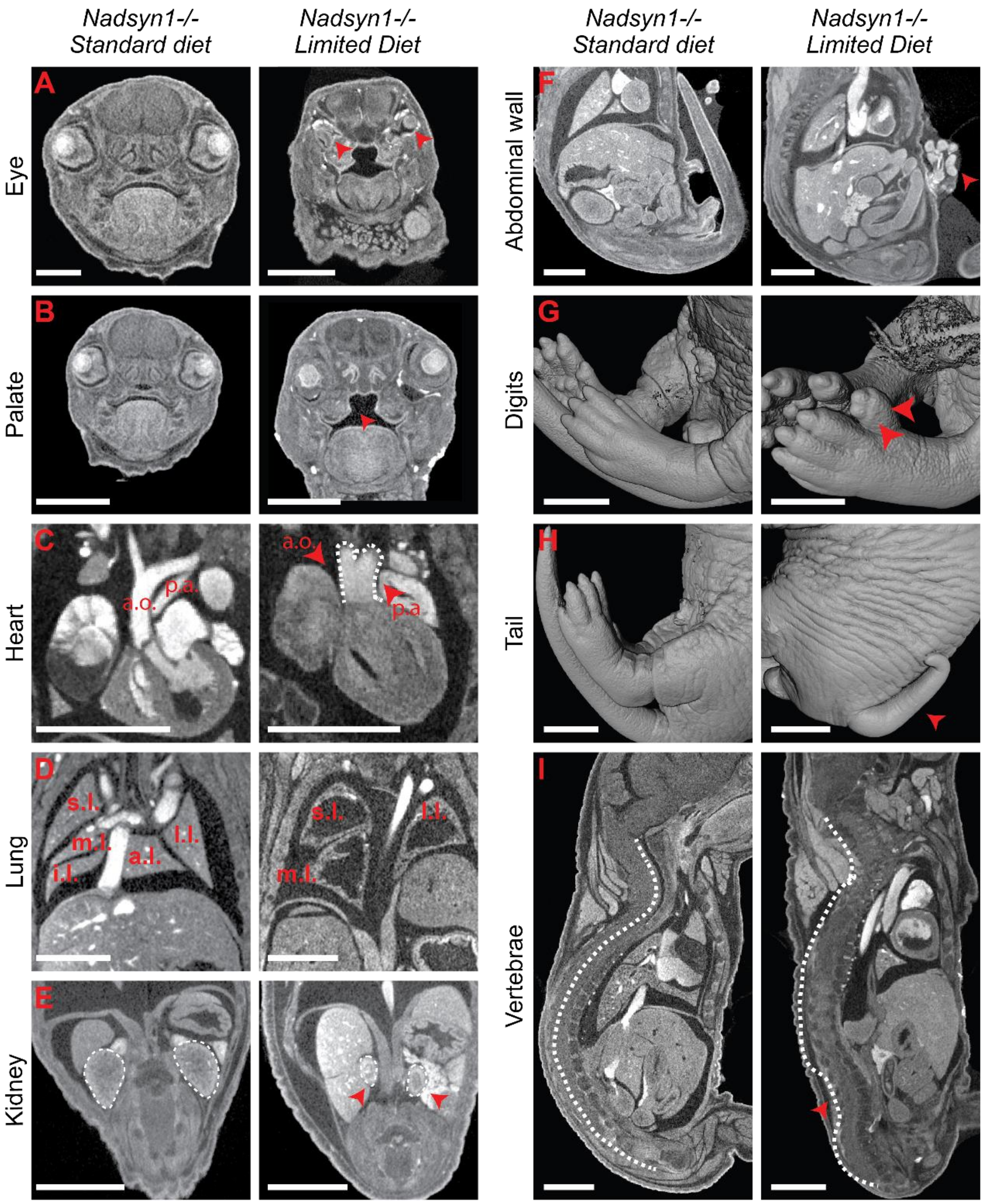

Supplemental Figure 6. Congenital malformations in E18.5 *Nadsyn1-/-* embryos. All embryos were generated from *Nadsyn1*+/- intercross with mothers provided with a *Standard Diet* or *Limited Diet* during pregnancy. Observed malformations (identified by red arrows and dotted white lines) reproducibly affected multiple systems, as labelled on the left of images, including the following: (**A**) Microphthalmia, coronal view; (**B**) Cleft palate, coronal view; (**C**) Persistent truncus arteriosus, coronal view. a.o.: aorta, p.a.: pulmonary artery; (**D**) Absent right auxiliary lung lobe (a.l.) and right inferior lung lobe (i.l.), coronal view. s.l.: superior lung lobe, m.l.: middle lung lobe, l.l.: left lung lobe; (**E**) Hypoplastic kidneys, coronal view; (**F**) Omphalocele, sagittal view; (**G**) Left hindleg 1st digit polysyndactyly; (**H**) Short, curly tail; (**I**) Scoliosis, sagittal view. Scale bar = 2 mm.

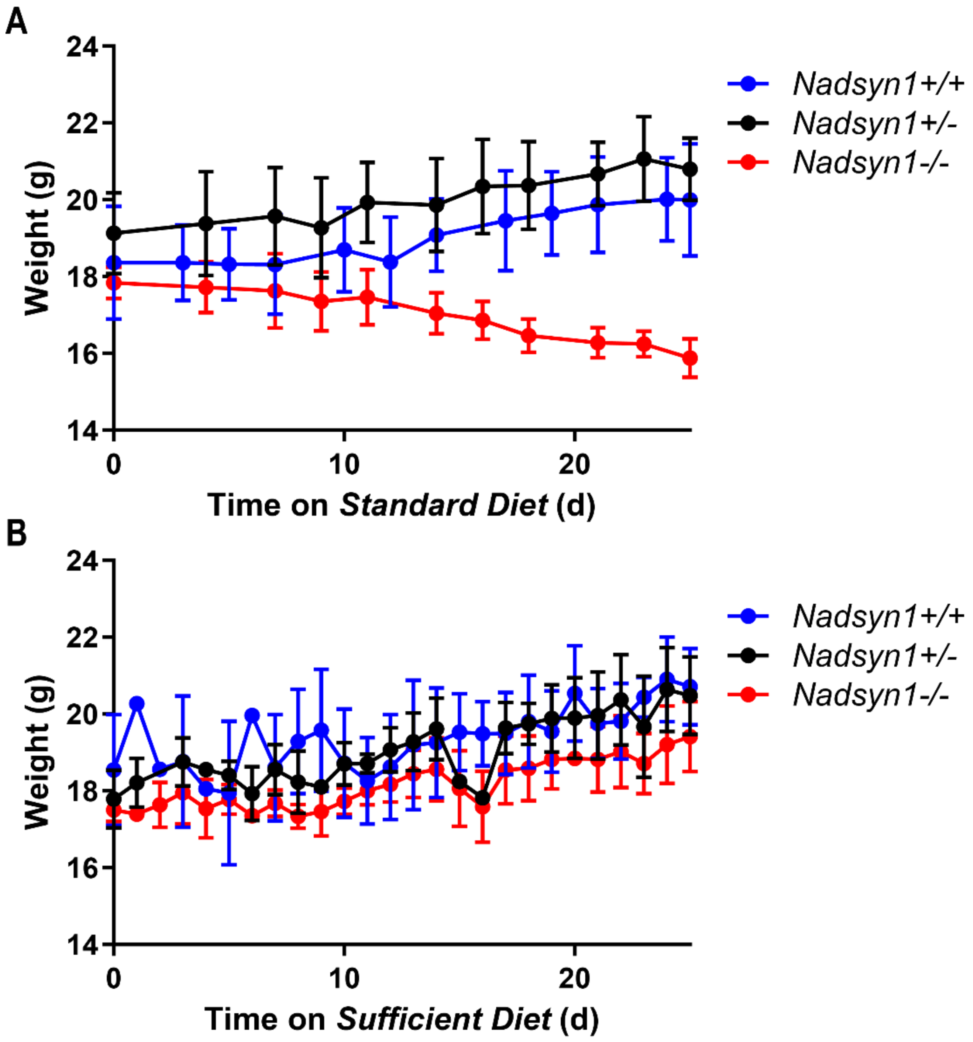

Supplemental Figure 7. Weight change in female mice maintained on the *Standard Diet* or *Sufficient Diet*. Female mice were fed either a (**A**) *Standard Diet*; or (**B**) *Sufficient Diet*, for 25 days with weight measurements taken every few days at 9 am. $n \geq 5$ mice per *Nadsyn1* genotype were treated per condition. Data points indicate mean ± standard deviation. Average weight changes in mice treated with the *Sufficient Diet* over the first 17 days are also represented in Supplemental Figure 8 as blue points.

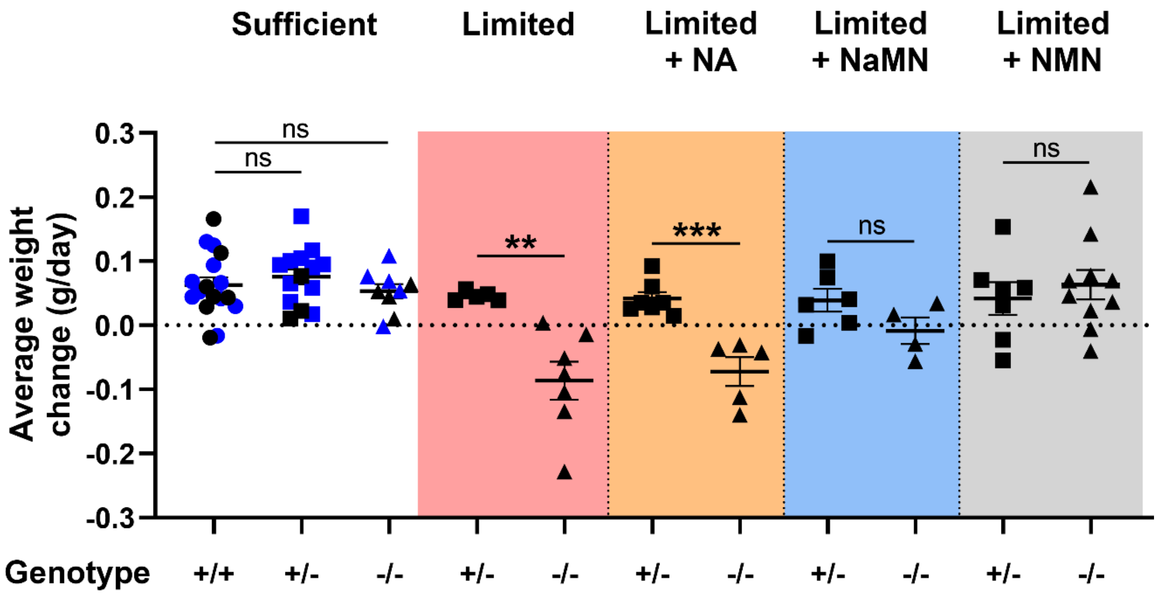

Supplemental Figure 8. Weight change in non-pregnant female *Nadsyn1+/-* and *Nadsyn1-/-* mice maintained on various dietary conditions for 17 days. Each data point represents the average weight difference of a mouse on the respective diet between day 0 and day 17, with the dotted line indicating 0 (no average weight change). Black points indicate mice from which liver (Figure 5A, Supplemental Table 9) and plasma (Figure 5, B, C, and D, Supplemental Figure 12) samples were subsequently assessed; blue points indicate mice from which no further tissue or blood samples were collected (see Supplemental Figure 7B). Statistical comparisons between *Nadsyn1*+/+, *Nadsyn1*+/-, and *Nadsyn1*-/- mice fed the *Sufficient Diet* were done by One-Way ANOVA. Statistical comparisons for the other dietary conditions were done by two-tailed paired Student's T-test because no *Nadsyn1*+/+ mice were given those diets. Bars indicate mean ± standard deviation. ***$p < 0.001$, **$p < 0.01$. ns: not significant; NA: nicotinic acid; NaMN: nicotinic acid mononucleotide; NMN: nicotinamide mononucleotide. $n \geq 4$ mice per *Nadsyn1* genotype per condition.

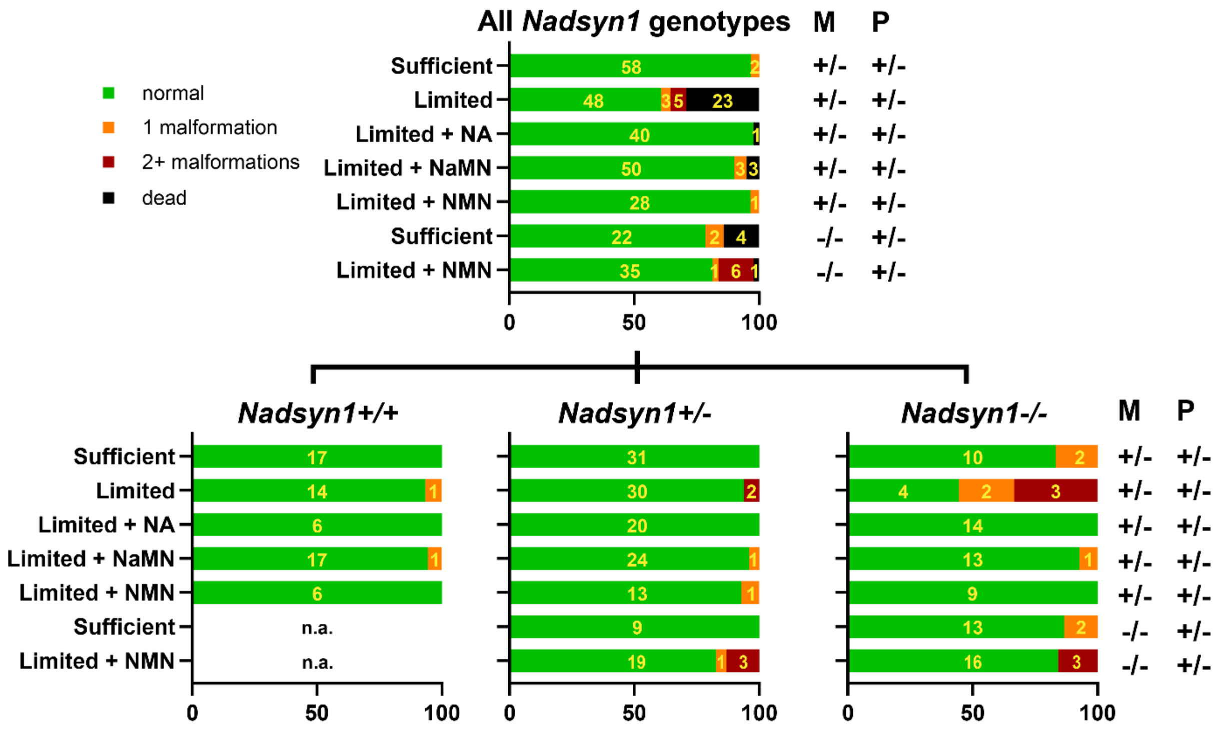

Supplemental Figure 9. Phenotypic outcomes of E17.5 embryos generated under various dietary conditions and mating schemes. The diets given to pregnant female mice are indicated on the left. The mating schemes are indicated on the right. *Nadsyn1*-/- mothers on *Limited Diet*, *Limited Diet* + NA, or *Limited Diet* + NaMN did not generate any embryos. M: maternal *Nadsyn1* genotype; P: paternal *Nadsyn1* genotype. NA: nicotinic acid; NaMN: nicotinic acid mononucleotide; NMN: nicotinamide mononucleotide; n.a.: not applicable because *Nadsyn1*-/- × *Nadsyn1*+/- matings cannot generate *Nadsyn1*+/+ embryos. Dead embryos could not be genotyped. Mouse numbers (n) are indicated within graphs in yellow. Information about the phenotypic outcomes of individual litters is presented in Supplemental Figure 10.

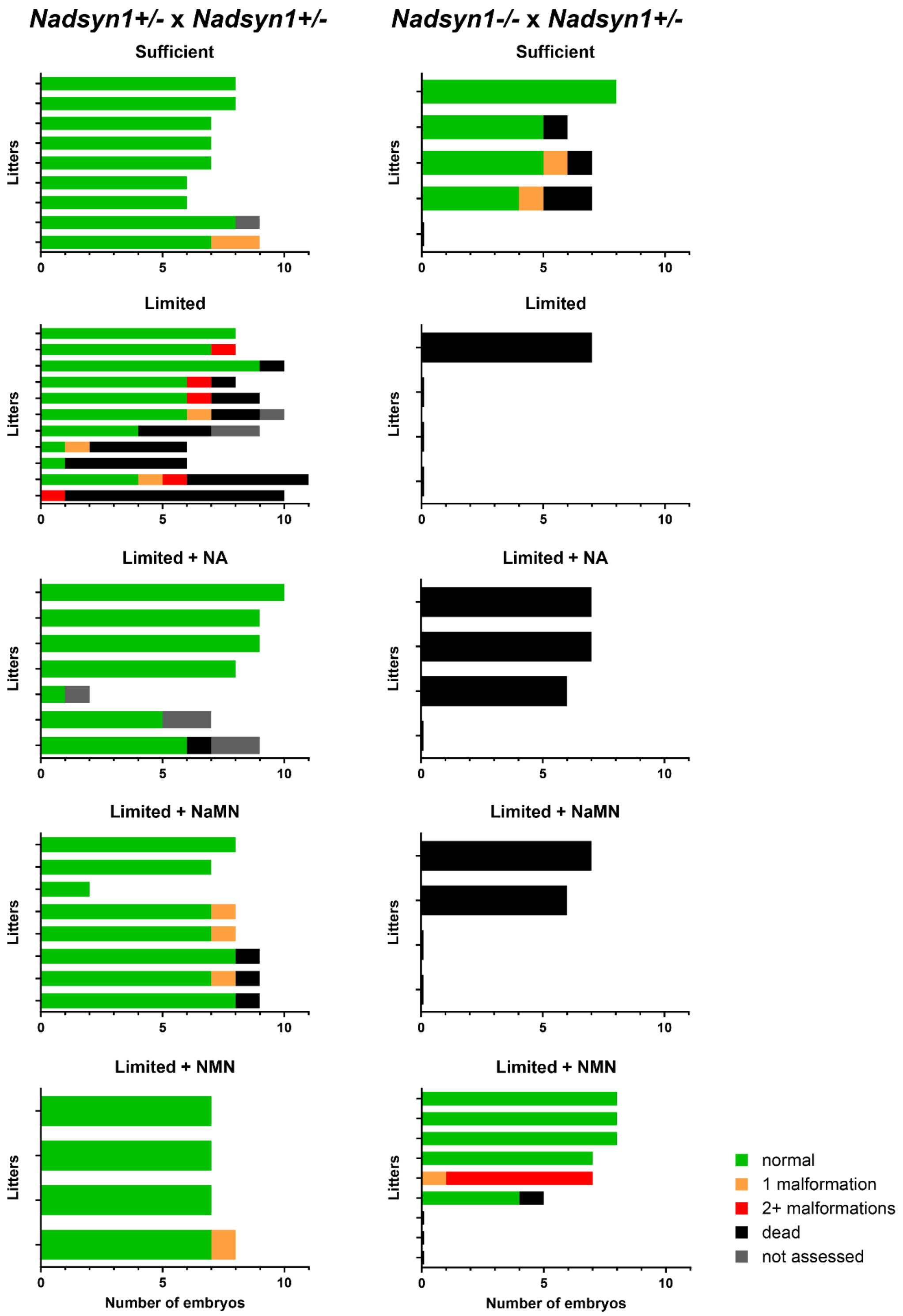

Supplemental Figure 10. Phenotypic outcomes of E17.5 embryo litters generated under various dietary conditions and mating schemes. Each bar represents a litter. The mating scheme (maternal × paternal) is indicated at the top. The diets given to the female mice throughout pregnancy are indicated above each graph. Subsets of *Nadsyn1*-/- mothers on *Limited Diet* or *Limited Diet* + any of the supplements did not generate any embryos (indicated by missing bars in the graphs). Some embryos could not be assessed for malformations (grey) due to sample loss during processing. NA: nicotinic acid; NaMN: nicotinic acid mononucleotide; NMN: nicotinamide mononucleotide

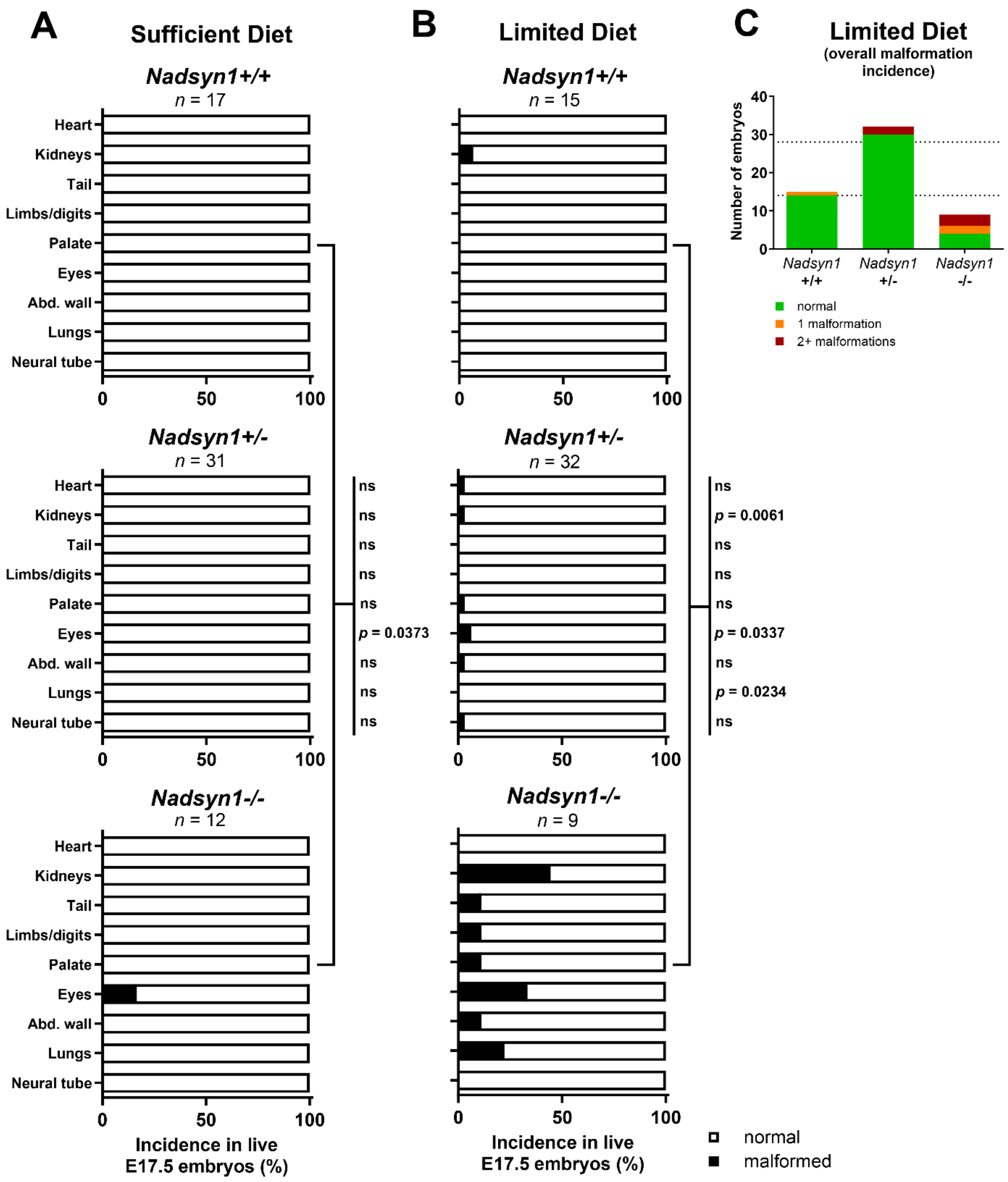

Supplemental Figure 11. Malformations found in E17.5 embryos generated from *Nadsyn1+/-* intercrosses with females given a *Sufficient Diet* or *Limited Diet* during pregnancy. The types of malformations observed in embryos of mothers on (**A**) *Sufficient Diet* and (**B**) *Limited Diet* are shown for each embryo *Nadsyn1* genotype (indicated above each graph). Statistical comparisons represent Fisher's exact test with Freeman-Halton extension for 3×2 contingencies. ns: not significant; Abd. wall: abdominal wall. (**C**) Overall incidence of isolated or multiple malformations in embryos from mothers on *Limited Diet*. Dotted lines indicate expected numbers of generated embryos by Mendelian ratios per *Nadsyn1* genotype. For details about observed malformations, see Supplemental Table 8.

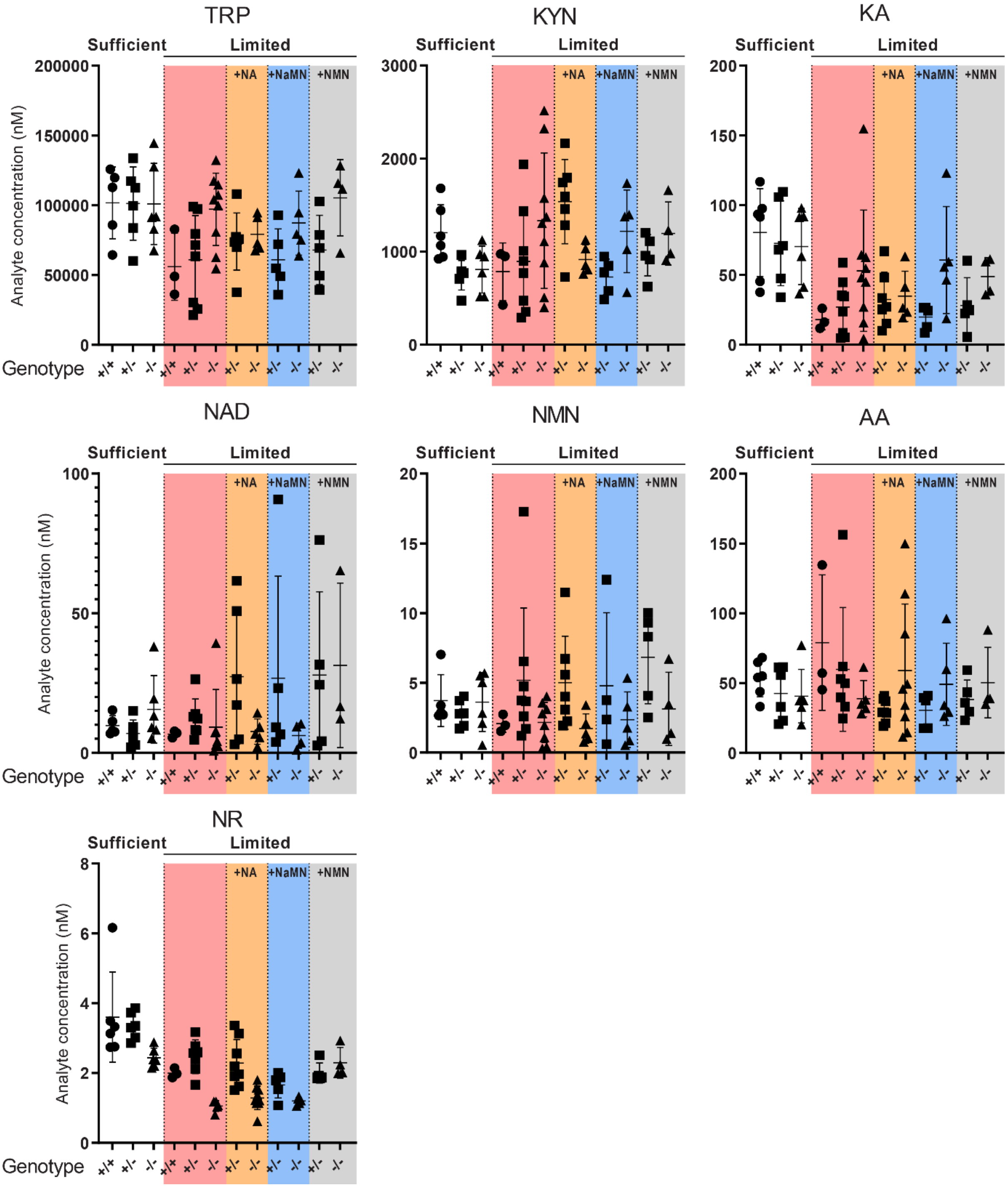

Supplemental Figure 12. Levels of NAD-related metabolites in plasma of female mice of different *Nadsyn1* genotypes under various dietary conditions. NAD-related metabolite concentrations were measured in plasma of mice fed various diets for 17 days. *Nadsyn1* genotypes are shown below each graph. Metabolite concentrations are provided in nM. Bars indicate mean ± standard deviation. Shown are metabolites without significant alterations among conditions, indicated by lower variable importance in projection (VIP) scores in a partial least squares discriminant analysis (PLS-DA) (see Figure 5, B, C, and D). TRP: tryptophan, KYN: kynurenine, KA: kynurenic acid, NAD+: nicotinamide adenine dinucleotide (oxidised), NMN: nicotinamide mononucleotide, AA: anthranilic acid, NR: nicotinamide riboside. $n \geq 3$ per *Nadsyn1* genotype per condition.

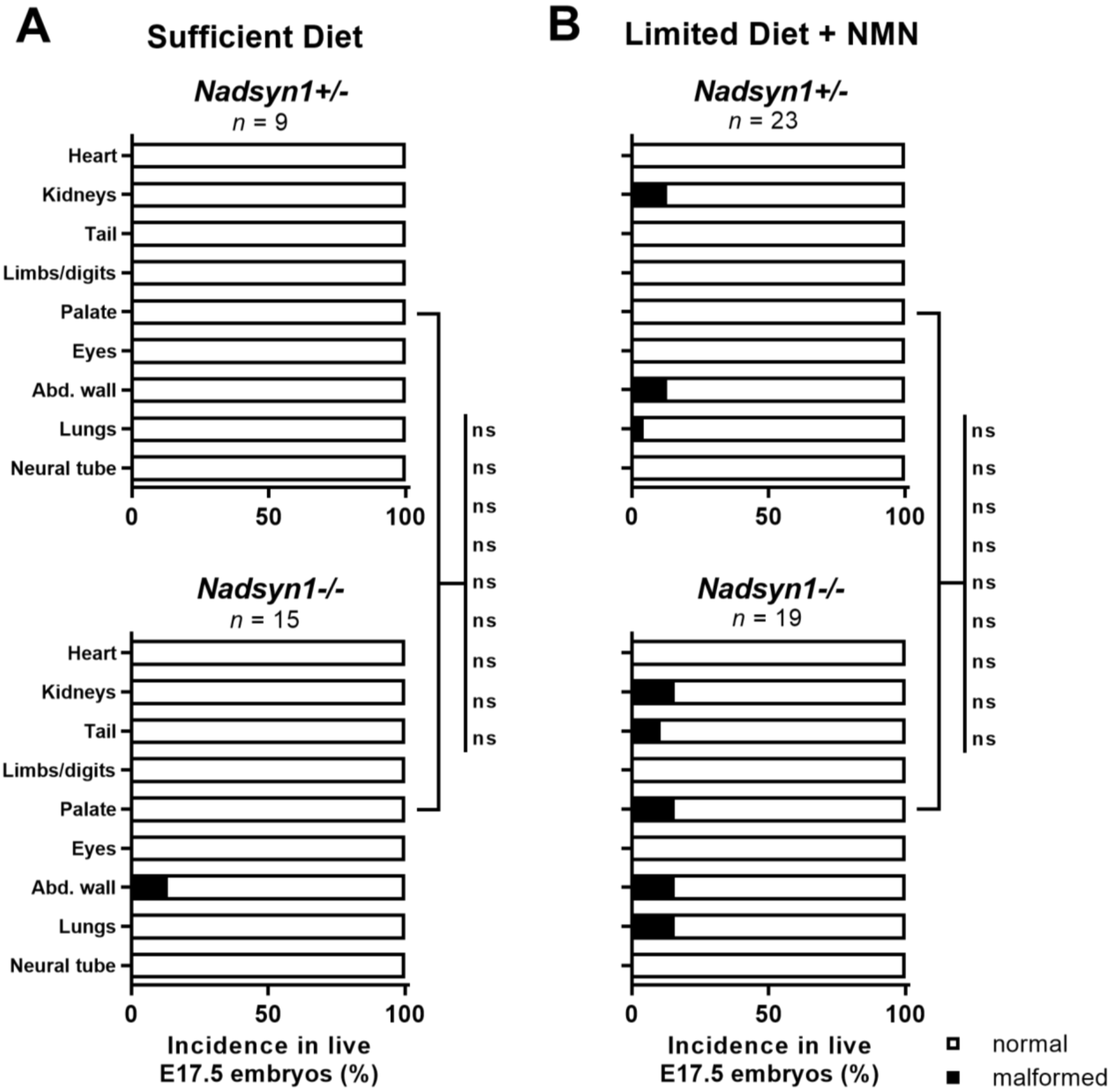

Supplemental Figure 13. Malformations found in E17.5 embryos generated from matings of *Nadsyn1-/-* females with *Nadsyn1+/-* males, and females given a *Sufficient Diet* or *Limited Diet* supplemented with NMN during pregnancy. The types of malformations observed in embryos of mothers on (**A**) *Sufficient Diet* and (**B**) *Limited Diet* + NMN are shown for each embryo *Nadsyn1* genotype (indicated above each graph). Statistical comparisons represent Fisher's exact test for 2×2 contingencies. NMN: nicotinamide mononucleotide; ns*:* not significant; Abd. wall: abdominal wall. For details about observed malformations, see Supplemental Table 8.

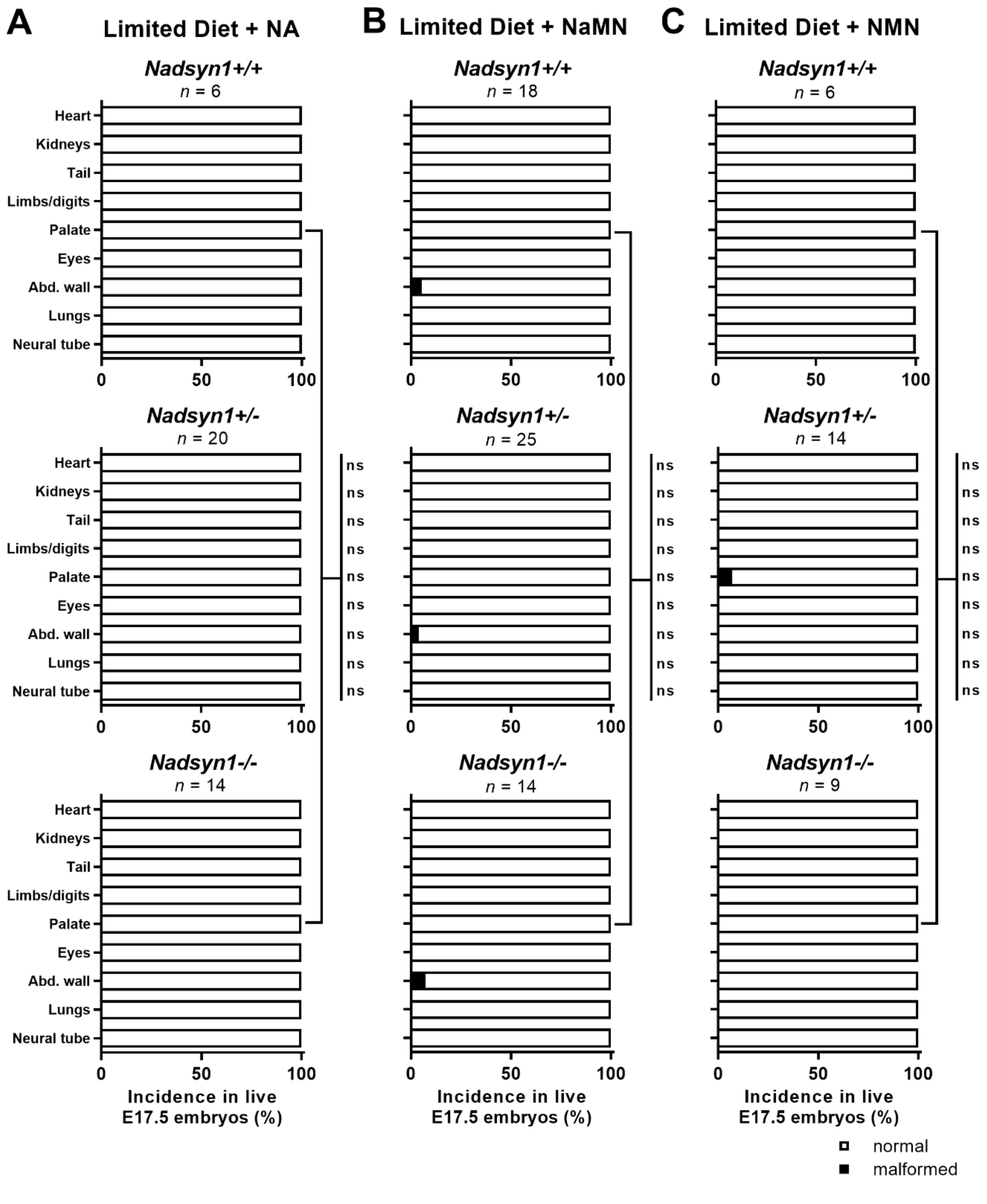

Supplemental Figure 14. Malformations found in E17.5 embryos generated from *Nadsyn1+/-* intercrosses with females given a *Limited Diet* with different supplements during pregnancy. The types of malformations observed in embryos of mothers on (**A**) *Limited Diet* + NA, (**B**) *Limited Diet* + NaMN, or (**C**) *Limited Diet* + NMN are shown for each embryo *Nadsyn1* genotype (indicated above each graph). Statistical comparisons represent Fisher's exact test with Freeman-Halton extension for 3×2 contingencies. NA: nicotinic acid; NaMN: nicotinic acid mononucleotide; NMN: nicotinamide mononucleotide; ns*:* not significant; Abd. wall: abdominal wall. For details about observed malformations, see Supplemental Table 8.

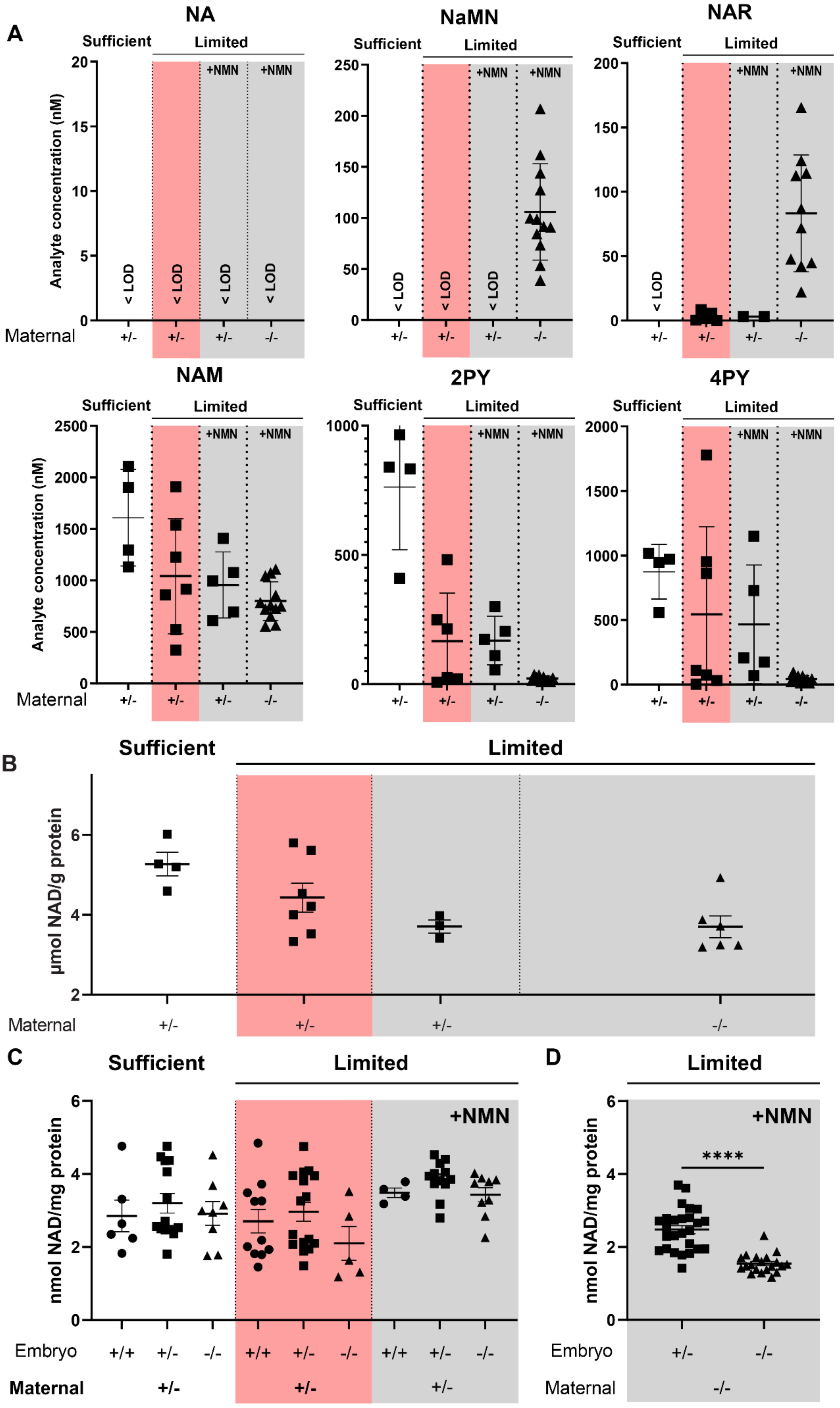
A
NA
NaMN
NAR
NAM
2PY
4PY
Sufficient
Limited
+NMN
< LOD
Analyte concentration (nM)
Maternal
+/-
-/-
B
Sufficient
Limited
µmol NAD/g protein
Maternal
C
Sufficient
Limited
+NMN
nmol NAD/mg protein
Embryo
+/+
+/-
-/-
Maternal
D
Limited
+NMN
****
nmol NAD/mg protein
Embryo
Maternal

Supplemental Figure 15. Pregnant mouse plasma NAD-related metabolites, maternal liver total NAD, and total NAD in generated E11.5 embryos. Male *Nadsyn1+/-* and female *Nadsyn1+/-* or *Nadsyn1-/-* mice were intercrossed and maternal plasma, liver, and generated embryos collected at E11.5. Assessed gestational diets included *Sufficient Diet*, *Limited Diet,* and *Limited Diet* + NMN. (**A**) Preiss-Handler and salvage pathway metabolites in pregnant maternal plasma. Maternal *Nadsyn1* genotypes are indicated below the graph. Bars indicate mean ± standard deviation. $n \geq 4$ per *Nadsyn1* genotype per condition; (**B**) Total NAD in pregnant maternal liver at E11.5. Dots represent NAD levels in individual liver samples from pregnant mice and bars indicate the mean ± standard deviation. $n \geq 4$ per *Nadsyn1* genotype per condition; (**C**) Total NAD in E11.5 embryos generated from *Nadsyn1+/-* mothers. Within-diet One-way ANOVA comparisons between embryo genotypes showed no significant differences. Bars indicate mean ± standard deviation. $n = 4 - 16$; (**D**) Total NAD in E11.5 embryos generated from *Nadsyn1-/-* mothers. Statistical comparison represents a two-tailed Student's T-test. Dots represent NAD levels in individual embryos and bars indicate the mean ± standard deviation. $n = 20 - 26$. Embryo and maternal *Nadsyn1* genotypes are indicated below respective graphs. Paternal genotype was *Nadsyn1*+/- in all matings. ****$p < 0.0001$, *$p < 0.05$. NMN: nicotinamide mononucleotide. For numerical values and statistical comparisons of NAD in E11.5 embryos, see Supplemental Table 12.

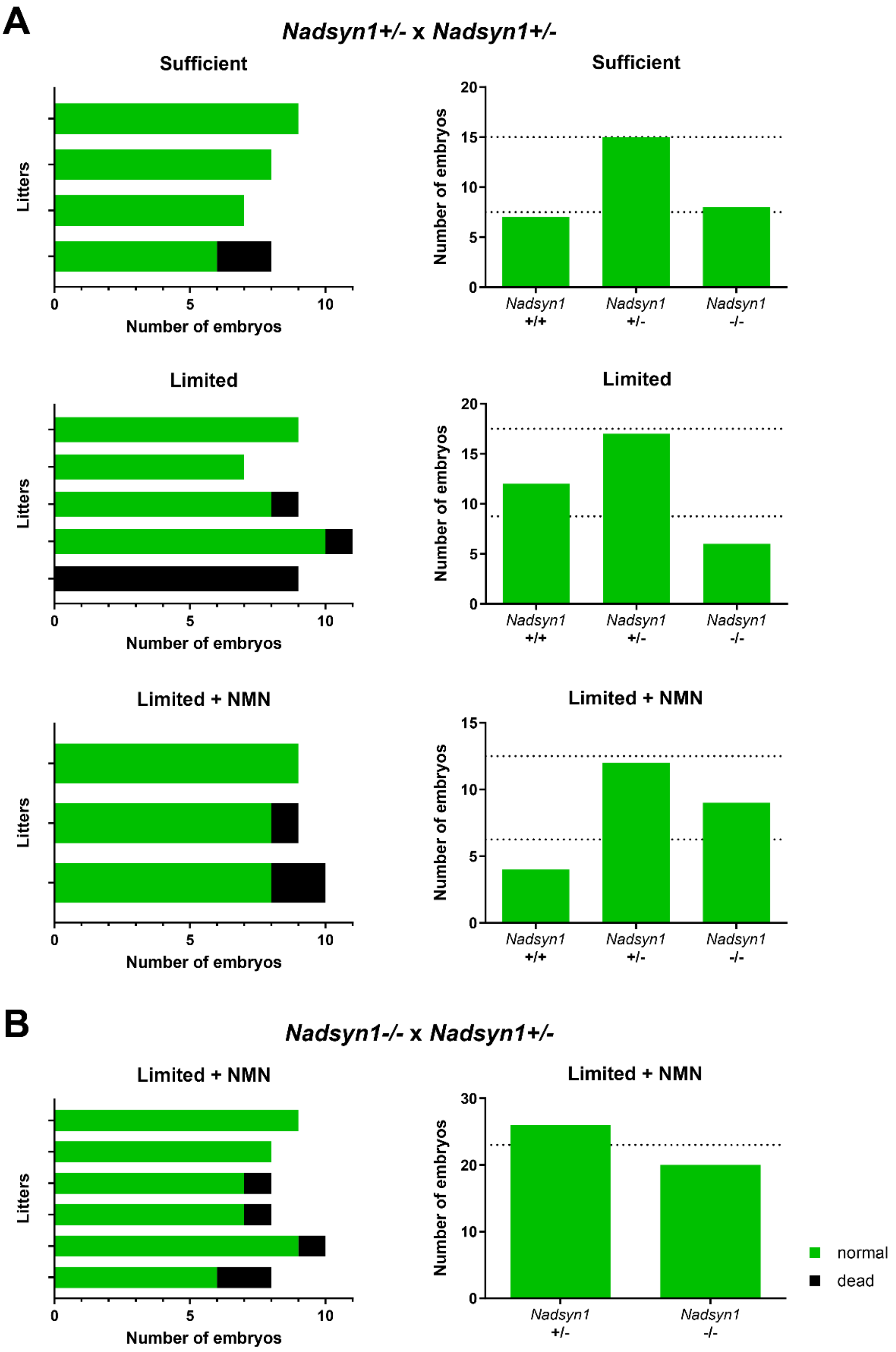

Supplemental Figure 16. Number of surviving and dead embryos at E11.5 under various dietary conditions and mating schemes. Numbers of live and dead E11.5 embryos from (**A**) *Nadsyn1*+/- intercrosses and (**B**) *Nadsyn1*-/- female x *Nadsyn1*+/- male matings are shown in the graphs on the left. Each bar represents a litter. Corresponding embryo *Nadsyn1*-genotype distributions are shown on the right. Dotted lines indicate expected numbers of embryos per genotype based on Mendelian ratios. Chi-Square test for expected Mendelian ratios of generated embryos was not statistically significant in any comparison.

Supplemental Tables

Supplemental Table 1. Expanded phenotypic description of patients with biallelic *NADSYN1* (NM_018161.5) variants

Patient [variant]	Sex	Vertebral	Cardiac	Renal	Limb	Other
F1.II.1 [c.379C>T p.R127C]; [c.379C>T p.R127C]	Male stillborn at 30 weeks	nd	Absent ductus venosus, single umbilical artery	-	Deep-set hypoplastic fingernails and toenails	**Neurodevelopmental:** nd; **CNS**: nd; **Microcephaly**: nd; **Growth:** nd; **Craniofacial**: absent nasal bone; **Dysmorphism**: short sloping forehead, deep set eyes, thin lips, retrognathia; **Vision**: nd; **Hearing:** nd; **Respiratory:** nd; **Skeletal:** nd; **Genitourinary:** nd; **Gastrointestinal:** nd; **Cutaneous:** nd; **Vascular/lymphatic:** Cystic hygroma, forehead and pedal skin edema, right jugular sac, two vessel cord
F2.II.3 [c.1717G>A p.A573T]; [c.1759G>A p.D587N]	Female	Abnormal vertebral morphology, abnormality of the ribs	Abnormal aortic arch morphology	-	-	**Neurodevelopmental:** -; **CNS:** Arnold-Chiari type I malformation; **Microcephaly:** Yes; **Growth:** -; **Craniofacial:** -; **Dysmorphism:** -; **Vision:** -; **Hearing:** Sensorineural hearing impairment; **Respiratory:** -; **Skeletal:** -; **Genitourinary:** -; **Gastrointestinal:** -; **Cutaneous:** -; **Vascular/lymphatic**: -
F3.II.1 [c.145T>C p.C49R]; [c.145T>C p.C49R]	Male	Hemi-vertebrae T8-L1	-	-	Hypoplastic toenails, overlapping toes	**Neurodevelopmental:** -; **CNS:** -; **Microcephaly:** -; **Growth:** short stature; **Craniofacial:** -; **Dysmorphism:** -; **Vision:** -; **Hearing:** -; **Respiratory:** -; **Skeletal:** -; **Genitourinary:** Cryptorchidism; **Gastrointestinal:** -; **Cutaneous:** -; **Vascular/lymphatic:** Inflammatory vascular lesions on scalp in newborn period
F4.II.2 [c.1717G>A p.A573T]; [c.1717G>A p.A573T]	Female	Kyphoscoliosis, 5 abnormal vertebrae (hemivertebrae, butterfly), spina bifida occulta	Coarctation of the aorta with ALCAPA, persistent LSVC	Positioned slightly lower than normal	Mild pes planus, joint hypermobility, short 4th left metacarpal, upturned nails	**Neurodevelopmental:** -; **CNS:** -; **Microcephaly:** -; **Growth:** short stature; **Craniofacial:** slightly high arched palate, delayed eruption of secondary teeth; **Dysmorphism:** -; **Vision:** myopia; **Hearing:** -; **Respiratory:** -; **Skeletal:** pectus excavatum, winged scapula; **Genitourinary:** voiding dysfunction and recurrent UTI; **Gastrointestinal:** GERD, constipation; **Cutaneous:** soft skin; **Vascular/lymphatic:** hemangioma over coccyx
F5.II.1 [c.1765-7T>A p.(?)]; [c.1088C>T p.A363V]	Male	-	Bicuspid aortic valve, coarctation of the aorta	-	Clinodactyly	**Neurodevelopmental:** muscular hypotonia, autism spectrum disorder (moderate); **CNS:** -; **Microcephaly:** -; **Growth:** -; **Craniofacial:** high arched palate; **Dysmorphism:** long flat philtrum, ptosis; **Vision:** -; **Hearing:** Bilateral sensorineural hearing loss (high frequencies); **Respiratory:** -; **Skeletal:** -;

Patient [variant]	Sex	Vertebral	Cardiac	Renal	Limb	Other
						**Genitourinary:** -; **Gastrointestinal:** -; **Cutaneous:** -; **Vascular/lymphatic:** -
F6.II.2 [c.524G>A p.C175Y]; [c.524G>A p.C175Y]	Female (deceased at 13 days)	Vertebral segmentation defects, rib abnormalities (10 pairs, partial fusion posterior ribs 6-7, 9-10 right side; 7-8 left side), hypoplastic sacrum	Tetralogy of Fallot, AVSD, OA, PS	-	Upper limb rhizomelia, left thumb appears triphalangeal and proximally inserted, fusion proximal L radius/ulna, short metacarpals, some hypoplastic distal phalanges	**Neurodevelopmental:** nd; **CNS:** large posterior fossa cyst (atypical Blake's pouch); **Microcephaly:** -; **Growth:** -; **Craniofacial:** High arched palate, bifid uvula; **Dysmorphism:** limited assessment of facial features as ventilator dependent until death: low set ears with thickened helices; **Vision:** unilateral microphthalmia; **Hearing:** nd; **Respiratory:** pulmonary hypoplasia; **Skeletal:** Deficient sternum, obtuse mandibular angle, diffuse sclerosis throughout, dense skull base, no discernible medullary cavities, hypoplastic left orbit (all likely due to osteopetrosis diagnosis); short broad neck; **Genitourinary:** hydrometrocolopos, single urogenital sinus; **Gastrointestinal:** -; **Cutaneous:** -; **Vascular/lymphatic**: -
F7.II.3 [c.524G>A p.C175Y]; [c.524G>A p.C175Y]	Male	Multiple thoracic vertebrae segmentation and formation defects (T8-T11), butterfly vertebrae (T8, T10-11), lower thoracic scoliosis, bilateral rudimentary 1st ribs, posterior fusion of right 8th-10th ribs	Double outlet right ventricle, hypoplastic left ventricle, hypoplastic mitral valve, coarctation of the aorta, subaortic ventricular septal defect	-	-	**Neurodevelopmental:** speech delay, behavior problems; **CNS:** delayed myelination, mild white matter volume loss; **Microcephaly:** Yes; **Growth:** Short stature; **Craniofacial:** -; **Dysmorphism:** Micrognathia; **Vision:** -; **Hearing:** -; **Respiratory:** -; **Skeletal:** -; **Genitourinary:** -; **Gastrointestinal:** -; **Cutaneous:** sacral dimple; **Vascular/lymphatic:** -
F8.II.1 [1717G>A p.A573T]; [1717G>A p.A573T]	Male (TOP 19 weeks)	Lower dorsal butterfly-like or hemivertebra, anomalous development of the 1st and 2nd ribs, L5 forme fruste butterfly vertebra	Hypoplastic left heart, small globular left ventricle, aortic valve stenosis, hypoplasia of the aortic arch,	-	-	**Neurodevelopmental:** nd; **CNS:** nd; **Microcephaly:** -; **Growth:** nd; **Craniofacial:** -; **Dysmorphism:** -; **Vision:** nd; **Hearing:** nd; **Respiratory:** -; **Skeletal:** -; **Genitourinary:** -; **Gastrointestinal:** -; **Cutaneous:** -; **Vascular/lymphatic:** -
F8.II.2 [1717G>A p.A573T]; [1717G>A p.A573T]	Female (TOP 18 weeks 5 days)	Multiple segmental abnormalities of the mid and lower dorsal vertebral bodies, reduced number of ribs on both sides, posterior fusion of left lower ribs, partial S1 and S2 fusions	Hypoplastic left heart, large right atrium and right ventricle, right ventricular outflow tract, TGA, double outlet right ventricle, small left ventricle,	-	Prominent sandal gap on the right foot	**Neurodevelopmental:** nd; **CNS:** nd; **Microcephaly:** -; **Growth:** nd; **Craniofacial:** -; **Dysmorphism:** -; **Vision:** nd; **Hearing:** nd; **Respiratory:** Unilobed right lung and bilobed left lung; **Skeletal:** -; **Genitourinary:** -; **Gastrointestinal:** -; **Cutaneous:** -; **Vascular/lymphatic:** -

Patient [variant]	Sex	Vertebral	Cardiac	Renal	Limb	Other
			hypoplasia of the aortic arch			
F8.II.3 [1717G>A p.A573T]; [1717G>A p.A573T]	Male	Subtle fusion of L4/L5 and wedged appearance of L1	-	-	Camptodactyly of left middle and ring finger	**Neurodevelopmental:** Mild gross motor delay; **CNS:** -; **Microcephaly:** -; **Growth:** short stature; **Craniofacial:** -; **Dysmorphism:** hypertelorism, low-set posterior rotated ears, hooded eyelids, downslanting palpebral fissures; **Vision:** -; **Hearing:** -; **Respiratory:** -; **Skeletal:** -; **Genitourinary:** -; **Gastrointestinal:** -; **Cutaneous:** -; **Vascular/lymphatic**: -
F9.II.4 [c.271del p.M91Cfs*11]; [c.271del p.M91Cfs*11]	Male	Mild scoliosis	-	Right renal pelviectasis SFU 1, improving after void, slightly enlarged right kidney, left renal hydronephrosis SFU 2	-	**Neurodevelopmental:** -; **CNS:** -; **Microcephaly:** -; **Growth:** -; **Craniofacial:** velopharyngeal insufficiency; **Dysmorphism:** brachycephaly, narrow palpebral fissures, full lips, large nose; **Vision:** -; **Hearing:** -; **Respiratory:** -; **Skeletal:** -; **Genitourinary:** - **Gastrointestinal:** -; **Cutaneous:** recurrent photosensitive rash, inverted nipples; **Vascular/lymphatic:** -
F10.II.2 [c.1717G>A p.A573T]; [c.1459C>T p.R487*]	Male	Abnormal vertebrae: 10th thoracic butterfly vertebrae, $11\text{-}12^{th}$ thoracic fusion and abnormal rib (possibly due to vertebral fusion)	AVSD	Small and dysplastic	Left arm and hand rhizomelia, thin thumb, biphalangeal 2nd digit; Right arm and hand rhizomelia, hypoplastic thumb, duplication of 2nd digit and duplication of the nails	**Neurodevelopmental:** hypotonia, developmental delay; **CNS:** -; **Microcephaly:** -; **Growth:** failure to thrive; **Craniofacial:** cleft palate; **Dysmorphism:** Pear-shaped face, full cheeks, hypertelorism, upslanting palpebral fissures, high nasal bridge, upturned nares, broad nasal tip, retrognathia, hypoplasia of the right ear, dysplastic left ear; **Vision:** -; **Hearing:** hearing loss; **Respiratory:** tracheomalacia; **Skeletal:** short neck; **Genitourinary:** glandular hypospadias; **Gastrointestinal:** -; **Cutaneous:** -; **Vascular/lymphatic:** -

-: Assessed but no abnormality found; nd: not determined; VSD: ventricular septal defect; ALCAPA: anomalous left coronary artery from the pulmonary artery; LSVC: left superior vena cava; AVSD: atrioventricular septal defect; OA: overriding aorta; PS: pulmonary stenosis; TGA: Transposition of the great arteries; GERD: gastro-esophageal reflux disease. Phenotypes previously not reported in CNDD patients are highlighted in red text.

Supplemental Table 2. Predicted impact of NADSYN1 missense mutation in silico to substrate binding affinities

Missense variant	Distance to glutamine (Å)	Δglutamine affinity (mCSM-lig)	Distance to ATP (Å)	ΔATP affinity (mCSM-lig)	Distance to NaAD (Å)	ΔNaAD affinity (mmCSM-lig)
C49R	11.83	-0.189	>12 Å	-	>12 Å	-
R127C	11.14	-0.386	>12 Å	-	>12 Å	-
C175Y	2.48	-0.209	>12 Å	-	>12 Å	-
A363V	>12 Å	-	5.25	-0.905	>12 Å	-
A573T	>12 Å	-	3.56	-0.945	3.17	-1.051
D587N	>12 Å	-	3.69	-0.644	5.44	-0.696

Changes in NADSYN1 monomer affinities to interacting partners as calculated on the complete Alphafold2 structures. Values obtained were grouped into mild (|0.05 – 0.5|), moderate (|0.5 – 1.0|), or large (|>1.0|), depending on their deviation from zero. Interaction distances >12 Å were considered insignificant.

Supplemental Table 3. Concentrations of NAD and related metabolites in patients with biallelic *NADSYN1* variants and their parents

	Patient	F3.I.1	F3.I.2	F3.II.3	F5.I.1	F5.I.2	F5.II.1	F7.I.1	F7.I.2	F7.II.3	+NAM	+2xNAM
	***NADSYN1* genotype (1)**	c.145T>C p.C49R	WT	c.145T>C p.C49R	c.1765-7T>A	WT	c.1765-7T>A	c.524G>A p.C175Y	WT	c.524G>A p.C175Y		
	**% of WT activity[A]**	0	100	0	0	100	0	0.62	100	0.62		
	***NADSYN1* genotype (2)**	WT	c.145T>C p.C49R	c.145T>C p.C49R	WT	c.1088C>T p.A363V	c.1088C>T p.A363V	WT	c.524G>A p.C175Y	c.524G>A p.C175Y		
	**% of WT activity[A]**	100	0	0	100	47.6	47.6	100	0.62	0.62		
	**Fasting status**	Yes	Yes	Yes	Yes	Yes	Yes	Yes	Yes	No[C]	Yes	Yes
	**Supplementation**	No	No	No	No	No	No	No	No	No	Yes	Yes
	**Age at assessment (years)**	42	32	1.6	44.6	45.5	~10	37	32	1.8	2.2	2.3
	**Whole Blood Metabolites (nM)**											
NAD de novo synthesis pathway	Tryptophan	51572	41195	44871	28815	29689	31889	32484	33839	38510	34927	34503
	Kynurenine	1610	1803	2104	1456	1356	1860	1620	1492	1860	1784	2202
	3-Hydroxykynurenine	<LOD	<LOD	<LOD	<LOD	<LOD	<LOD	<LOD	<LOD	<LOD	<LOD	<LOD
	3-Hydroxyanthranilic acid	33.4	23.8	32.0	19.5	21.3	21.3	18.5	21.2	12.1	23.4	<LOD
	Kynurenic acid	37.6	43.8	44.7	25.6	22.2	56.4	73.8	52.3	30.1	32.5	28.2
	Anthranilic acid	39.6	37.7	13.4	25.6	11.6	29.5	57.4	28.1	22.9	19.6	73.8
	Xanthurenic acid	16.5	15.1	13.0	13.5	14.1	16.8	21.6	15.0	11.1	11.2	41.5
	Quinolinic acid	<LOD	<LOD	<LOD	<LOD	<LOD	<LOD	<LOD	<LOD	<LOD	<LOD	<LOD
Preiss-Handler pathway	Nicotinic acid	<LOD	<LOD	35.1	<LOD	<LOD	<LOD	<LOD	<LOD	5.43	5.37	20.2
	Nicotinic acid riboside	30.1	5.54	>2500[B]	<LOD	8.45	75.0	6.18	32.9	1594	>2500[B]	1.73
	Nicotinic acid mononucleotide	17.2	0.343	460	2.03	1.32	2.67	8.89	9.08	206	91.9	322
	Nicotinic acid adenine dinucleotide	<LOD	<LOD	62669	<LOD	<LOD	68.7	<LOD	57.6	8746	2351	8816
Salvage pathway	Nicotinamide adenine dinucleotide	15453	9731	17911	19629	11000	21038	29313	19014	14490	28332	31154
	Nicotinamide	1770	1496	1497	1618	3840	2823	1747	3752	3080	1701	5501
	Nicotinamide mononucleotide	2102	1221	2359	2058	1459	2013	1611	1657	2585	2129	2353
	Nicotinamide riboside	0.763	<LOD	0.0635	0.0907	<LOD	0.0588	<LOD	<LOD	0.0324	<LOD	<LOD
Excretion products	1-Methylnicotinamide	61.3	98.7	20.3	98.2	104.7	87.0	132	126	78.2	170	768
	N1-Methyl-2-pyridone-5-carboxamide	511	728	164	1206	1137	1008	1434	2497	813	2750	11782
	N1-Methyl-4-pyridone-3-carboxamide	154	200	48.4	369	327	264	409	774	242	1037	4309
	**Plasma Metabolites (nM)**											
NAD de novo synthesis pathway	Tryptophan	66555	51533	55172	47140	36612	40791	56308	53556	51869	54858	54719
	Kynurenine	2613	2713	3544	1815	1259	2025	2295	2174	2549	2648	2297
	3-Hydroxykynurenine	36.2	43.6	57.2	35.6	35.4	38.1	39.0	53.0	70.1	65.0	72.1
	3-Hydroxyanthranilic acid	77.3	49.9	51.4	41.0	35.8	39.7	47.2	50.7	56.2	47.3	55.8
	Kynurenic acid	81.2	83.7	62.1	50.1	41.8	112	90.6	61.6	37.2	45.5	37.4
	Anthranilic acid	7.04	18.7	15.6	15.5	12.3	15.4	10.3	10.1	16.3	14.9	18.6
	Xanthurenic acid	25.3	22.2	16.5	18.4	17.0	24.0	34.2	23.0	16.1	14.0	15.7
	Quinolinic acid	<LOD	<LOD	<LOD	<LOD	<LOD	<LOD	<LOD	<LOD	<LOD	<LOD	<LOD
Preiss-Handler pathway	Nicotinic acid	<LOD	<LOD	6.78	<LOD	<LOD	<LOD	<LOD	<LOD	3.71	<LOD	<LOD
	Nicotinic acid riboside	<LOD	<LOD	>40000[B]	<LOD	<LOD	<LOD	33.4	39.9	>10000[B]	860	625
	Nicotinic acid mononucleotide	<LOD	<LOD	9.92	<LOD	<LOD	<LOD	<LOD	<LOD	0.918	0.334	2.87
	Nicotinic acid adenine dinucleotide	<LOD	<LOD	8.79	<LOD	<LOD	<LOD	<LOD	<LOD	3.42	<LOD	4.21
Salvage pathway	Nicotinamide adenine dinucleotide	5.71	8.54	12.6	10.8	9.79	15.8	11.6	25.8	34.7	14.9	2.11
	Nicotinamide	76.1	162	114	81.8	67.7	191	336	211	198	431	243
	Nicotinamide mononucleotide	2.86	1.32	6.33	4.52	3.87	8.1	8.13	9.94	15.4	8.56	0.178
	Nicotinamide riboside	0.230	0.245	0.370	0.458	0.482	0.395	0.309	0.472	0.559	0.646	0.555
Excretion products	1-Methylnicotinamide	32.9	111	19.7	164	96.2	143	162	126	84.8	183	622
	N1-Methyl-2-pyridone-5-carboxamide	866	1109	259	1861	1639	1454	2365	3213	1296	4257	11386
	N1-Methyl-4-pyridone-3-carboxamide	172	231	49.1	431	387	294	551	908	316	1477	4760

[A]Mutant protein activity represents NAD synthesized by canonical glutamine-dependent activity relative to wild-type activity in vitro (see Figure 3). [B]Above the limit of quantification, the value represents the upper quantification limit. [C]Anecdotally recorded to have consumed strawberries, crackers, quinoa chips, and ~1.3 mg nicotinamide via "Kid Essentials Nutritionally Complete 850 g (Nestlé)" prior to sample collection. <LOD: below limit of detection (signal:noise < 3).

Supplemental Table 4. Plasma Global Metabolomic Assisted Pathway Screen of F4.II.2

Analyte	z-score	Superpathway	Subpathway	HMDB ID
N6,N6,N6-trimethyllysine	4.1	Amino acid	Lysine Metabolism	HMBD0001325
isourodeoxycholate	2.8	Lipid	Secondary Bile Acid Metabolism	HMBD0000686
guinidinoacetate	2.7	Amino acid	Creatine Metabolism	HMBD0000128
2-hydroxy-3-methylvalerate	2.6	Amino acid	Leucine, Isoleucine and Valine Metabolism	HMBD0000317
ursodeoxycholate	2.6	Lipid	Secondary Bile Acid Metabolism	HMBD0000946
glutarate (C5-DC)	2.5	Lipid	Fatty Acid, Dicarboxylate	HMBD0000661
1-eicosapentaenoyl-GPE (20:5)	2.5	Lipid	Lysophospholipid	HMBD0011489
1-eicosapentaenoyl-GPC (20:5)	2.4	Lipid	Lysophospholipid	HMBD0010397
3-(4-hydroxyphenyl)lactate	2.4	Amino acid	Tyrosine Metabolism	HMBD0000755
3-carboxy-4-methyl-5-pentyl-furanproprionate (3-CMPFP)	2.4	Lipid	Fatty Acid, Dicarboxylate	HMBD0061643
alpha-hydroxyisocaproate	2.4	Amino acid	Leucine, Isoleucine and Valine Metabolism	HMBD0000665, HMDB00746
1-(1-enyl-stearoyl)-2-docosapentaenoyl-GPE (P-18:0/22:5n3)	2.3	Lipid	Plasmalogen	HMBD0011393
palmitoylcholine	2.3	Lipid	Fatty Acid Metabolism (Acyl Choline)	HMBD0240592
trimethylamine N-oxide	2.3	Lipid	Phospholipid Metabolism	HMBD0000925
phosphatidylcholine (18:0/20:5, 16:0/22:5n6)	2.3	Lipid	Phosphatidylcholine (PC)	HMBD0008050, HMDB0007989
1-(1-enyl-stearoyl)-2-docosahexaenoyl-GPE (P-18:0/22:5n3)	2.2	Lipid	Plasmalogen	HMBD0011394
androstenediol (3beta, 17beta) monosulfate (2)	2.2	Lipid	Androgenic Steroids	HMBD0240429, HMDB0186954
1-docosahexaenoyl-GPE (22:6)	2.2	Lipid	Lysophospholipid	HMBD11496
creatinine	2	Amino acid	Creatine Metabolism	HMBD0000562
etiocholanolone glucuronide	2.2	Lipid	Androgenic Steroids	HMBD0004484
picolinate	2.1	Amino acid	Tryptophan Metabolism	HMBD0002243
5alpha-androstan-3beta, 17-beta-diol monosulfate (1)	2.1	Lipid	Androgenic Steroids	NA

androsterone glucuronide	2.1	Lipid	Androgenic Steroids	HMBD0002829
5alpha-androstan-3alpha, 17-beta-diol monosulfate (1)	2	Lipid	Androgenic Steroids	NA
homoarginine	2	Amino acid	Urea cycle; Arginine and Proline Metabolism	HMBD0000670
threonine	2	Amino acid	Glycine, Serine and Threonine Metabolism	HMBD0000167
glycolithocholate sulfate	-2.3	Lipid	Secondary Bile Acid Metabolism	HMBD0002639
taurolithocholate 3-sulfate	-2.4	Lipid	Secondary Bile Acid Metabolism	HMBD0002580
pro-hydroxy-pro	-2.5	Amino acid	Urea cycle; Arginine and Proline Metabolism	HMBD0006695
glycerate	-2.7	Carbohydrate	Glycolysis, Gluconeogenesis, and Pyruvate Metabolism	HMBD0000139, HMDB0006372
N-acetylaspartate (NAA)	-2.9	Amino acid	Alanine and Aspartate Metabolism	HMBD0000812
threonate	-2.9	Cofactors and Vitamins	Ascorbate and Aldarate Metabolism	HMBD0062620, HMDB0000943
oxalate (ethanedioate)	-3.2	Cofactors and Vitamins	Ascorbate and Aldarate Metabolism	HMBD0002329
Rare analytes present in sample (found in <5% of all previous specimen) and possibly related to the patient's phenotype				
**Analyte**	**Prevalence**	**Superpathway**	**Subpathway**	**HMDB ID**
indoleacetoylcarnitine	0.1	Amino acid	Tryptophan Metabolism	NA
indoleacetylglutamine	0.1	Amino acid	Tryptophan Metabolism	HMBD0013240
lamtrigine	0.1	Amino acid	Drug - Neurological	HMBD0014695
Common analytes (found in >99% of all previous specimen) ABSENT in this sample and possibly related to the patient's phenotype				
None detected.				
Significantly altered analytes likely related to nutrition, diet, supplements, or medication				
**Analyte**	**z-score**	**Superpathway**	**Subpathway**	**HMDB ID**
guaiacol sulfate	2.8	Xenobiotics	Benzoate Metabolism	HMBD0060013
catechol sulfate	2.7	Xenobiotics	Benzoate Metabolism	HMBD0059724

perfluorooctanesulfonate (PFOS)	2.1	Xenobiotics	Chemical	HMDB0059586
tartronate (hydroxymalonate)	-3.3	Xenobiotics	Food Component/Plant	HMDB0035227
NAD-related metabolites detectable by untargeted screen				
**Analyte**	**z-score**	**Superpathway**	**Subpathway**	**HMDB ID**
quinolinate	-0.8	Organoheterocyclic compounds	Nicotinate and nicotinamide metabolism	HMDB0000232
nicotinamide	-0.01	Organoheterocyclic compounds	Nicotinate and nicotinamide metabolism	HMDB0001406
ADP-ribose	0.6	Nucleosides, nucleotides, and analogues	Nicotinate and nicotinamide metabolism	HMDB0001178
N1-methyl-2-pyridone-5-carboxamide	-0.75	Organoheterocyclic compounds	Nicotinate and nicotinamide metabolism	HMDB0004193
trigonelline	0.2	Alkaloids and derivatives	Unknown	HMDB0000875
1-methylnicotinamide	-1.83	Organoheterocyclic compounds	Nicotinate and nicotinamide metabolism	HMDB0000699
kynurenine	0.6	Organic oxygen compounds	Tryptophan Metabolism	HMDB0000684
kynurenate	0.3	Organoheterocyclic compounds	Tryptophan Metabolism	HMDB0000715
N-formylanthranilic acid	0.96	Benzenoids	Tryptophan Metabolism	HMDB0004089

3-hydroxykynurenine and nicotinic acid adenine dinucleotide were not detectable via this assessment. z-scores are calculated using mean and standard deviation from healthy populations. HMDB = human metabolome database http://www.hmdb.ca/. Global Metabolomic Assisted Pathway Screen was performed by Baylor Genetics. NAD synthesis pathway-related metabolites are highlighted in red.

Supplemental Table 5. Urinary NAD metabolome components in Family 7 prior to and post-NAM supplementation

	Patient	F7.I.1	F7.I.2	F7.II.3[A]	F7.II.3[B]	+NAM
	***NADSYN1* genotype (1)**	c.524G>A p.C175Y	WT	c.524G>A p.C175Y		
	**% of WT activity[C]**	0.62	100	0.62		
	***NADSYN1* genotype (2)**	WT	c.524G>A p.C175Y	c.524G>A p.C175Y		
	**% of WT activity[C]**	100	0.62	0.62		
	**Fasting status**	Yes	Yes	No	No	Yes
	**Supplementation**	No	No	No	No	Yes
	**Age at assessment (years)**	37	32	1.7	1.7	2.2
	**Urine metabolite levels (µM) normalized to creatinine (mM)**					
NAD *de novo* synthesis pathway	Kynurenine	0.616	1.06	2.15	0.766	1.36
	3-Hydroxykynurenine	0.255	0.382	0.0887	0.198	0.277
	3-Hydroxyanthranilic acid	0.741	1.15	0.763	2.58	1.01
	Kynurenic acid	2.91	2.99	1.73	2.96	4
	Anthranilic acid	0.06	<LOD	0.225	<LOD	<LOD
	Xanthurenic acid	0.584	0.542	0.778	0.811	0.537
	Quinolinic acid	0.757	1.2	9.59	4.15	5.4
Preiss-Handler pathway	Nicotinic acid	<LOD	0.0071	0.153	0.0975	<LOD
	Nicotinic acid riboside	<LOD	0.126	66.7	46.2	42.6
	Nicotinic acid mononucleotide	<LOD	<LOD	0.348	0.339	0.28
	Nicotinic acid adenine dinucleotide	<LOD	<LOD	<LOD	<LOD	<LOD
Salvage pathway	Nicotinamide adenine dinucleotide	<LOD	<LOD	<LOD	<LOD	<LOD
	Nicotinamide	0.284	0.206	0.378	0.0746	0.251
	Nicotinamide mononucleotide	0.0139	0.0305	0.464	0.339	0.438
	Nicotinamide riboside	<LOD	<LOD	<LOD	<LOD	<LOD
Excretion products	1-Methylnicotinamide	8.76	15.5	11.1	5.54	39.1
	N1-Methyl-2-pyridone-5-carboxamide	22.2	>52.9[D]	81	32.4	>151[D]
	N1-Methyl-4-pyridone-3-carboxamide	5.83	25.3	19.4	7.16	112

[A]First and [B]second non-fasting urine collection from F9.II.3. [C]Mutant protein activity represents NAD synthesized by canonical glutamine-dependent activity relative to wild-type activity in vitro (see Figure 3). [D]Above the limit of quantification, the value represents the upper quantification limit. <LOD: below limit of detection (signal:noise < 3). WT: wild-type.

Supplemental Table 6. Mouse dietary treatments, their NAD precursor contributions, and how they compare to the human recommended dietary intake

Diet	Tryptophan in feed (mg/kg)	NA in feed (mg/kg)	NAM in feed (mg/kg)	Tryptophan in water (mg/kg)	Non-tryptophan NAD precursor in water (reported as 'mg niacin/kg')	Total NAD precursors (µg/d)	% RDI for human pregnancy
Breeder	3700	90		0	0	592	667.91
Standard	2700	31.4	0	0	0	298	336.49
Sufficient	2700	0.5	29.5	0	0	292.5	330.28
Limited[A]	0	0	1.4	600	0	67.46	76.17
Limited + NA	0	0	1.4	600	15	160.46	181.19
Limited + NaMN	0	0	1.4	600	15	160.46	181.19
Limited + NMN	0	0	1.4	600	15	160.46	181.19

NA: nicotinic acid; NAM: nicotinamide; NaMN: nicotinic acid mononucleotide; NMN: nicotinamide mononucleotide; RDI: recommended dietary intake. Drinking and food consumption rates in mice are calculated as previously reported (13) with 60 mg tryptophan assumed to be equivalent to 1 mg NA or 1 mg NAM for the conversion to NAD. Human equivalent doses of NAD precursors were calculated using the approximation that the mouse dose is 12.3-times the human dose (18). The RDI for NA in human pregnancy is 18 mg/day (19, 20). [A]This diet is identical to “NTF+TW600” (13).

Supplemental Table 7. Incidence of embryo loss and malformation in E18.5 embryos generated on the *Standard* and *Limited Diets*

Diet		Standard			Limited		
**Maternal genotype**		+/-			+/-		
**Paternal genotype**		+/-			+/-		
**Embryo genotype**		+/+	+/-	-/-	+/+	+/-	-/-
**Normal**		6 (86%)	17 (94%)	7 (100%)	26 (72%)	47 (71%)	12 (34%)
**1 malformation**		0 (0%)	1 (6%)	0 (0%)	3 (8%)	9 (14%)	8 (22%)
**2+ malformations**		1 (14%)	0 (0%)	0 (0%)	7 (19%)	10 (15%)	15 (43%)
**Death**		0 (0%)			37/178 (21%)		
**Specific malformations**							
**Heart**[A]	Bicuspid aortic valve	0	0	0	0	1	1
	Ventricular septal defect	0	0	0	2	2	3
	Persistent truncus arteriosus	0	0	0	1	0	6
	Double outlet right ventricle	0	0	0	0	0	1
	Overriding aorta	0	0	0	0	1	0
	Transposition of the great arteries	0	0	0	1	0	0
	Total	0/7 (0%)	0/18 (0%)	0/7 (0%)	3/36 (8%)	3/66 (5%)	9/35 (26%)
	Difference between groups	*ns*			***p* = 0.00576917		
**Neural tube**[B]	Exencephaly	0	0	0	0	0	1
	Spina bifida	0	0	0	0	0	1
	Total	0/7 (0%)	0/18 (0%)	0/7 (0%)	0/36 (0%)	0/66 (0%)	1/35 (3%)
	Difference between groups	*ns*			*ns*		
**Kidneys**[C]	Agenesis	0	0	0	0	3	12
	Hypoplasia	0	0	0	4	6	5
	Dysmorphic (duplex, hydronephrosis)	0	1	0	3	1	3
	Total	0/7 (0%)	1/18 (6%)	0/7 (0%)	7/36 (19%)	9/66 (14%)	19/35 (54%)
	Difference between groups	*ns*			*****p* < 0.0001		
**Lungs**[D]	Aplasia	0	0	0	3	2	11
	Hypoplasia	0	0	0	0	0	3
	Total	0/7 (0%)	0/18 (0%)	0/7 (0%)	3/36 (8%)	2/66 (3%)	12/35 (34%)
	Difference between groups	*ns*			*****p* < 0.0001		
**Tail**[E]	Short	0	0	0	4	7	15

	Curly	0	0	0	2	6	14
	Absent	0	0	0	0	0	1
	Total	0/7 (0%)	0/18 (0%)	0/7 (0%)	4/36 (11%)	8/66 (12%)	17/35 (49%)
	Difference between groups		*ns*			****$p < 0.0001$	
**Digits/Limbs**[F]	Polydactyly	0	0	0	1	2	2
	Oligodactyly	0	0	0	0	0	6
	Syndactyly	0	0	0	0	0	2
	Polysyndactyly	0	0	0	1	6	3
	Brachydactyly	0	0	0	0	0	3
	Talipes	0	0	0	1	2	6
	Short limbs	0	0	0	0	1	2
	Total	0/7 (0%)	0/18 (0%)	0/7 (0%)	3/36 (8%)	8/66 (12%)	11/35 (31%)
	Difference between groups		*ns*			*$p = 0.02103991$	
**Palate**[G]	Cleft palate	1	0	0	3	5	10
	Choanal atresia	0	0	0	1	1	1
	Microretrognathia	0	0	0	0	0	1
	Total	1/7 (14%)	0/18 (0%)	0/7 (0%)	3/36 (8%)	6/66 (9%)	10/35 (29%)
	Difference between groups		*ns*			*$p = 0.02350342$	
**Eyes**[H]	Anophthalmia	1	0	0	3	4	2
	Microphthalmia	0	0	0	3	10	6
	Total	1/7 (14%)	0/18 (0%)	0/7 (0%)	5/36 (14%)	13/66 (20%)	8/35 (23%)
	Difference between groups		*ns*			*ns*	
**Abdominal wall**[I]	Omphalocele	0	0	0	0	1	7
	Umbilical hernia	0	0	0	0	0	0
	Total	0/7 (0%)	0/18 (0%)	0/7 (0%)	0/36 (0%)	1/66 (2%)	7/35 (20%)
	Difference between groups		*ns*			***$p = 0.00041286$	
**Vertebrae**[J]	Scoliosis	0	0	0	6	2	8
	Butterfly vertebrae	0	0	0	0	0	2
	Caudal agenesis	0	0	0	0	0	5
	Sacral vertebral defect	0	0	0	0	0	1
	Cervical vertebrae fusion	0	0	0	1	0	0
	Lumbosacral defect	0	0	0	0	1	0
	Total	0/7 (0%)	0/18 (0%)	0/7 (0%)	7/36 (20%)	3/66 (5%)	14/35 (40%)
	Difference between groups		*ns*			****$p < 0.0001$	

[A]Heart defects assessed include: Bicuspid aortic valve, ventricular septal defect, overriding aorta, persistent truncus arteriosus, double outlet right ventricle, and transposition of the great arteries.
[B]Neural tube defects assessed include: Exencephaly, spina bifida.
[C]Kidney defects assessed include: Kidney hypoplasia (defined as having a tip-to-tip length ≤ 1.5 mm, as established in (13)), agenesis, duplex kidney, and hydronephrosis.
[D]Lung defects assessed include: Less than normal lobe number of 5 (aplasia) and underdeveloped parenchyma (hypoplasia).
[E]Tail defect defined as: Tails shorter than the length of the lower extremities (tail does not pass the toes of the embryo); 'curly' includes if the tail is kinked or reversed in direction.
[F]Digit and limb defects assessed include: Polydactyly, oligodactyly, syndactyly, polysyndactyly, brachydactyly, talipes, and short limbs.
[G]Palate defect classified as incomplete closure of the palatal shelf (cleft palate) or abnormality of the nasal cavity (choanal atresia).
[H]Eye defects assessed include: Anophthalmia and microphthalmia affecting one or both eyes.
[I]Abdominal wall defects assessed include: Omphalocele and umbilical hernia.
[J]Vertebral defects include: Scoliosis, butterfly vertebrae, caudal agenesis, sacral vertebral defects, cervical defects, and lumbosacral defects.
Totals indicate overall number of affected embryos per defect category. Statistical comparisons reflect 2×3 Fisher's exact tests with Freeman-Halton extension comparing incidence of defects versus no defects between *Nadsyn1* embryo genotypes per defect category. *$p < 0.05$; **$p < 0.01$; ***$p < 0.001$; ****$p < 0.0001$.

Supplemental Table 8. Summary of types and incidence of congenital malformations in E17.5 mouse embryos

	Diet	Sufficient	Sufficient	Limited	Limited + NA	Limited + NaMN	Limited + NMN	Limited + NMN
	Maternal genotype	+/-	-/-	+/-				-/-
	Paternal genotype	+/-		+/-				
Malformations								
Heart	Bicuspid aortic valve	0	0	1	0	0	0	0
	Right aortic arch	0	0	1	0	0	0	0
	Total	0/66	0/24	2/56	0/56	0/58	0/29	0/42
Neural tube	Exencephaly	0	0	1	0	0	0	0
	Total	0/66	0/24	1/56	0/56	0/58	0/29	0/42
Kidneys	Agenesis	0	0	0	0	0	0	1
	Hypoplasia[A]	0	0	6	0	0	0	5
	Dysmorphic (duplex, hydronephrosis)	0	0	1	0	0	0	0
	Total	0/66	0/24	7/56	0/56	0/58	0/29	6/42
Lungs	Aplasia	0	0	0	0	0	0	3
	Hypoplasia	0	0	1	0	0	0	4
	Total	0/66	0/24	1/56	0/56	0/58	0/29	4/42
Tail	Short	0	0	1	0	0	0	2
	Total	0/66	0/24	1/56	0/56	0/58	0/29	2/42
Palate	Cleft palate	0	0	2	0	0	1	3
	Total	0/66	0/24	2/56	0/56	0/58	1/29	3/42
Eyes	Anophthalmia	0	0	1	0	0	0	0
	Microphthalmia	2	0	4	0	0	0	0
	Total	2/66	0/24	5/56	0/56	0/58	0/29	0/42
Abdominal wall	Omphalocele or umbilical hernia	0	2	2	0	3	0	6
	Total	0/66	2/24	2/56	0/56	3/58	0/29	6/42

Assessed defects were as described in Supplemental Table 7 with the exception of the threshold established for kidney hypoplasia[A]: On the *Sufficient Diet*, across all embryo genotypes, the average kidney length was ~2.6 mm; hypoplastic kidneys were classified as having a length <1.3 mm.

Supplemental Table 9. Liver total NAD levels of female mice of different *Nadsyn1* genotypes under various dietary conditions.

Genotype	Diet + Supplement	NAD (μmol/g protein ± s.d.)	*n*	*p*
+/+	Sufficient	6.21 ± 0.745	6	0.0346
+/-	Sufficient	5.60 ± 0.978	7	
-/-	Sufficient	4.67 ± 0.354	7	
+/+	Limited	5.34 ± 0.513	5	0.0042
+/-	Limited	4.77 ± 0.991	9	
-/-	Limited	3.43 ± 0.782	10	
+/-	Limited + NA	4.90 ± 2.09	7	0.3020
-/-	Limited + NA	3.86 ± 0.368	5	
+/-	Limited + NaMN	5.54 ± 1.48	5	0.0319
-/-	Limited + NaMN	3.66 ± 0.653	5	
+/-	Limited + NMN	4.52 ± 1.08	5	0.8383
-/-	Limited + NMN	4.69 ± 1.44	5	

p values represent statistical comparisons between the *Nadsyn1* mouse genotypes of the respective dietary conditions and were done by one-way ANOVA for data with three genotypes (*Sufficient Diet* and *Limited Diet*) or by a two-tailed Student's t-test for the dietary conditions tested with mice of 2 different genotypes. NA: nicotinic acid; NaMN: nicotinic acid mononucleotide; NMN: nicotinamide mononucleotide; s.d.: standard deviation.

Supplemental Table 10: Concentrations of NAD and related metabolites in plasma of female mice of various *Nadsyn1* genotypes fed various NAD precursor diets for 17 days

Diet + Supplement	Sufficient			Limited			Limited + NA		Limited + NaMN		Limited + NMN	
***Nadsyn1* Genotype**	**+/+**	**+/-**	**-/-**	**+/+**	**+/-**	**-/-**	**+/-**	**-/-**	**+/-**	**-/-**	**+/-**	**-/-**
TRP	101750 ± 25815	101215 ± 26244	101017 ± 29060	55989 ± 24062	61038 ± 31591	97114 ± 25933	74021 ± 20424	79279 ± 12863	61004 ± 22058	87248 ± 22948	64258 ± 22369	74709 ± 39551
KYN	1204 ± 301	747 ± 159	808 ± 252	784 ± 310	896 ± 566	1332 ± 729	1537 ± 453	917 ± 155	728 ± 191	1219 ± 444	962 ± 222	1181 ± 458
KA	80.4 ± 32	72.7 ± 30	70.4 ± 27	18.0 ± 7.3	27.0 ± 20	53.0 ± 43	32.3 ± 20	34.8 ± 18	19.8 ± 8.5	60.7 ± 38	28.2 ± 20	38.6 ± 18
AA	53.2 ± 13	42.5 ± 19	40.6 ± 19	79.0 ±49	30.5 ± 7.9	58.9 ± 48	59.8 ± 44	38.9 ± 13	30.6 ± 12	49.1 ± 29	38.3 ± 14	49.3 ± 22
NA	<LOD	<LOD	50.4 ± 9.3	<LOD	<LOD	32.2 ± 11	<LOD	19.7 ± 8.5	<LOD	47.1 ± 11	<LOD	15.6 ± 4.1
NAR	5.20 ± 6.5	19.2 ± 18	2748 ±1704	<LOD	<LOD	214 ± 215	0.140 ± 0	226 ± 244	24.8 ± 36	217 ± 113	<LOD	316 ± 414
NaMN	<LOD	<LOD	881 ± 194	0.817 ± 0.50	0.747 ± 0.30	407 ± 277	0.895 ± 0.43	338 ± 356	51.8 ± 88	459 ± 324	0.615 ± 0.064	188 ± 98
NAD	9.77 ± 3.5	6.98 ± 4.8	15.6 ± 12	6.76 ± 1.2	12.3 ± 7.0	9.18 ± 14	12.9 ± 11	7.62 ± 4.5	6.55 ± 2.6	4.75 ± 4.3	13.4 ±12	29.4 ± 27
NAM	1938 ± 489	1815 ± 395	667 ± 69	681 ± 225	664 ± 125	277 ± 204	667 ± 150	166 ± 73	783 ± 490	184 ± 72	699 ± 148	707 ± 184
NMN	3.73 ± 1.9	2.68 ± 0.80	3.36 ± 1.8	2.08 ± 0.62	3.22 ± 1.6	2.15 ± 1.4	4.19 ± 1.6	1.69 ± 0.96	2.12 ± 1.4	1.58 ± 1.1	4.48 ± 3.0	3.23 ± 2.6
NR	3.60 ± 1.3	3.35 ± 0.39	2.44 ± 0.28	2.00 ± 0.14	2.28 ± 0.68	1.29 ± 0.34	2.47 ± 0.48	1.05 ± 0.16	1.66 ± 0.37	1.20 ± 0.11	2.00 ± 0.29	2.76 ± 0.84
2PY	380 ± 167	533 ± 159	44.4 ± 55	11.6 ± 4.3	59.8 ±71	1.28 ± 1.0	137 ± 42	2.59 ± 1.2	105 ± 81	24.9 ± 0	97.2 ± 59	19.3 ± 8.3
4PY	1552 ± 596	1945 ± 550	148 ± 187	51.5 ± 20	194 ± 256	7.68 ± 8.9	647 ± 174	3.14 ± 1.2	311 ± 274	22.1 ± 35	339 ± 232	52.0 ± 31

All values are in nM. TRP: tryptophan; KYN: kynurenine; KA: kynurenic acid; AA: anthranilic acid; NA: nicotinic acid; NAR: nicotinic acid riboside; NaMN: nicotinic acid mononucleotide; NAM: nicotinamide; NMN: nicotinamide mononucleotide; NR: nicotinamide riboside; 2PY: N1-methyl-2-pyridone-5-carboxamide; 4PY: N1-methyl-4-pyridone-3-carboxamide. <LOD: below limit of detection (signal:noise < 3). Nicotinic acid adenine dinucleotide (NaAD) was not detected in any of these samples.

Supplemental Table 11: Concentrations of NAD and related metabolites in plasma of pregnant *Nadsyn1+/-* and *Nadsyn1-/-* mice at 11.5 days of gestation

**Diet + Supplement**	**Sufficient**	**Limited**	**Limited +NMN**	**Limited +NMN**
***Nadsyn1* Genotype**	**+/-**	**+/-**	**+/-**	**-/-**
NA	< LOD	< LOD	< LOD	< LOD
NAR	< LOD	3.24 ± 3.7	2.99 ± 0.028	83.3 ± 45
NaMN	< LOD	< LOD	< LOD	106 ± 47
NAM	1608 ± 468	1042 ± 558	956 ± 320	799 ± 188
2PY	762 ± 243	166 ± 187	169 ± 93	21.4 ± 10
4PY	875 ± 212	545 ± 677	466 ± 460	43.5 ± 27

All values are in nM. NA: nicotinic acid; NAR: nicotinic acid riboside; NaMN: nicotinic acid mononucleotide; NAM: nicotinamide; 2PY: N1-methyl-2-pyridone-5-carboxamide; 4PY: N1-methyl-4-pyridone-3-carboxamide. <LOD: below limit of detection (signal:noise < 3).

Supplemental Table 12. Total NAD levels in E11.5 embryos generated under various dietary conditions and mating schemes

M	P	Embryo	Diet + Supplement	NAD (nmol/mg protein ± s.d.)	n	p
+/-	+/-	+/+	Sufficient	2.85 ± 1.06	6	0.7089
+/-	+/-	+/-	Sufficient	3.20 ± 0.993	14	
+/-	+/-	-/-	Sufficient	2.92 ± 0.928	8	
+/-	+/-	+/+	Limited	2.71 ± 1.07	11	0.2851
+/-	+/-	+/-	Limited	2.97 ± 1.04	16	
+/-	+/-	-/-	Limited	2.10 ± 1.03	5	
+/-	+/-	+/+	Limited + NMN	3.49 ± 0.260	4	0.1475
+/-	+/-	+/-	Limited + NMN	3.86 ± 0.489	12	
+/-	+/-	-/-	Limited + NMN	3.43 ± 0.584	9	
-/-	+/-	+/-	Limited + NMN	2.48 ± 0.576	26	<0.0001
-/-	+/-	-/-	Limited + NMN	1.55 ± 0.259	20	

p values represent statistical comparisons between the *Nadsyn1* embryo genotypes of the respective dietary conditions and were done by one-way ANOVA except for the comparison between *Nadsyn1*+/- and *Nadsyn1*-/- embryos from *Nadsyn1*-/- mothers on *Limited Diet* + *NMN* (last 2 rows) which were done by a two-tailed Student's t-test. M: maternal *Nadsyn1* genotype; P: paternal *Nadsyn1* genotype; NMN: nicotinamide mononucleotide; s.d.: standard deviation.

Supplemental Table 13. NADSYN1 cloning and genotyping primers

Variant	Primers	Use
Arg127Cys	5’-AACTACCGCGAGCTGTGCTGGTTCACC-3’	Cloning
Arg127Cys	5’-GGTGAACCAGCACAGCTCGCGGTAGTT-3’	Cloning
Cys175Tyr	5’-TGGAAGTGAGATCTATGAGGAGCTCTGGAC-3’	Cloning
Cys175Tyr	5’-GTCCAGAGCTCCTCATAGATCTCACTTCCA-3’	Cloning
Ala363Val	5’-GGGTGGACAGCGTAGCCACCGCCTG-3’	Cloning
Ala363Val	5’-CAGGCGGTGGCTACGCTGTCCACCC-3’	Cloning
Asp587Asn	5’-GGTGTCCCAGACCAACGAGGAAGATATG-3’	Cloning
Asp587Asn	5’-CATATCTTCCTCGTTGGTCTGGGACACC-3’	Cloning
-	5’-GGCTGTTCTCCCTTCCCTTC-3’	WT/mutant mouse genotyping
-	5’-CTACCCTGGGCAGACTTTGG-3’	WT/mutant mouse genotyping
-	5’-GTCTCCCTCAACAGGCAACCA-3’	WT mutant mouse genotyping
-	5’-GTCTCCCTCAACAGGCAACCG-3’	Mutant mouse genotyping

Supplemental Results

Molecular Modelling

Interpretations were made using Arpeggio (11) analysis carried out on the human crystalized NADSYN1 protein (PDB: 6OFB) and relate to in vitro functional assessment summarized in Figure 3, B, C, and D. Relevant reference structures and protein mutants are presented in Supplemental Figure 2.

C49R

The C49R mutation was first reported in Szot, et al. (8) wherein it was suspected that loss of a disulfide bond to C53 in the glutaminase domain would result in mispositioning of Y51 and result in protein destabilization; Y51 is a hydrophobic component of the first major bottleneck of the ammonia channel and interacts with the highly conserved YRE loop (10). Orthologous mutation of Y51 results in reduced stability of the protein (21) in *M. tuberculosis*. While these interpretations were originally made before human NADSYN1 had been crystalized and its structure resolved, interestingly, Arpeggio analysis did not similarly identify a disulfide bond between Cys49 and Cys53 (>4 Å) in the human structure. Instead, the loss of the sulfur atom upon mutation to Arg resulted in a loss of polar and hydrogen bond interactions with Leu46 and Tyr57, while introduction of the larger side chain Arg penetrated deeper within the pocket, and resulted in hydrogen bond, polar, and hydrophobic interactions with Pro89 and Ser66, and clashes with Thr62. While these effects may explain the observed destabilization in vitro, these changes occurred beyond significant interaction distance of either ligand, suggesting that the observed in vitro effects on affinity are indirect. Given that this mutant protein could be purified from transfected cells despite its reduced stability (8), and that this variant occurs in the N-terminal glutaminase domain (Figure 2), it is likely that it also retains partial synthetase activity.

R127C

R127 points towards the YRE loop of an opposing NADSYN1 monomer but resides just proximal to the YRE loop (aa120-125) within the same monomer. In the presence of bound glutamine at the glutaminase active site, R127 makes polar contacts to G121 of the YRE loop and Y58 of the same chain, while also interacting with D55 via an extensive ionic and hydrogen bond network. Mutation to cysteine at this site retains the capacity to hydrogen bond Y58 but loses the capacity to bind D55, thereby drastically weakening the hydrogen bonding network, which may stabilize the YRE loop within the glutaminase domain and potentially the link between two adjacent glutaminase domains of separate monomers. The R127C mutant therefore exhibits ~8-fold reduction compared to wild-type NADSYN1 in the presence of glutamine (reduced glutaminase activity), while less drastic reduction of ~2.7-fold reduction in the presence of ammonia, independent of glutaminase activity. This suggests that there is a degree of perturbation to overall activity by this variant, but primarily by reducing the capacity to use glutamine as substrate. Similar observations were made following in silico analysis, where mild effects were permuted, at a considerable interaction distance of 11.4 A.

C175Y

Within the glutaminase domain, a highly conserved catalytic triad referred to as CEK (cysteine-glutamine-lysine) is involved in glutamine hydrolysis. Within this triad, the nucleophilic cysteine is C175. C175 also forms part of a hydrophilic patch of

glutaminase active site residues creating the first major bottleneck of the glutamine tunnel. Bieganowski, et al. (22) assessed the impact of C175A mutation in yeast, showing that the resultant strain was inviable. Interestingly, it was shown that this mutant exhibited at >5000-fold reduction in glutamine-dependent NAD synthetase activity and a >250-fold reduction in glutaminase activity that was not further reduced when NaAD was not provided as substrate. Additionally, a <2-fold reduction in ammonia-dependent NAD synthetase activity was observed, indicating that, like human NADSYN1, ammonia-dependent synthetase activity was largely independent of a functional glutaminase domain. A similar neutral substitution to this conserved residue in *M. tuberculosis* C176A$^{C175A\ (human)}$ has been shown to completely lose glutamine-dependent activity while retaining 90% of its original ammonia-dependent synthetase activity, indicating that this residue is essential for hydrolysis of glutamine (23, 24). It has additionally been suggested that the primary contribution to glutamine binding is via the formation of a covalent bond between the sulfur of C175 and the δ-carbon of glutamine (21). These observations in orthologues are similar to results in this study – complete lack of glutaminase-dependent NADSYN1 activity (~162.6-fold reduced) while retaining most of its ammonia-dependent activity (~1.4-fold reduced). Arpeggio analysis suggests that C175 forms strong hydrogen bonds and polar interactions with E45, which is required for glutaminase activity. Some of these polar interactions are specifically mediated by the sulfur atom, which also interacts with S200 and G201 via a hydrogen bond network. Mutation to tyrosine loses the sulfur-mediated polar and hydrogen bond interactions, resulting in a less stabilized E45. This loss of E45 stabilization within the glutaminase pocket may explain the observed reduction in glutamine affinity in vitro and in silico.

A363V

Within the ATP-binding site of NADSYN1, AMP and MgPPi are bound in a deep cleft with one Mg ion coordinating to three O atoms of PPi, to the main chains of ATP-binding site residues V360 and D361 (the “ATP pyrophosphate fingerprint”), and a Cl- atom (10). Given the proximity of the A363 residue to these deeply pocketed residues, a conservative A363V mutation could mildly destabilize this cleft, thereby reducing effective closure of the P2 loop. In this regard, the A363V mutant exhibited the least reduced overall activity compared to WT, but to a similar degree when exposed to either glutamine or ammonia as substrates. P2 loop closure is necessary for synthetase activity. Therefore, in the presence of glutamine, this mutant exhibits ~2.1-fold reduced activity relative to WT, and in the presence of ammonia exhibits ~2.5-fold reduced activity. Arpeggio analysis indicates that the conservative A363V mutation results in a more extensive polar bond network at the mutation site, where the mutant participates in polar interactions with nearby G359, V360, and S362 residues, suggesting a possible displacement of the neighboring loop, which may lead to the observed reduction in ATP affinity.

A573T

This variant was first published in Szot, et al. (8) who showed a reduced glutamine-dependent activity of ~342-fold. Here we show a similar (non-zero) ~444-fold reduced glutamine-dependent activity under identical conditions. Supplying with ammonia shows equally reduced activity ~424-fold suggesting that it is indeed altered NaAD binding at the synthetase domain that prevents normal NAD generation. Differences in interaction profiles between alanine and threonine at A573 suggest a more stable interaction is created between the A573T mutant and NaAD

and ATP, mediated via hydrogen and polar bonds. The nature of these interactions suggest that the reaction equilibrium may be shifted, resulting in ATP depletion via lower disassociation with threonine, which in turn results in the observed lower enzymatic activity.

D587N

In the presence of glutamine, the D587N mutant exhibited ~187.3-fold reduced activity, whereas with ammonia had ~175.5-fold reduced activity. This similarity in reduced activity with both precursors supports the idea that synthetase activity is perturbed, while passage of ammonia (via N-terminal glutaminase activity) may be unaffected by this missense change. In yeast, neutral substitution to alanine at this residue results in yeast that remain viable, while purified yeast-expressed mutant protein exhibits 44% that of wild-type glutamine-dependent and 18.7% ammonia-dependent synthetase activity (25). The authors classified this mutant as a synthetase mutant with poor synthetase activity that retained substrate synergism meaning that, in the presence of NaAD binding at the active site, glutaminase activity is wastefully increased ~187-fold compared to that of wild-type (25). This indicates that even neutral amino acid substitution at this residue may cause structural adjustments similar to that of NaAD binding. Rizzi, et al. (26) and Jauch, et al. (27) have also structurally identified this residue as necessary for NaAD binding in orthologous *B. subtilis* and *E. coli* NAD synthetases, respectively. With respect to the human structure, the D587 is involved in the stabilization of NaAD at the active site (10). D587 is positioned within the P2 loop (alongside A573 and L575, which also hydrogen bond NaAD at specific sites) and specifically hydrogen bonds Y595 and A571, while also mediating an ionic bond with K544, thereby stabilizing closure of the P2 loop. D587N, on the other hand, loses the hydrogen bond at A571 and ionic bond at K544, instead forging a polar interaction with Y525 and an amide-amide interaction with A571. This change in interaction profiles is thought to destabilize the P2 loop and NaAD binding at the active site and explain why synthetase activity is equally reduced in the presence of either co-substrate.

Clinical Presentation of Affected Individuals

No patient gave consent for clinical photos to be published.

F1.II.1

The parents of proband (F1.II.1) are not known to be consanguineous but are from a small Jewish community in Uzbekistan. The proband presented at 19 weeks gestation. Fetal ultrasound at 12+2 weeks gestation showed a nuchal translucency of 6.7 mm and absent nasal bone (HP:0025706). A detailed fetal ultrasound at 19 weeks gestation showed forehead and pedal skin edema (HP:0010741), right jugular sac, and two vessel cord (P:0001195). Both kidneys were seen and were normal, and echocardiography was normal. Repeat ultrasound showed no abnormalities apart from the forehead edema and pedal edema. At 30 weeks gestation, intrauterine fetal demise was noted. Delivery was induced and on external examination we noted abnormal facial features with a short and slopped forehead (HP:0000290), deep set eyes (HP:0000490), thin lips (HP:0000233), and retrognathia (HP:0000278). There were deep-set and hypoplastic fingernails (HP:0001804) and toenails (HP:0001800). Autopsy was declined. This baby was found to be homozygous for a mutation in the *NADSYN1* gene (c.379 C>T p.R127C).

Prior genetic testing: Amniocentesis was performed and showed a normal microarray analysis. A RASopathy gene panel was also normal.

F2.II.3

The proband (F2.II.3) was the third child of healthy, young, and non-consanguineous White parents from northern France, born after a spontaneous pregnancy and antenatally suspected diagnoses of aortic coarctation (HP:0001680) and left superior vena cava (HP:0005301). She was born at 38 weeks with a weight of 2630 g (-1 SD), length 44 cm (-2 SD), and head circumference 33 cm (0 SD). Cardiac surgery was performed at 6 days old. Additional investigations showed skeletal abnormalities: costal fusion, Chiari type I malformation (HP:0007099), and complex bilateral inner ear malformations (HP:0011390); the right ear had a common cavity with rudimentary semicircular canals, the left had malformation of the posterior labyrinth with an enlarged vestibular aqueduct resulting in severe cochlear dysplasia IP type I. She had bilateral profound hearing loss (HP:0008619), requiring bilateral cochlear implantation. Decompressive surgery was performed because of her cervical-skull malformation (HP:0002678). No kidney nor eye abnormalities were noted. Due to feeding difficulties, nutrition ("Nutricia Infantrini", 200 mL/day) was provided via enteral tube. At 27 months old, F2.II.3 walked unassisted, speech had improved, and she was able to follow an ordinary education. At 5 years old, she was 102 cm in height (-1 SD), 17 kg (0 SD), with a 48 cm head circumference (-2 SD). Family history was uneventful. No medications or multivitamins were taken during pregnancy.

Prior genetic testing: Array CGH showed a 958 kb duplication: arr[GRCh37]2p25.3(883836_1842130)x3, inherited from her healthy mother. Gene panels (deafness and ID) were normal.

F3.II.3

Patient F3.II.3 was born via normal vaginal delivery at term to consanguineous parents (second cousins) of Pakistani origin. Due to fetal respiratory distress, the proband was transferred to the Neonatal Intensive Care Unit (NICU) and commenced on antibiotic treatment for sepsis of unknown origin. F3.II.3's inflammatory markers were consistently high throughout his stay in the NICU, and no cause was identified despite multiple blood cultures and a broad septic screen. Bilateral undescended testes were recognized on admission to the NICU, and MRI of the head and spine identified hemivertebra (HP:0002937) from T8-L1. Intra-cranial structures were normal. The spinal MRI demonstrated fluid in the right shoulder and left hip joints. A washout was performed on 10th October 2020, but there was no growth from the fluid. An echocardiogram and renal ultrasound scan were normal. No supplements were recorded to be taken during pregnancy.

Of note, in the first few days of life, a number of nodules were identified on the occiput and in the post-auricular region. These hard nodules grew in size relatively quickly, accompanied by a rise in inflammatory markers. The etiology of these lesions remains unclear. An ultrasound scan suggested that these were well vascularized lesions, making a biopsy technically challenging.

This inflammatory process appeared to settle over the course of a few weeks and the patient was discharged at five weeks of age. He was reviewed regularly in the first few months of life as he required three blood transfusions over the course of four months due to anemia (HP:0001903). Again, this resolved spontaneously, and no

further transfusions were required after six months of age. The hematology team felt that the most likely explanation was exaggerated physiological anemia.

He was slightly late to crawl, at 12 months of age, but he walked at 15 months of age. At the age of 19 months, he was using babble appropriately and he plays well with his siblings. He uses a pincer grip. Orchidopexy was performed to correct his left undescended and right retractile testes (HP:0000028). No significant limb abnormalities were identified; however, toenails were slightly hypoplastic (HP:0001800) with overlapping toes (HP:0001845) when assessed in the newborn period. At 20 months of age, he was recorded with significant short stature (HP:0004322) with a height of 74.1 cm (-3.66 SD), and a weight of 10.4 kg (-0.82 SD). Both parents are asymptomatic with no family history of similar anomalies and identified to be heterozygous carriers of the *NADSYN1* variant. The proband is the youngest of three children with an older brother and sister, aged eight and nine, respectively.

Whole blood and plasma were collected under fasting conditions from the proband and parents one year after his last blood transfusion, no longer anemic, aged 19 months. The proband was not on any specific NAD-boosting supplement during this time.

Prior genetic testing: Initial genetics assessment of the proband involved both array comparative genomic hybridization and quantitative fluorescent PCR, identifying no chromosomal abnormalities. Subsequent rapid trio whole exome sequencing was then performed on DNA extracted from blood of the proband and parents, identifying a homozygous *NADSYN1* variant (NM_018161.5: c.145T>C p.C49R) as the only candidate variant of note in the proband. This variant was initially reported as of uncertain significance. Both siblings are asymptomatic but have not been assessed for carrier status of this variant.

F4.II.2

The proband (F4.II.2) is the second pregnancy of non-consanguineous parents of Tunisian ancestry. The mother was 30 years of age at the time of delivery, with one spontaneous miscarriage and one termination of pregnancy for severe heart defects before (F4.II.1) and after (F4.II.3) the birth of the proband, respectively. The pregnancy was uncomplicated and followed by routine prenatal care, resulting in a normal vaginal delivery. Her birth weight was 2.7 kg (10% - WHO) and birth length was 44 cm (<2% - WHO). Coarctation of the aorta (HP:0001680) was diagnosed at birth and was repaired within the first week of life. Following this, due to a decrease in left ventricular function and development of a myocardial infarct, she was also found to have anomalous left coronary artery arising from pulmonary artery (ALCAPA) (HP:0011638). This was ligated at age 2 weeks and she has had no cardiac surgeries since with no remaining cardiopulmonary symptomology. She did not require subacute bacterial endocarditis prophylaxis. She is known to have a left-sided superior vena cava (HP:0005301) which is asymptomatic.

Since early childhood, the proband has a known history of multiple skeletal anomalies including butterfly vertebrae (HP:0003316), hemivertebrae (HP:0002937), spina bifida occulta (with sacral dimple) (HP:0003298), and kyphoscoliosis (HP:0002751) with lumbar lordosis. She has not required bracing or surgery of the spine. In early childhood she carried a clinical diagnosis of spondyloepiphyseal (and spondylothoracic/costal) dysplasia (HP:0002655). She has a low-lying spinal cord which has not been clinically significant. She has had episodic back pain for which

she received physical therapy. While the proband is short in stature (HP:0004322), she received growth hormone for 9 years and Lupron treatment taken once a month for less than a year, she has been able to adapt and function quite normally.

The proband had some gastrointestinal difficulties in her first year of life including gastroesophageal reflux disease (HP:0002020) and slow gastric emptying for which she received nasogastric tube feeds until she was one year of age, however feeding issues did not abate. As a child, she continued to have poor appetite but was able to take full nutrition by mouth, albeit with an aversion for certain food textures. She followed with an orthodontist for palate expansion and wore a retainer for a period of time, followed by braces and again a retainer. She has a history of constipation (HP:0012450), which may have contributed to her urinary history.

The proband has a history of multiple recurrent urinary tract infections (UTIs) (HP:0000010) including pyelonephritis (HP:0012330) over the years, which was managed by increasing fiber and water in the diet as well as a strict timed voiding schedule. She is followed by urology for this and had an extensive workup both anatomically and immunologically. Her kidneys appeared to be anatomically low in position (HP:0100542) from abdominal imaging, however normal renal ultrasound, normal renal function, and normal urodynamics (via voiding cystourethrogram) have been documented.

When she was five and six years old, in addition to the UTIs, the proband had a pattern of superficial infections (HP:0001581) of the skin in consecutive summers requiring antibiotics which prompted an immunological work up. Her complete blood count was normal, but absolute CD8+ and CD3+CD25+ lymphocytes were low. Immunoglobulin testing was normal, as was vaccine titer testing for streptococcal pneumoniae, diphtheria, and tetanus. She has a recurrent history of single ear infection (HP:0410018), affecting the small ear canals. Follow up immunology testing for possible immunodeficiency was unremarkable, and her various recurrent infections eventually resolved.

Other body systems are largely unaffected. Her hearing has been considered normal, and by the time she was 11 years old she had myopia which was corrected with glasses. Aside from her 37-day NICU stay, primarily due to her cardiac history and surgeries, the proband has subsequently been quite healthy, with no hospitalizations following the newborn period.

Developmentally, the proband obtained all gross and fine motor skills as well as speech milestones on time. She never had any periods of regression. By age 5 she was able to skip, run, climb, and would use a stair rail to go up and down stairs, primarily using the rail due to her short stature. Fine motor skills at age 5 included being able to legibly write her name, do math, self-feed, and self-dress including the use of buttons and zippers, though she still needed assistance to tie her shoes at this age. Intellectually she was always on target or advanced. She attended school in a conventional classroom once she was not receiving NG feeds, and later was enrolled in a gifted and talented program where she performed well academically. She has normal social skills. Since a young age she has participated in various high-level activities such as karate, gymnastics, volleyball, ballet, and swimming. She has played piano and guitar as well.

Her physical exams have regarded different features over the years. She has been short in stature since birth. At age 5 years her weight was 13.3 kg and her height was 96 cm, both of which were less than the third percentile (CDC). While at age 6

she had a normal upper to lower segment ratio of 1.074, by age 8 years, this was slightly reduced at 0.95, and by age 12 years this was reduced to 0.78. At age 5 and subsequently, she has been described as a well-appearing non-dysmorphic female, though on previous exams she had been described as having epicanthal folds (HP:0000286) and a somewhat high arched palate (HP:0000218), delayed eruption of secondary teeth (HP:0000696), as well as short 4th metacarpal (HP:0010049) on her left side and upturned nails (HP:0001597). Slightly blue sclera (HP:0000592) have been noted on some exams. She has a hemangioma (HP:0001028) over the coccyx. Her cardiac surgery scar shows slight widening due to soft skin (HP:0000977) but is otherwise normal in appearance. Her heart exam revealed a grade 1 systolic murmur with a normal heart rate and blood pressure for age in earlier childhood, but later exams do not feature a murmur. She has a slight pectus excavatum (HP:0000767), mild pes planus (HP:0001763), some generalized hyperflexibility to her joints (HP:0005692), and although with a history of kyphoscoliosis, this has been subtle. She did have some winging to the scapulae (HP:0003691) by age 6 years, and lumbar lordosis noted by age 8 years, and a slight rib hump by age 11.5 years.

At age 19, she is well-appearing and non-dysmorphic. She continues to have subtle kyphoscoliosis, with lumbar lordosis and a rib hump consistent with a thoracic curve. Her heart exam is unremarkable including normal blood pressure. Other aspects of her exam remain unchanged.

After receiving a diagnosis of NADSYN1-dependent Congenital NAD Deficiency Disorder, nicotinic acid levels in the plasma were measured at 11.5 nM. An untargeted screen of plasma metabolites was performed by Baylor Genetics, identifying no significant metabolic abnormalities, with very mild alteration to few NAD-related metabolites (Supplemental Table 4). She has subsequently begun a supplementation scheme with nicotinamide at a dosage of 250-500 mg/day (Nutricost Niacinamide capsules).

Prior genetic testing: She had a normal karyotype and a normal microarray (BAC array) analysis of 622 loci using 1887 BAC clones for the sub-telomeres, pericentromeric regions, and known genetic syndromes which detected no abnormalities. At age 5, a “DLO gene test” was negative. Specific gene sequencing tests for *SHOX* and *JAG1* were performed and resulted negative, as did testing of a panel of genes (*DLL3, MESP2,* and *LFNG*) known to be associated with spondylothoracic dysplasia. Sequencing of *NOTCH2* gene was also discussed but not performed. A chromosome microarray was performed showing a benign 63 Kb deletion identified in Xp22.33 which is located proximal to the *SHOX* gene and was determined to be paternally inherited. At age 12 the proband underwent trio whole exome sequencing at a clinical genetic diagnostic laboratory showing no variants in disease-associated genes; however, a heterozygous de novo variant NM_012395.3(*CDK14*):c.926A>G p.(Q309R), a novel disease-gene at the time, was identified. The original clinical whole exome trio data was reanalyzed under a research protocol (PGDP) at the age of 17 years, and re-sequenced months later under the same research protocol due to low coverage of the original clinical exome, which newly identified compound heterozygous missense variants in NM_005529.7(*HSPG2*):c.11562G>A p.(E2828K); c.4681G>C p.(P1534R). While a homozygous *NADSYN1* variant was also identified, the gene was not a focus of research at that time. Two years later, the parents (who have expertise in molecular genetics themselves) reviewed the candidate variants identified by the research

protocol. The parents connected to the scientific literature on the now recurrent variant NM_018161.5(*NADSYN1*):c.1717G>A p.(A573T), which was agreed by all involved parties (the family, PGDP, and *NADSYN1* experts contacted by the family) to be the diagnostic variant for the proband, age 19 at the time. The variant was then confirmed by Sanger sequencing in a clinical diagnostic genetic laboratory and classified as pathogenic. No DNA analysis was performed on F4.II.1 or F4.II.3.

F5.II.1

F5.II.1, a male child, was born at 41 weeks to White Australian parents via a normal pregnancy and caesarean section for breech presentation; growth parameters are not available. The proband was slender at birth and had a brief cyanotic episode at first weigh which resolved promptly. There was mild jaundice in the neonatal period that did not require phototherapy. No antibiotics were given. He also experienced sleeping difficulties (HP:0002360), was irritable, and required sleep therapy.

He presented to a hearing genetics clinic at age 9 years. While he passed the neonatal audiology screening tests, he was subsequently diagnosed with mild stable bilateral sensorineural hearing loss (HP:0008619) affecting high frequencies. This had been detected by the school nurse. He had seen a paediatrician and had a past medical history of bicuspid aortic valve (HP:0001647) with coarctation of the aorta (HP:0001680) which was surgically stented. Developmental milestones had been relatively normal but complicated by hypotonia (HP:0001252) and a diagnosis of mild-to-moderate autism spectrum disorder (HP:0000729) (Level 2) with a corresponding Full-Scale IQ score of 76. He had hyperextensible joints (HP:0001382). Craniofacial abnormalities were noted including a high-arched palate (HP:0000218), ptosis (HP:0000508), retrognathia (HP:0000278), and a long flat philtrum (HP:0000319). There was clinodactyly (HP:0030084).

During pregnancy, the mother (F5.I.2), a dental nurse, experienced negligible exposure to cleaning products, and no exposure to X-rays. There was also mild exposure to massage oils with apricot oil base, and various other combinations handled until four months gestation. Topical cortisone cream (1%) and light therapy were applied to treat psoriasis. Ventolin and Seratide medications were taken infrequently during pregnancy for asthma, while anti-depressants were taken throughout. Elevit pre-conception & pregnancy Multivitamins were taken daily throughout pregnancy.

There was no known family history of congenital malformation. A deficit in hearing was observed in the father (F5.I.1). A paternal uncle has been diagnosed with an unspecified condition causing deterioration of the bones in his wrist, while the paternal grandmother (deceased) was diagnosed with non-Hodgkin's lymphoma. The proband, father, sister (F5.II.2) and paternal half-brother all also share a history of hyperextensible joints.

Paediatric investigations for the aetiology of the hearing loss included normal results for blood thyroid-stimulating hormone, Cytomegalovirus IgG and brain and inner ear. Ultrasound of the kidneys, ureters, and bladder did not detect any abnormalities. A vertebral X-ray was also normal.

Prior genetic testing: Given the phenotypic clustering, syndromic deafness conditions were initially suspected including Townes-Brocks and Holt-Oram syndrome. Screening for mutations in *SALL1, TBX5*, and *GJB2* did not identify any mutations. Microarray did not detect any cytogenetic abnormalities. Exome analysis and re-analysis of the patient and parents did not reveal any competing genetic variants

besides those in *NADSYN1* (c.1088C>T p.Ala363Val; c.1765-7T>A). This splice variant was previously shown to cause three protein-truncating events: (1) p.Glu589Hisfs*7; (2) Glu589Valfs*8; (3) exon 19 skipping (28). No genetic testing has been performed on the healthy sister.

F6.II.2

The proband (F6.II.2) was the second child born to first cousins of Lebanese descent. There was a family history of breast cancer in the maternal great grandmother but no other cancers. The patient's mother (F6.I.2) has mild intellectual disability, as does the paternal grandfather and a maternal aunt. The mother had pharyngoplasty in childhood for pharyngeal incompetence and speech problems.

The couple's first child (F6.II.1) was a male, born at 35 weeks gestation however he died at three months of age. He had severe congenital microcephaly (HP:0000252) (birth head circumference 27 cm (0.04%, Z -3.36)) with MRI demonstrating thin corpus callosum (HP:0033725), globally reduced cerebral volume with underdeveloped cortex, bilaterally mildly dilated cochlea (HP:0011388), vestibule (HP:0011387), and semicircular canals (HP:0011383). He demonstrated neurodevelopmental delays (HP:0012758) with frequent dystonic movements. Other features included markedly reduced subcutaneous fat (HP:0003758), scaphocephaly with (HP:0030799) bitemporal narrowing (HP:0000341), right unilateral lamboid craniosynostosis (HP:0001363), bilateral undescended testes (HP:0000028) and rocker bottom feet (HP:0001838) with deep plantar creases (HP:0001869).

Microarray in the son identified a 0.1 Mb homozygous deletion of region 7p22.1 that includes the *PMS2* gene. This result conferred a diagnosis of Constitutional Mismatch Repair Deficiency Syndrome (CMMRD; OMIM: 619101) however it did not explain his clinical presentation. Singleton exome analysis identified two variants of uncertain significance in *RTTN*, however these were ultimately downgraded to likely benign variants, and no cause for his presentation was identified. No other variants of interest were identified on the exome. Given his poor prognosis due to the undiagnosed neurodevelopmental disorder and the CMMRD, a decision was made to palliate the baby. He developed progressive irritability, feed intolerance, increasing respiratory secretions, and central apnoeas. He ultimately passed away at three months of age, never having left hospital following his birth.

Microarray confirmed both parents were heterozygous for the *PMS2* deletion, and therefore had Lynch syndrome.

The proband (F6.II.2) was delivered at 38 weeks by forceps assisted vaginal delivery; she died after 13 days. During pregnancy the ultrasound at 20 weeks identified multiple structural abnormalities involving the heart, brain, and skeleton, however the couple declined investigation during the pregnancy. There were no known teratogen exposures, maternal vitamin supplementation is unrecorded. Birth weight was 2.961 kg, length 46 cm and head circumference 34 cm; Apgar scores were 7 and 9 at 1 and 5 minutes, respectively. She required non-invasive respiratory support at delivery and was admitted to the Special Care Nursery.

Clinical examination identified dysmorphic features including microphthalmia (HP:0000568) and sclerocornea (HP:0000647) with fused left eyelid, low set ears (HP:0000369) with thickened helices (HP:0000391), a short broad neck (HP:0000470), mild upper limb rhizomelia (HP:0004991), and the impression of triphalangeal thumbs (HP:0001199) with proximal insertion. She had a high arched palate (HP:0000218) and bifid uvula (HP:0000193). Genital examination revealed a

bulging membrane at the vaginal introitus, imaging was suggestive of hydrocolpos (HP:0030711). Examination under anesthesia identified a large hydrocolpos with a single urogenital sinus with no vaginal opening and a normal anus. A pigtail drain was inserted transabdominally into the uterus and viscous fluid was drained. Fluid cytology did not reveal any malignant cells and there was no bacterial growth on culture. Cystoscopy revealed mildly trabeculated bladder (HP:0000014). Renal ultrasound identified both kidneys were normally grown (right 50th centile, left 95th centile) with mild bilateral prominence of the renal pelvis, likely related to obstruction due to the hydrocolpos.

Echocardiogram confirmed Tetralogy of Fallot (HP:0001636) with right sided aortic arch, dilated left ventricle with atrioventricular septal defect (HP:0006695), common aortic valve, right ventricular hypertrophy (HP:0001667), overriding aorta (HP:0002623) and mild-to-moderate pulmonary stenosis (HP:0001642).

Skeletal survey demonstrated generalized widespread diffuse sclerosis in keeping with osteopetrosis (HP:0011002), hypoplasia of the left orbit, and partial fusion of the posterior aspects of numerous ribs (HP:0000913) with only 10 pairs of ribs (HP:0030300) seen bilaterally. Multiple vertebral segmentation defects (HP:0003422) were present within the thoracolumbar region, the sternum appeared segmented, while in the pelvis the pubic (HP:0003173) and ischial bones (HP:0003175) were hypoplastic. There was unilateral fusion of the proximal radius and ulna, and the tarsal (HP:0010743) and carpal bones (HP:0005914) were relatively hypoplastic with increased density. Femora were somewhat short and broad with irregular metaphyses.

Cerebral MRI revealed a left retrocerebellar cyst causing mass effect and elevation of the left cerebellar hemisphere with a normal vermis that was underrotated. The cyst was in communication with the fourth ventricle which was slightly enlarged. The midline structures including corpus callosum were otherwise normal, and myelination was appropriate for gestation. There were normal cranial nerves and no features of a neuronal migration disorder. Left globe was hypoplastic with normal optic nerve.

An attempt to wean non-invasive respiratory support led to an acute obstructive episode, with progressive deterioration with intubation required on day 9 of life. Respiratory failure was considered secondary to pulmonary hypoplasia (HP:0002089) and the baby remained ventilator-dependent with increasing oxygen requirements. Given the poor prognosis and difficulties with ventilation, care was redirected to palliation on day 11 and she passed away on day 13.

Microarray confirmed the baby was heterozygous for the familial *PMS2* deletion, although this did not explain the presentation. There was also loss of heterozygosity across approximately 10% of the genome, in keeping with parental consanguinity.

Singleton exome sequencing was arranged under research (Broad collaboration) after death. This identified a homozygous variant in *NADSYN1* (c.524G>A p.C175Y) that was considered likely to explain the cardiac and skeletal features. The baby had a second homozygous variant in *TCIRG1* (c.702del p.I235Sfs*44). This variant was considered causative for the osteopetrosis phenotype.

Subsequently, the exome data from the deceased brother was reanalyzed; he was a carrier of both the *NADSYN1* and the *TCIRG1* variants, therefore the cause of his features remains unknown.

Prior genetic testing: None.

F7.II.3

The proband (F7.II.3) is the third child to consanguineous (second cousin) parents of Lebanese descent. His brother and parents are healthy, and there is a family history of malignant infantile osteopetrosis in four of eight of his assessed first cousins.

The 30-year-old mother (F7.I.2) had diet-controlled gestational diabetes and a previous first trimester miscarriage (F7.II.1). There was no known teratogen exposure during pregnancy. Concerns about congenital cardiac malformations were raised at 30 weeks' gestation, and fetal echocardiogram showed a double outlet right ventricle (HP:0001719) with hypoplasia of the left ventricle and mitral valve (HP:0001633), coarctation of the aorta (HP:0001680), and subaortic ventricular septal defect (HP:0011656). The male infant was born by elective caesarean at 39 weeks' gestation with a birth weight 2676 g (7th percentile), and length 45.4 cm (3rd percentile). Apgar scores were 9 at one and five minutes, respectively, and he passed meconium at birth. Spinal imaging showed multilevel vertebral segmentation defects at T8-T11 (HP:0100711), lower thoracic scoliosis (HP:0002943), bilateral rudimentary 1st ribs (HP:0000772), bifid 10th ribs (HP:0000892), and posterior fusion of the right 8th, 9th, and 10th ribs (HP:0000913); a deep non-communicating sacral dimple was also identified (HP:0000960). Brain MRI carried out due to microcephaly (HP:0000252) showed reduced white matter volume loss and delayed myelination at two months equivalent to that of 40 weeks' gestation. At four months, myelination was still delayed at a 6-8 week age-equivalent while white matter volume remained reduced with prominent frontal extra-axial cerebrospinal fluid space. No intracranial abnormalities were detected. Echocardiogram confirmed prenatally diagnosed congenital heart malformations, additionally identifying a large patent ductus arteriosus (HP:0001643) with right-to-left shunt. He had poor growth, measuring below the 2nd centile for weight, height, and head circumference at 12 months of age, with no abnormalities detected on nutritional assessment. Bayley Scales developmental assessment at 16 months of age had previously shown cognitive (HP:0001263) and motor delays (HP:0001270) and emerging communication skills. He learned to walk independently at 19 months and was noted to have some expressive language delay (HP:0002474), together with behavioral and emotional regulation difficulties (HP:0000708). His vision and hearing are normal.

On review at 20 months, the proband's weight was 9.1 kg (<1st centile), length 75 cm (<1st centile), and head circumference 45.5 cm (2nd centile). He was centrally cyanosed. He had mild micrognathia (HP:0000347) but no other craniofacial dysmorphism. There was no body asymmetry and limbs were proportionate, with no radial ray defects or digit abnormalities. His abdomen was soft with no hepatosplenomegaly. He had normal male genitalia. Examination of the spine showed lower thoracic scoliosis and a sacral dimple in addition to butterfly-shaped vertebrae at T8, T10, and T11 (HP:0003316). Neurologic exam was normal, and he had no neurocutaneous stigmata. Kidney and bladder ultrasound revealed normal appearance of both kidneys and bladder.

Staged surgical repairs of his cardiac lesion were undertaken at 1 week and 4 months of life, respectively, and heart failure medications commenced. Enteral feeds were fortified due to his poor growth with introduction of a nutritionally complete formula (Sustagen Kid Essential Nutritionally Complete; 55 g/day) at 12 months in addition to solids. His pediatrician arranged early intervention speech therapy support.

Following consultation with the metabolic team, the proband was commenced on NAD precursor supplementation in the form of nicotinamide at 5 mg/kg/d (50 mg/d) at 24 months of age (Blackmores Insolar Nicotinamide 500 mg tablets; 1 tablet crushed and mixed in 10 mL water, 1 mL administered daily). Whole blood, plasma, and urine were taken prior to and 6 weeks after NAM supplementation for assessment. Nicotinamide dosage was then increased to 10 mg/kg/d (100 mg/d) and maintained for 6 weeks. Whole blood and plasma were then collected for metabolic assessment.

The proband's most recent assessment was at the age of 3 years 2 months, 14 months post-commencement of nicotinamide supplementation. His pediatrician noted that he had shown excellent developmental progress and is at a developmental level consistent with age despite being at high risk of developmental vulnerabilities. Also noted were episodes of mild emotional and behavioral dysregulation with hitting episodes. He remains microcephalic (head circumference: 46.5 cm; <1st centile; Z = -2.8). His other growth parameters are also tracking below the 3rd centile (weight: 11.2 kg; Z = -2.3, and height 86 cm; Z = -2.3).

Prior genetic testing: Initial genetics assessments by array-CHG revealed no clinically significant genomic imbalances. Extensive long continuous stretches of homozygosity (LCSH) were identified in chromosomes 1, 2, 3, 5, 6, 7, 9, 10, 11, 13, 14, 15, 18, and 19, representing 9.5% of the genome and confirming stated parental consanguinity. Clinical exome analysis of the parent-proband trio identified a single likely-pathogenic homozygous variant in *NADSYN1* (c.524G>A p.C175Y) in the proband, with no competing candidate gene variants identified to explain the broader phenotype. A pathogenic homozygous variant in *TCIRG1* (c.702del p.I235Sfs*44), 3.4 Mb downstream of *NADSYN1* (11q13.4) and outside the regions of LCSH identified in F9.II.3, was identified to explain the osteopetrosis phenotype in the extended family. This variant was found in heterozygous state in the proband and parents.

F8.II.3

The male proband (F8.II.3) was the third pregnancy of a non-consanguineous White Australian couple, born at 39 weeks by standard vaginal delivery. Non-invasive prenatal testing had not identified any abnormalities. He had a birth weight of 3.1 kg, a head circumference of 33 cm, and body length of 53 cm. Initial examination identified camptodactyly of the left middle and little finger (HP:0009183). Spinal X-ray showed a subtle fusion of L4-L5 (HP:0030040) and a wedged appearance of L1. Abdominal ultrasound and echocardiogram did not reveal any abnormalities. Ophthalmological examination was also normal. He was noted to have some dysmorphic features including low-set ears (HP:0000369) and downslanting palpebral fissures (HP:0000494). From birth, he has had difficulties with weight gain and feeding (HP:0008872) and was under regular pediatric review.

At five months, the proband was gaining weight via a supplemented formula-based diet (Aptamil Gold+ Infant Formula; 200 mL taken daily at night). At six months, he was diagnosed with mild gross motor delay on a Griffiths score, which was mostly due to difficulties in sitting. At 14 months, he was noted to make developmental progress with a mild expressive language delay. At two years old he only had a mild expressive language delay (HP:0002194), continuing to be of short stature (HP:0004322) and low in weight (10 kg), with a head circumference of 46.5 cm, and height too difficult to measure. Dysmorphic features include hypertelorism

(HP:0000316), down-slanting palpebral fissures (HP:0000494), hooded eyelids (HP:0030820), and low-set posterior rotated ears (HP:0000368).

Two prior pregnancies were medically terminated after discovery of significant congenital malformation. During the first pregnancy (F8.II.1), first trimester cytogenetic screening revealed a high risk for Trisomy 21, Trisomy 18, and Trisomy 13. At 16 weeks, anatomy scan and subsequent fetal echocardiogram identified a hypoplastic left heart (HP:0004383). The pregnancy was terminated at 19 weeks. Post-mortem showed a non-dysmorphic male fetus with hypoplastic left heart syndrome characterized by a small globular left ventricle, aortic valve stenosis (HP:0001650), hypoplasia of the aortic arch (HP:0012304), mitral stenosis (HP:0001718), multiple bony abnormalities including a lower dorsal butterfly-like vertebrae (HP:0003316) or hemivertebrae (HP:0002937), L5 forme fruste butterfly vertebrae, and anomalous development of the first and second ribs (HP:0006593). During the second pregnancy (F8.II.2), first trimester cytogenetic screening identified a high probability for Trisomy 21, Trisomy 18, and Trisomy 13. Ultrasound scan performed at 16 weeks revealed a hypoplastic left heart (HP:0004383) with a large right atrium, right ventricle, and right ventricular outflow tract. The pregnancy was terminated at 18 weeks 5 days. Post-mortem showed transposition of the great arteries (HP:0001669), double outlet right ventricle (HP:0001719), small left ventricle, hypoplasia of the aortic arch (HP:0012304), a single right lung lobe and two left lobes (HP:0033998), prominent sandal gap on the right foot (HP:0001852), multiple segmental abnormalities of the mid and lower dorsal vertebral bodies (HP:0003422), a reduced number of ribs on both sides (HP:0000921) with posterior fusion of the left lower ribs (HP:0000902), and partial S1 and S2 fusions.

Maternal ages for all three pregnancies were 29, 30, and 31, respectively. There is no other family history of any congenital malformation, with both parents having additional children from previous relationships, all of whom are fit and healthy. Both terminated pregnancies and the proband have been confirmed to be homozygous for the *NADSYN1* (c.1717G>A p.A573T) variant by Sanger sequencing.

Prior genetic testing: No alternative genetic anomalies were detected via chromosomal microarray.

F9.II.4

The proband (F9.II.4) is the third pregnancy to consanguineous parents (grandparents are siblings) of Chinese descent. The pregnancy was uncomplicated with the father (F9.I.1) and mother (F9.I.2) aged 31 and 28 years old, respectively. Comprehensive phenotypic assessment of the proband identified mild craniofacial abnormalities including brachycephaly (HP:0000248), narrow palpebral fissures (HP:0045025), full lips (HP:0012471) and large nose (HP:0000448), and an absent uvula (HP:0010292). Additional abnormalities included mild scoliosis (HP:0002650) and bilateral posterior iliac crests (HP:0003796), a hyper nasal voice (HP:0001611) due to velopharyngeal insufficiency (HP:0000220) (corrected via sphincter palatoplasty), the right sclera was slightly injected with mild periorbital erythema, and inverted nipples (HP:0003186). Echocardiogram assessment was normal, as was a brain MRI and EEG, while renal ultrasound diagnosed mild left renal pelviectasis (HP:0010945). Concerning renal assessment, the most recent review identified right renal pelviectasis (SFU 1, improving after void) and slightly enlarged; left renal hydronephrosis (SFU 2, improving after void). Bladder distention was also noted with significant post-void volume, potentially representing a neurogenic bladder.

At 7 years of age, the proband presented with a photo-distributed rash (HP:0000992) with abdominal pain (HP:0002027) and leg weakness (HP:0007340). This episode lasted a month and required hospitalization. This severe rash appeared on his face involving the malar area, arms, ankles, and hands in a symmetric glove-like distribution with patchy involvement of his feet, including only sun-exposed areas, with sharp demarcations. At the time, differential diagnoses included pellagra and several inborn errors of metabolism, including Hartnup disease, mevalonic aciduria, and various types of porphyria, including porphyria cutanea tarda and variegate porphyria. This coincided with watery diarrhea that progressed to hematochezia, with associated severe abdominal pain, and pseudomembranous hemorrhagic colitis due to clostridium difficile. The patient was further diagnosed with pancytopenia (HP:0001876). Blood and bone marrow biopsies at the time were performed under suspicion of copper deficiency but blood copper levels were normal. Pancytopenia was characterized by 40% hypocellular marrow with serious atrophy/stromal damaged, marked erythroid hypoplasia with few ring sideroblasts, cytoplasmic vacuolization of hematopoietic precursors, reactive appearing lymphocytes and plasma cells. Skin biopsies were performed from samples of the left wrist and left foot revealing psoriasiform dermatitis (HP:0003765) with superficial epidermal vacuolization and compact hyperkeratosis (HP:0000962). The left wrist sample was further defined as ulcerous with dense dermal inflammation and adjacent epidermal necrosis. During hospitalization, the patient developed hypertension and required a personal trainer to relearn to walk, involving two episodes of abdominal pain and nausea with leg weakness recovering.

A year later, with recurrence of this rash, repeat biopsies were performed on the right medial thigh, left wrist, and right foot. Again, findings revealed a psoriasiform dermatitis with epidermal necrosis and subepidermal clefting, ballooning degeneration of the keratinocytes and ulceration. Pallor and necrosis of the epidermal keratinocytes was suggestive of a nutritional deficiency such as pellagra or acrodermatitis enteropathica, tryptophan metabolism disorders such as Hartnup disease and necrolytic migratory erythema.

At 11 years old, the proband experienced a similar pellagra-like episode characterized by a desquamating rash on the face, forearms, hands, and concurrent symptoms of an upper respiratory tract infection. Differential diagnoses included photosensitivity disorders, pellagra, eczematous dermatitis, polymorphous light eruption, nutritional deficiency, contact dermatitis, acute cutaneous lupus erythematosus, and Hartnup disease. Given the distribution of the desquamating rash, this was considered an episode of pellagra and treated with sunlight avoidance in combination with oral administration of nicotinamide (100-200 mg daily) starting at 100 mg/day. At this time, plasma and urine amino acids were at normal levels and a complete blood count did not reveal any cytopenia. The rashes were observed to improve after nicotinamide treatment and application of 0.1% Triamcinolone ointment. Additional phenotyping of the proband at this time characterized developmental delays in terms of speech development and articulation (HP:0000750), ADHD (HP:0007018), requiring special education classes.

The proband experienced a third, more severe, pellagra-like episode at age 12 lasting 2-3 months. The rash presented as erythematous and edematous skin on the arms and other parts of the body, highly suggestive of a photosensitive nutritional deficiency such as that of niacin, which had been assessed and previously shown to be at normal levels. Given repeated findings of acute epidermal necrolysis, balloon

cell degeneration and psoriasiform dermatitis as well as sub-epidermal clefting, annually recurring acroerythema was alternatively considered due to the identification of a hereditary lactate dehydrogenase M-subunit deficiency. The rash was treated with nicotinamide and Triamcinolone for two months before it resolved. At this time, blood nicotinic acid was measured at 764 nM. Months later, after nicotinamide treatment was stopped, the rash returned prompting reassessment of blood nicotinic acid levels (593 nM). At this time, all vitamin B12 metabolites were within a normal range. At 14 years old, he injured both knees playing routine sports requiring surgical repair; these injuries were consistent avulsion fractures of the anterior tibial tuberosity. A few days post-surgery he developed a red rash on his face, surrounding his mouth, and on his upper and mid-back with the perioral rash recorded to be quite severe and dark with some desquamation. The most recent pellagra-like episode occurred aged 15 years old at which point partial assessment of whole blood metabolites was performed within 2-3 days of the onset of the rash. Blood nicotinic acid levels were observed to be at 1064 nM at this time. During all three of the most recent pellagra episodes, blood nicotinamide was measured at <164 nM (limit of detection). The proband has remained on nicotinamide supplementation at 200 mg/day having not yet experienced a repeat pellagra-like episode.

Additional assessments of the proband included an erythrocyte porphyrin analysis, aged 11, identifying a slight increase in erythrocyte protoporphyrin with zinc predominance. At the time, blood UROD was low suggesting a deficiency but urine procalcitonin was within normal ranges so this was considered insignificant. Further biochemical testing of lactate dehydrogenase levels showed reduced levels of subunits I, II, and V.

Although there is no record of congenital malformation in the extended family, the father (F9.I.1) was diagnosed with a heart murmur of unknown etiology, and the mother (F9.I.2) has since passed away aged 35 years old from a brain aneurysm. The paternal grandfather had stomach cancer and died from lung cancer at 52 years old, diagnosed 49 years of age. The oldest sister (F9.II.1) was also free of congenital malformation and was first seen for genetics assessment at 21 years of age. She was born via C-section without complications; newborn bloodspot screening did not reveal any abnormalities. At 11 years of age, she was diagnosed with myopia and, by 20 years old, required surgical scleral buckling for retinal detachment. At 15 years of age, she was diagnosed with mild idiopathic scoliosis not necessitating treatment. In addition, she has exhibited intermittent bruising, heavy and irregular menstrual cycles, migraine headaches, numbness after long periods of sitting, chest pain and lightheadedness, polyuria and an incapacity to completely empty her bladder. Intellectual development was normal with only special education required in elementary school via English as a second Language class. She was also reported to have experienced a very mild photo-distributed rash, although clinical records surrounding this event are not available. Thyroid ultrasound, brain MRI and head CT did not reveal any abnormalities. Her younger sister (F9.II.2) is healthy and without congenital malformations. The youngest daughter (F9.II.3) is also reported to not exhibit any congenital anomalies but has experienced a milder form of the pellagra-like dermatitis at age 12 to that experienced by F9.II.4.

Prior genetic testing: For the proband, a porphyria *UROD* single gene test was negative. A chromosomal microarray identified long contiguous stretches of homozygosity covering the following regions:

arr[hg19] 1p13.2p11.2(113,276,155-121,339,317)x2
arr[hg19] 1q21.1q24.2(144,033,938-169,085,119)x2
arr[hg19] 1q24.2q25.2(170,521,375-176,990,417)x2
arr[hg19] 4p15.2p11(26,533,148-49,089,181)x2
arr[hg19] 4q11q13.3(52,686,799-75,780,094)x2
arr[hg19] 5q32q33.3(149,653,572-158,457,716)x2
arr[hg19] 6p12.2p11.1(52,513,888-58,741,497)x2
arr[hg19] 6q11.1q16.1(61,968,745-97,095,082)x2
arr[hg19] 11p15.1p11.12(19,554,416-51,563,636)x2
arr[hg19] 11q11q13.5(54,794,726-76,537,114)x2
arr[hg19] 15q23q26.3(68,718,268-99,458,481)x2
arr[hg19] 22q13.31q13.33(45,629,445-51,177,928)x2
A comprehensive lysosomal storage disease panel was also negative. Whole exome sequencing performed on the proband, father, and three sisters (F9.II.1, F9.II.2, F9.II.3) identified a heterozygous pathogenic variant NM_000552.5(*VWF*):c.5335C>T p.(Arg1779*), alongside a homozygous variant of uncertain significance (VUS) NM_001198904.1(*YY1AP1*):c.981A>T p.(Lys327Asn) inherited from the father, and the homozygous *NADSYN1* variant NM_018161.5(*NADSYN1*):c.271del p.(Met91Cysfs*11). F9.II.1 is heterozygous for the *VWF* and *NADSYN1* variants, and homozygous for the *YY1AP1* variant; F9.II.2 is heterozygous for both *YY1AP1* and *VWF* variants; F9.II.3 is homozygous for only the *NADSYN1* variant. No samples were available from the deceased mother for sequencing. Homozygosity of the *VWF* variant has been reported as a pathogenic cause of von Willebrand disease (29). Similarly, homozygosity of *YY1AP1* mutations cause Grange syndrome (30) although lack of functional testing concerning the specific *YY1AP1* variant in Family 9 has resulted in its classification as a VUS.

F10.II.2
The proband (F10.II.2) is the second pregnancy of non-consanguineous White parents of Slovakian descent. During pregnancy at 20 weeks of gestation, an atrioventricular septal defect (HP:0006695) was detected at fetal ultrasound. An amniocentesis was performed. Whole exome sequencing with a targeted panel of 4500 genes revealed two mutations in the *NADSYN1* gene. After extensive genetic counseling, the parents decided to continue the pregnancy. At 29 weeks of gestation, additional anomalies were seen: right hand polydactyly (HP:0001161) adjacent to the second digit with a pre-axial deviation and a crooked thumb, and left hand pre-axial deviation of the second digit (HP:0009468). Delivery of this male was uncomplicated at 38 weeks 2 days at a weight of 2985 g. Apgar 8/8/8. One week after birth, physical examination revealed several dysmorphic features: a pear-shaped face with full cheeks (HP:0000271), hypertelorism (HP:0000316) and an upslant of the eyes, a high nasal bridge (HP:0000426), upturned nostrils with a broad nasal tip (HP:0000455), retrognathia (HP:0000278), hypoplasia of the right ear and a dysplastic left ear (HP:0008551), short neck (HP:0000470), and a palatal cleft (HP:0000175). A biphalangeal 2nd digit was noted of the left hand (HP:0011297) with a thin thumb. On the right hand was a duplication of the 2nd digit (HP:0009946) and duplication of the nails with a hypoplastic thumb (HP:0001172). Rhizomelia was noted for both arms (HP:0004991). Vertebral anomalies were also observed including a T10 butterfly vertebra (HP:0003316), T11-T12 fusion (HP:0030039) and abnormal rib (HP:0000772) possibly due to the vertebral fusion. Echocardiogram revealed an atrioventricular septal defect (HP:0006695). Other anomalies included

glandular hypospadias (HP:0000807) and hypotonia (HP:0001252). Repeat examination at 5 months of age identified a failure-to-thrive (HP:0001508) with a weight of only 4750 g (2nd-3rd percentile). At this time, feeding had been performed via nasogastric tube. The proband exhibited stridor with low O_2 saturation, believed to be due observation of a supraglottic web and a distal tracheomalacia (HP:0002779), and developmental delay (HP:0001263). Brain MRI revealed no abnormalities. At the age of 8 months, a tracheostoma was placed because of respiratory insufficiency due to a narrow bronchial airway and the laryngeal web. The atrioventricular septal defect was closed at the age of 9 months. Hearing loss was noted in right ear, and loss of at hearing 70 dB in the left ear (HP:0000365). At the age of 12 months tracheomalacia (HP:0002779) occurred. At the age of 20 months artificial respiration was weaned a few hours per day. The kidneys were also observed to be small and dysplastic (HP:000110) while renal function was normal.

Supplemental References

1. Retterer K, Juusola J, Cho MT, Vitazka P, Millan F, Gibellini F, Vertino-Bell A, Smaoui N, Neidich J, Monaghan KG, et al. Clinical application of whole-exome sequencing across clinical indications. *Genet Med.* 2016;18(7):696-704.
2. Richards S, Aziz N, Bale S, Bick D, Das S, Gastier-Foster J, Grody WW, Hegde M, Lyon E, Spector E, et al. Standards and guidelines for the interpretation of sequence variants: a joint consensus recommendation of the American College of Medical Genetics and Genomics and the Association for Molecular Pathology. *Genet Med.* 2015;17(5):405-24.
3. Laver TW, De Franco E, Johnson MB, Patel KA, Ellard S, Weedon MN, Flanagan SE, and Wakeling MN. SavvyCNV: Genome-wide CNV calling from off-target reads. *PLoS Comput Biol.* 2022;18(3):e1009940.
4. McKenna A, Hanna M, Banks E, Sivachenko A, Cibulskis K, Kernytsky A, Garimella K, Altshuler D, Gabriel S, Daly M, et al. The Genome Analysis Toolkit: a MapReduce framework for analyzing next-generation DNA sequencing data. *Genome Res.* 2010;20(9):1297-303.
5. Wang K, Li M, and Hakonarson H. ANNOVAR: functional annotation of genetic variants from high-throughput sequencing data. *Nucleic Acids Res.* 2010;38(16):e164.
6. Strande NT, Riggs ER, Buchanan AH, Ceyhan-Birsoy O, DiStefano M, Dwight SS, Goldstein J, Ghosh R, Seifert BA, Sneddon TP, et al. Evaluating the Clinical Validity of Gene-Disease Associations: An Evidence-Based Framework Developed by the Clinical Genome Resource. *Am J Hum Genet.* 2017;100(6):895-906.
7. Sadedin SP, Dashnow H, James PA, Bahlo M, Bauer DC, Lonie A, Lunke S, Macciocca I, Ross JP, Siemering KR, et al. Cpipe: a shared variant detection pipeline designed for diagnostic settings. *Genome Med.* 2015;7(1):68.
8. Szot JO, Campagnolo C, Cao Y, Iyer KR, Cuny H, Drysdale T, Flores-Daboub JA, Bi W, Westerfield L, Liu P, et al. Bi-allelic Mutations in NADSYN1 Cause Multiple Organ Defects and Expand the Genotypic Spectrum of Congenital NAD Deficiency Disorders. *Am J Hum Genet.* 2020;106(1):129-36.
9. Jumper J, Evans R, Pritzel A, Green T, Figurnov M, Ronneberger O, Tunyasuvunakool K, Bates R, Zidek A, Potapenko A, et al. Highly accurate protein structure prediction with AlphaFold. *Nature.* 2021;596(7873):583-9.

10. Chuenchor W, Doukov TI, Chang KT, Resto M, Yun CS, and Gerratana B. Different ways to transport ammonia in human and Mycobacterium tuberculosis NAD(+) synthetases. *Nat Commun.* 2020;11(1):16.
11. Jubb HC, Higueruelo AP, Ochoa-Montano B, Pitt WR, Ascher DB, and Blundell TL. Arpeggio: A Web Server for Calculating and Visualising Interatomic Interactions in Protein Structures. *J Mol Biol.* 2017;429(3):365-71.
12. Yang H, Wang H, and Jaenisch R. Generating genetically modified mice using CRISPR/Cas-mediated genome engineering. *Nat Protoc.* 2014;9(8):1956-68.
13. Cuny H, Rapadas M, Gereis J, Martin E, Kirk RB, Shi H, and Dunwoodie SL. NAD deficiency due to environmental factors or gene-environment interactions causes congenital malformations and miscarriage in mice. *Proc Natl Acad Sci U S A.* 2020;117(7):3738-47.
14. Cuny H, Kristianto E, Hodson MP, and Dunwoodie SL. Simultaneous quantification of 26 NAD-related metabolites in plasma, blood, and liver tissue using UHPLC-MS/MS. *Anal Biochem.* 2021;633( ):114409.
15. Mori V, Amici A, Mazzola F, Di Stefano M, Conforti L, Magni G, Ruggieri S, Raffaelli N, and Orsomando G. Metabolic profiling of alternative NAD biosynthetic routes in mouse tissues. *PLoS One.* 2014;9(11):e113939.
16. Cuny H, Bozon K, Kirk RB, Sheng DZ, Broer S, and Dunwoodie SL. Maternal heterozygosity of Slc6a19 causes metabolic perturbation and congenital NAD deficiency disorder in mice. *Dis Model Mech.* 2023;16(5).
17. Alankarage D, Szot JO, Pachter N, Slavotinek A, Selleri L, Shieh JT, Winlaw D, Giannoulatou E, Chapman G, and Dunwoodie SL. Functional characterisation of a novel PBX1 de novo missense variant identified in a patient with syndromic congenital heart disease. *Hum Mol Genet.* 2019.
18. Nair AB, and Jacob S. A simple practice guide for dose conversion between animals and human. *Journal of basic and clinical pharmacy.* 2016;7(2):27-31.
19. Institute of Medicine (US) Standing Committee on the Scientific Evaluation of Dietary Reference Intakes and its Panel on Folate, Other B Vitamins, and Choline. *Dietary Reference Intakes for Thiamin, Riboflavin, Niacin, Vitamin B(6), Folate, Vitamin B(12), Pantothenic Acid, Biotin, and Choline.* Washington (DC): National Academies Press (US); 1998.
20. National Health and Medical Research Council (Australia) & New Zealand. Ministry of Health and Australia. Department of Health and Ageing. *Nutrient reference values for Australia and New Zealand : including recommended dietary intakes.* Canberra, A.C.T: National Health and Medical Research Council; 2006.
21. Chuenchor W, Doukov TI, Resto M, Chang A, and Gerratana B. Regulation of the intersubunit ammonia tunnel in Mycobacterium tuberculosis glutamine-dependent NAD+ synthetase. *Biochem J.* 2012;443(2):417-26.
22. Bieganowski P, Pace HC, and Brenner C. Eukaryotic NAD+ synthetase Qns1 contains an essential, obligate intramolecular thiol glutamine amidotransferase domain related to nitrilase. *J Biol Chem.* 2003;278(35):33049-55.
23. Bellinzoni M, Buroni S, Pasca MR, Guglierame P, Arcesi F, De Rossi E, and Riccardi G. Glutamine amidotransferase activity of NAD+ synthetase from Mycobacterium tuberculosis depends on an amino-terminal nitrilase domain. *Res Microbiol.* 2005;156(2):173-7.

24. LaRonde-LeBlanc N, Resto M, and Gerratana B. Regulation of active site coupling in glutamine-dependent NAD(+) synthetase. *Nat Struct Mol Biol.* 2009;16(4):421-9.
25. Wojcik M, Seidle HF, Bieganowski P, and Brenner C. Glutamine-dependent NAD+ synthetase. How a two-domain, three-substrate enzyme avoids waste. *J Biol Chem.* 2006;281(44):33395-402.
26. Rizzi M, Bolognesi M, and Coda A. A novel deamido-NAD+-binding site revealed by the trapped NAD-adenylate intermediate in the NAD+ synthetase structure. *Structure.* 1998;6(9):1129-40.
27. Jauch R, Humm A, Huber R, and Wahl MC. Structures of Escherichia coli NAD synthetase with substrates and products reveal mechanistic rearrangements. *J Biol Chem.* 2005;280(15):15131-40.
28. Dawes R, Bournazos AM, Bryen SJ, Bommireddipalli S, Marchant RG, Joshi H, and Cooper ST. SpliceVault predicts the precise nature of variant-associated mis-splicing. *Nat Genet.* 2023;55(2):324-32.
29. Kasatkar P, Shetty S, and Ghosh K. Genetic heterogeneity in a large cohort of Indian type 3 von Willebrand disease patients. *PLoS One.* 2014;9(3):e92575.
30. Guo DC, Duan XY, Regalado ES, Mellor-Crummey L, Kwartler CS, Kim D, Lieberman K, de Vries BBA, Pfundt R, Schinzel A, et al. Loss-of-Function Mutations in YY1AP1 Lead to Grange Syndrome and a Fibromuscular Dysplasia-Like Vascular Disease. *Am J Hum Genet.* 2017;100(1):21-30.
